# Identification of QTLs related to the vertical distribution and seed-set of pod number in soybean [*Glycine max* (L.) Merri]

**DOI:** 10.1371/journal.pone.0195830

**Published:** 2018-04-17

**Authors:** Hailong Ning, Jiaqi Yuan, Quanzhong Dong, Wenbin Li, Hong Xue, Yanshu Wang, Yu Tian, Wen-Xia Li

**Affiliations:** 1 Key Laboratory of Soybean Biology, Ministry of Education, Harbin, China; 2 Key Laboratory of Soybean Biology and Breeding/Genetics, Ministry of Agriculture, Harbin, China; 3 Soybean Research Institute, Northeast Agricultural University, Harbin, China; College of Agricultural Sciences, UNITED STATES

## Abstract

Pod number is an important factor that influences yield in soybean. Here, we used two associated recombinant inbred line (RIL) soybean populations, RIL3613 (containing 134 lines derived from Dongnong L13 × Heihe 36) and RIL6013 (composed of 156 individuals from Dongnong L13 × Henong 60), to identify quantitative trait loci (QTLs) regulating the vertical distribution and quantity of seeds and seed pods. The numbers of pods were quantified in the upper, middle, and lower sections of the plant, as well as in the plants as a whole, and QTLs regulating these spatial traits were mapped using an inclusive complete interval mapping method. A total of 21 and 26 QTLs controlling pod-number-related traits were detected in RIL3613 and RIL6013, respectively, which explained 1.25–11.6698% and 0.0001–7.91% of the phenotypic variation. A total of 34 QTLs were verified by comparison with previous research, were identified in both populations, or were found to regulate multiple traits, indicating their authenticity. These results enhance our understanding of the vertical distribution of pod-number-related traits and support molecular breeding for seed yield.

## Introduction

The number of pods per plant is one of the most agronomically important traits in soybean [*Glycine max* (L.) Merri], and is strongly positively correlated with yield [1]. Numerous studies have mapped quantitative trait loci (QTLs) for pod number, with the aim of increasing the efficiency of breeding for higher yields [2–18]. Previously, 15 QTLs for total pod number per plant (TPNPP) were identified using recombinant inbred lines (RILs) derived from a cross between BARC-8 and Garimpo [2], while another study identified 12 TPNPP QTLs on chromosomes B1, C2, D1a, F, J, and N using a F_2:10_ RIL population derived from a cross between Charleston and Dongnong 594 [3]. A further nine pod-number QTLs, including two QTLs for the number of pods containing one seed (TPA), one QTL for the number of pods containing two seeds (TPB), two QTLs for three-seed pods (TPC), and four QTLs for the number of four-seed pods (TPD), were identified from a population of introgression lines derived from the donors Harosoy and Clark and the receptor Hongfeng 11 [5]. A similar study using a RIL population of 165 individuals found 11 pod-number QTLs, including one QTL for TPA, five QTLs for TPB, two QTLs for TPC, and three QTLs for TPD [6]. In all, nine QTLs for TPA, six QTLs for TPB, three QTLs for TPC, fifteen QTLs for TPD, and 55 QTLs for TPNPP have been mapped on chromosomes 2–11, 13, and 15–20 (S1 Table).

Soybean pod numbers are also affected by their vertical spatial distribution. The pod numbers in the center of the main stem account for the majority of the pods on each plant, and the seed number in each pod is typically greater in this central region than in pods formed lower on the main stem [19]. In addition, more pods are formed on the upper and central sections of the stem than on the lower sections [20]. These previous findings reveal that pod and seed numbers are not uniform throughout the plant, and understanding the distribution of pods and seeds would be beneficial to improving soybean production.

Previous QTL studies analyzing various aspects of pod numbers in soybean ignored the uneven distribution of pods across the upper, middle, and lower parts of the plant. Physiological research in soybean has revealed that programs to increase pod numbers should consider the differences between the different regions of the plant; therefore, it is imperative to understand the genetic basis of the vertical distribution of pods. In the present study, two soybean RIL populations with a common female parent were used to detect QTLs regulating pod numbers in the upper, middle, and lower parts of the plant. The objective of this paper is to explore the genetic regulation of vertical pod distribution and to identify the QTLs with the largest effects for use in molecular breeding.

## Materials and methods

### Plant materials

Three soybean varieties, Dongnong L13, Henong 60, and Heihe 36, were employed to construct two RIL populations. These three parents derived from germplasms with extensive genetic differences and highly variable pod number traits (Table 1). Two crosses between Dongnong L13 × Henong 60 and between Dongnong L13 × Heihe 36 were conducted in 2008 (E126.63°, N45.75°) in Harbin, Heilngjiang, China, and 15 and 22 hybrid seeds were harvested for the two crosses, respectively. The F_1_ seeds were sown in Yacheng (E109.00°, N17.5°) in Hainan Province, China, and the mature plants were harvested in the winter. The non-hybrid seeds were removed following a comparison with the female parent, and the remaining seeds were continuously self-crossed for five generations from 2010 to 2013, in Harbin in the summer and Yacheng in the winter, with each individual selected from single-seed descent. A total of 134 and 156 RILs were ultimately obtained for the two populations, named RIL3613 (Dongnong L13 × Heihe 36) and RIL6013 (Dongnong L13 × Henong 60) respectively, which were used for the construction of the genetic linkage map and QTL mapping in the present study.

**Table 1:** Descriptive analysis of 16 pod-number-related traits in RIL3613 and RIL6013.

Trait ^A^	Parents		RIL3613 ^B^					
	Dongnong L13	Heihe 36	Mean	Standard Error	Skewness	Kurtosis	Minimum	Maximum
PNUA	2	2	1.672	0.896	1.096	0.116	1	4
PNMA	3	3	1.824	1.251	1.576	1.452	1	6
PNBA	2	1	1.528	0.779	1.451	2.046	1	5
PNUB	4	5	3.672	1.963	0.882	0.737	1	11
PNMB	4	8	4.728	2.827	0.573	-0.627	1	12
PNBB	5	6	3.712	2.055	0.492	-0.772	1	9
PNUC	14	7	5.800	2.865	1.020	1.221	1	16
PNMC	14	5	8.256	4.160	0.697	0.131	1	21
PNBC	4	5	5.328	3.169	0.920	0.598	1	16
PNUD	1	2	2.496	2.235	2.047	5.281	1	14
PNMD	1	1	2.704	2.750	2.992	12.759	1	20
PNBD	1	1	1.816	1.547	2.593	7.709	1	10
TPA	8	6	5.024	1.978	0.928	0.203	3	11
TPB	13	19	12.112	5.303	0.410	-0.798	3	24
TPC	32	17	19.384	8.130	0.611	-0.031	6	43
TPD	3	4	7.016	5.468	2.124	5.777	3	35
Trait ^A^	Parents				RIL6013			
	Dongnong L13	Henong60	Mean	Standard Error	Skewness	Kurtosis	Minimum	Maximum
PNUA	2	1	1.855	1.349	2.497	7.923	1	9
PNMA	3	1	2.069	1.403	1.468	1.696	1	7
PNBA	2	1	1.828	1.282	3.321	17.973	1	11
PNUB	4	3	4.731	2.777	1.242	1.629	1	16
PNMB	4	3	6.021	3.546	0.901	0.488	1	18
PNBB	5	2	4.241	2.325	0.803	0.486	1	13
PNUC	14	3	5.628	2.908	1.078	1.582	1	17
PNMC	14	5	7.262	3.350	0.960	2.312	1	22
PNBC	4	4	4.835	2.595	1.467	4.034	1	18
PNUD	1	2	1.469	1.185	3.129	10.076	1	7
PNMD	1	1	1.724	1.689	3.534	15.125	1	12
PNBD	1	1	1.255	0.806	3.978	17.287	1	6
TPA	8	3	5.752	2.737	1.171	1.018	3	16
TPB	13	8	14.993	7.272	0.983	0.933	4	39
TPC	32	12	17.724	7.025	1.277	4.169	5	54
TPD	3	4	4.448	3.140	3.282	13.489	3	24

A: PNUA, number of pods containing one seed in the upper part of the plant; PNMA, number of pods containing one seed in the middle plant section; PNBA, number of pods containing one seed in the lower part of the plant; PNUB, number of pods containing two seeds in the upper part of the plant; PNMB, number of pods containing two seeds in the middle plant section; PNBB, number of pods containing two seeds in the lower part of the plant; PNUC, number of pods containing three seeds in the upper part of the plant; PNMC, number of pods containing three seeds in the middle plant section; PNBC, number of pods containing three seeds in the lower part of the plant; PNUD, number of pods containing four seeds in the upper part of the plant; PNMD, number of pods containing four seeds in the middle plant section; PNBD, number of pods containing four seeds in the lower part of the plant; TPA, total number of pods containing one seed; TPB, total number of pods containing two seeds; TPC, total number of pods containing three seeds; TPD, total number of pods containing four seeds.

B: RIL3613 and RIL6013 are RILs derived from Dongnong L13 × Heihe 36 and Dongnong L13 × Henong 60, respectively.

### Field experiment

The parental lines and RILs were planted in Harbin in 2015, and were grown in a randomized complete block design with three replications. Each plot contained three 3-m rows that were 70 cm apart, and the seeds of an individual line were sown at 6-cm intervals. The field experiment was managed identically to the local soybean crops.

### Trait evaluations

Five mature plants were selected randomly from the middle row of each plot and the number of pods containing one, two, three, and four seeds on each node of the main stem were recorded. The number of nodes on the main stem was divided by three, with the top, central, and lower thirds of the plants labeled as the upper, middle, and lower sections of the plant, respectively. If dividing the number of nodes by three left a remainder of 1, the extra node was allocated to the middle section. If the remainder was two, one additional node was allocated to the top section and the other was allocated to the lower section.

For the upper section, the total number of pods containing one, two, three, and four seeds were recorded as PNUA, PNUB, PNUC, and PNUD, respectively. In the middle section, these categories were labeled PNMA, PNMB, PNMC, and PNMD, and in the lower part of the plant they were recorded as PNBA, PNBB, PNBC, and PNBD. The total number of pods containing one, two, three, or four seeds on all nodes of the plant were recorded as TPA, TPB, TPC, and TPD, respectively.

### SSR marker analysis

Juvenile leaves were collected from the two RIL populations, frozen in liquid nitrogen, then immediately ground into powder. Total genomic DNA was extracted using the CTAB method [21], and eluted in 50 μl deionized water. Its concentration was determined using a UV752N spectrophotometer (Shanghai Jingke Science Instrument Co. Ltd.) and was diluted to 100 ng^–1^ in deionized water.

A total of 560 simple sequence repeat (SSR) markers evenly distributed across the soybean genome [22] were selected to screen for polymorphisms between the two parents of each RIL population. Of these, 137 and 150 primer pairs showed polymorphisms in RIL6013 and RIL3613, respectively, and were therefore used for SSR genotyping. An optimized PCR was performed using a total reaction volume of 20 μl, including 3 μl genomic DNA, 2 μl reaction buffer, 3 μl SSR primer, 0.3 μl dNTP, 0.2 μl Taq DNA polymerase, and 11.5 μl ddH_2_O. The reaction was performed at 94°C for 10 min; followed by 38 cycles of 94°C for 30 s, 50°C for 30 s, and 72°C for 30 s; with a final extension at 72°C for 10 min. A 6% denaturing polyacrylamide gel electrophoresis was used for silver staining, water extraction, development, and genotyping.

### Construction of the linkage map and QTL analysis

The linkage maps were constructed using the QTLIciMapping 4.0 software (www.isbreeding.net), using the default setting for all parameters.

The average number of pods from five individuals per line was used for statistical analysis. The inclusive compositive interval mapping method (ICIM) [23], compositive interval mapping method (CIM) and single marker analysis (SMA) [24]. The significant threshold of LOD (logarithm of odds) score for ICIM, LR (likelihood ratio) for CIM and probability over F value for SMA were set as 2.5, 11.5 and 0.05, respectively. When the QTL were detected simultaneously by over two methods, and PVE (phenotypic variation explanation ratio) for ICIM over 2% or R^2^ (coefficient of determination) for CIM over 0.1, we declared the presence of the QTL. ICIM was implemented by the IciMapping 4.0 software (www.isbreeding.net), and CIM and SMA were implemented by the WinQTLCart 2.5 software (https://brcwebportal.cos.ncsu.edu/qtlcart/WQTLCart.htm).

All data obtained from experiment were listed in S1 Dataset.

## Results

### Phenotypic analysis

For all 16 pod number traits, there was a large variation among the RIL3613 and RIL6013 lines (Table 1 and S1 Fig); therefore, the two RIL populations were suitable for use to detect QTLs for PNUA, PNMA, PNBA, PNUB, PNMB, PNBB, PNUC, PNMC, PNBC, PNUD, PNMD, PNBD, TPA, TPB, TPC, and TPD. In terms of the pod number-related seed set traits, those related to three seeds per pod (PNUC, PNMC, PNBC) showed the largest means and ranges in both populations, while those related to pods with one seed (PNUA, PNMA, PNBA) exhibited the lowest mean and ranges in both populations. PNUD, PNMD, PNBD showed the second largest range in RIL3613, while PNUB, PNMB, PNBB had second biggest range in RIL6013. Importantly, there were parallel differences between populations in vertical distribution of numbers of pods, with the mean and range of middle part being the largest, and those of the upper and lower regions being approximately equal. In all, these data showed that there was high variation by pod number in seed set and vertical distribution, and different QTLs associated with pod number could likely be identified based on seed set and vertical distribution.

### Linkage map

A total of 150 and 137 SSR markers were anchored across all 20 soybean chromosomes in RIL3613 and RIL6013, with total linkage map lengths of 2849.54 cM and 1886.8 cM, respectively. The mean interval lengths for RIL3613 and RIL6013 populations were 21.92 cM and 16.13 cM, respectively. For RIL3613, the length of each linkage group ranged from 1.15 cM to 283.42 cM, with 31 intervals (23.85%) shorter than 10 cM, and 20 intervals (15.38%) longer than 25 cM. For RIL6013, the linkage group length varied from 19.68 cM to 163.67 cM, with 30 intervals (26.50%) shorter than 10 cM, and 14 intervals (11.97%) longer than 25 cM (S2 Table, S2 and S3 Figs).

### QTL mapping of the pod-number traits

A total of 47 QTLs were found to be associated with PNUA, PNUD, PNMA, PNMB, PNMC, PNMD, PNBA, PNBB, PNBC, PNBD, TPA, TPB, TPC, and TPD (Table 2). Of these, 21 and26 were identified from RIL3613 and RIL6013, respectively.

**Table 2:** QTLs associated with pod-number-related traits detected in RIL3613 and RIL6013.

QTL	Method^A^	Marker interval	Genomic region^B^	Trait^C^	LOD^D^	PVE (%)^E^	R^2^ ^F^	Additive effects	Population	Re-identification
*qPN-D1a-1*	ICIM	satt482~satt254	45.75~56.43 cM	PNBD	21.99	0.84		-1.46	RIL6013	*qPN-D1a-2* in RIL3613
	CIM			PNBD	19.27		0.57	-1.35	RIL6013	
	ICIM			PNUA	8.29	1.04		-2.14	RIL6013	
	CIM			PNUA	5.53		0.45	-1.96	RIL6013	
*qPN-D1a-2*	ICIM	Sat_346~Satt198	53.66~68.62 cM	PNMD	6.10	2.70		-6.58	RIL3613	*qPN-D1a-1* in RIL6013
	CIM			PNMD	8.62		0.01	-6.35	RIL3613	
	SMA	Sat_346	53.66 cM	PNMD	0.95		0.03	-0.85	RIL3613	
	ICIM			TPD	3.45		1.93	-9.00	RIL3613	
	CIM			TPD	4.39		0.36	0	RIL3613	
*qPN-D1b-1*	ICIM	sat_096~sat_289	0~131.91 cM	PNBD	23.32	0.84		-1.46	RIL6013	[9][13]
	CIM			PNBD	12.56		0.50	-0.98	RIL6013	*qPN-D1b-3* in RIL3613
	ICIM			PNUA	7.36	1.07		-1.96	RIL6013	
	CIM			PNUA	5.57		0.49	-1.73	RIL6013	
*qPN-D1b-2*	CIM	satt546~staga002	87.19~126.44 cM	PNBD	18.13		0.56	-1.35	RIL6013	*qPN-D1b-3* in RIL3613
	ICIM			PNBD	19.79	0.84		-1.46	RIL6013	
	CIM			PNMA	3.08		0.41	-1.25	RIL6013	
	ICIM			PNMA	3.79	2.11		-1.44	RIL6013	
	SMA	staga002	126.44 cM	PNMC	2.58		0.08	-1.85	RIL6013	
	ICIM			PNMC	2.57	7.91		-1.85	RIL6013	
*qPN-D1b-3*	ICIM	Sat_069~Sat_183	102.59~112.62 cM	TPA	2.51	10.21		-1.02	RIL3613	*qPN-D1b-1*, *qPN-D1b-2* in RIL6013
	SMA	Sat_069	102.59 cM	TPA	5.17		0.04	-0.42	RIL3613	
*qPN-N-1*	ICIM	Sat_166~satt237	38.59~74.98 cM	PNBA	9.11	5.84		-3.17	RIL6013	[14]
	CIM			PNBA	37.28		0.29	-3.11	RIL6013	
	ICIM			PNBD	27.07	0.84		-1.46	RIL6013	
	CIM			PNBD	110.37		0.56	-1.35	RIL6013	
	ICIM			PNMD	11.50	0.97		-2.54	RIL6013	
	CIM			PNMD	55.33		0.40	-3.15	RIL6013	
	SMA	satt237	74.98 cM	PNMD	5.58	0.04		-0.40	RIL6013	
	ICIM			PNUA	6.58	1.00		-2.08	RIL6013	
	CIM			PNUA	32.57		0.44	-1.81	RIL6013	
	ICIM			TPD	8.55	2.73		-3.96	RIL6013	
	CIM			TPD	28.61		0.02	0.00	RIL6013	
*qPN-C1-1*	ICIM	Satt396~Sat_140	24.11~41.43 cM	PNBB	3.15	3.30		-0.94	RIL3613	[5][12]
	CIM			PNBB	5.24		0.25	-1.15	RIL3613	*qPN-C1-2* in RIL6013
	SMA	Satt396	24.11 cM	PNBB	1.74		0.06	-0.63	RIL3613	
	SMA	Sat_140	41.43cM	PNBB	2.71		0.09	-0.68	RIL3613	
	SMA	Satt396	24.11 cM	PNUA	0.95		0.03	-0.20	RIL3613	
	SMA	Sat_140	41.43cM	PNUA	1.81		0.07	-0.25	RIL3613	
	CIM			TPA	2.57		0.13	-0.87	RIL3613	
	SMA	Satt396	24.11 cM	TPA	1.30		0.03	-0.53	RIL3613	
	SMA	Sat_140	41.43cM	TPA	1.73		0.07	-0.53	RIL3613	
*qPN-C1-2*	ICIM	sat_367~sat_140	28.04~41.43 cM	PNBA	4.92	2.84		-0.64	RIL6013	[5][12]
	CIM			PNBA	3.85		0.13	-0.57	RIL6013	*qPN-C1-1* in RIL3613
	SMA	sat_367	28.04 cM	PNBA	1.94		0.05	-0.40	RIL6013	
	SMA	sat_140	41.43 cM	PNBA	3.20		0.10	-0.50	RIL6013	
*qPN-C1-3*	CIM	Sat_140~Sat_416	41.43~76.41 cM	PNUA	6.99		0.56	-0.73	RIL3613	[2][5][14]
	SMA	Sat_416	76.41 cM	PNUA	0.99		0.03	-0.17	RIL3613	
*qPN-A1-1*	ICIM	Satt717~Sat_171	51.95~57.79 cM	PNMA	12.00	1.88		-1.48	RIL3613	
	CIM			PNMA	8.54		0.59	-1.36	RIL3613	
*qPN-A1-2*	ICIM	SOYNOD26A~Sat_171	57.24~57.79 cM	PNBD	7.21	1.65		2.11	RIL3613	
	CIM			PNBD	10.03		0.50	2.28	RIL3613	
	ICIM			PNMD	4.41	2.80		4.11	RIL3613	
	CIM			PNMD	4.23		0.33	3.38	RIL3613	
*qPN-C2-1*	ICIM	Satt277~Satt289	107.58~112.34 cM	PNMA	4.30	1.45		-1.66	RIL3613	[3][6][7]
	CIM			PNMA	5.67		0.37	-1.59	RIL3613	*qPN-C2-2* in RIL6013
*qPN-C2-2*	ICIM	satt376~satt307	97.83~121.26 cM	PNBD	19.43	0.84		-1.46	RIL6013	[3][6] [7] [12] [15][16]
	CIM			PNBD	17.67		0.61	-1.37	RIL6013	*qPN-C2-1*in RIL3613
	SMA	satt307	121.26 cM	PNBD	1.72		0.05	-0.30	RIL6013	
*qPN-C2-3*	ICIM	satt307~satt202	121.26~126.23 cM	PNBD	21.38	0.84		-1.46	RIL6013	[15]
	CIM			PNBD	17.14		0.61	-1.37	RIL6013	
*qPN-M-1*	ICIM	Sat_389~Satt697	0.00~85.34 cM	PNBA	3.70	4.65		0.69	RIL3613	[5]
	SMA	Satt697	85.34 cM	PNBA	2.20		0.08	0.23	RIL3613	
	CIM			TPA	2.77		0.15	0.80	RIL3613	
	SMA	Satt697	85.34 cM	TPA	2.02		0.07	0.55	RIL3613	
*qPN-M-2*	ICIM	Satt626~Satt536	58.59~62.13 cM	PNMB	2.78	2.19		-0.90	RIL3613	
	CIM			PNMB	2.57		0.08	-0.83	RIL3613	
	SMA	Satt626	58.59 cM	PNMB	1.37		0.05	-0.63	RIL3613	
	SMA	Satt536	62.13 cM	PNMB	2.29		0.08	-0.81	RIL3613	
*qPN-A2-1*	ICIM	sat_406~satt424	25.90~60.59 cM	PNBD	19.11	0.84		-1.46	RIL6013	[5][13][14]
	CIM			PNBD	16.72		0.56	-1.35	RIL6013	
*qPN-K-1*	ICIM	satt673~Sat_243	50.79~86.77 cM	PNBD	3.67	0.28		-0.53	RIL6013	[2]
	CIM			PNBD	3.51		0.25	-0.57	RIL6013	
	SMA	satt673	50.79 cM	PNBD	1.24		0.04	-0.23	RIL6013	
*qPN-O-1*	ICIM	Satt500~Satt153	14.17~118.13 cM	PNMD	5.53	2.69		-6.45	RIL3613	[5][9][14][15]
	CIM			PNMD	4.15		0.22	-4.67	RIL3613	*qPN-O-2* in RIL6013
*qPN-O-2*	ICIM	satt479~Sat_341	54.2~67.93 cM	PNBD	24.40	0.84		-1.46	RIL6013	[14][15]
	CIM			PNBD	21.23		0.58	-1.36	RIL6013	*qPN-O-1* in RIL3613
	ICIM			PNMD	10.64	0.98		-2.59	RIL6013	
	CIM			PNMD	10.25		0.41	-3.11	RIL6013	
	CIM			PNUA	8.48		0.43	-1.92	RIL6013	
	SMA	satt479	54.2 cM	PNUA	1.15		0.04	-0.70	RIL6013	
	ICIM			TPD	10.00	2.68		-4.11	RIL6013	
	CIM			TPD	7.37		0.00	0.00	RIL6013	
*qPN-O-3*	ICIM	Satt153~Satt243	118.13~119.50 cM	PNBB	3.41	11.67		1.62	RIL3613	[5][9]
	SMA	Satt243	119.5 cM	PNBB	1.07		0.04	0.94	RIL3613	
*qPN-O-4*	ICIM	Satt243~Sat_307	119.50~123.43 cM	PNMB	3.32	10.70		2.26	RIL3613	[5][9]
	CIM			PNMB	3.00		0.46	2.36	RIL3613	
*qPN-B1-2*	ICIM	BE806308~sat_272	0.00~14.32 cM	PNBD	18.99	0.84		-1.47	RIL6013	
	SMA	sat_272	14.32 cM	PNBD	1.62		0.05	-0.39	RIL6013	
	ICIM			PNMD	9.81	0.99		-3.28	RIL6013	
	SMA	sat_272	14.32 cM	PNMD	3.56		0.11	-1.20	RIL6013	
	ICIM			PNUD	20.89	0.90		-2.26	RIL6013	
	CIM			PNUD	18.44		0.56	-2.16	RIL6013	
	SMA	sat_272	14.32 cM	PNUD	1.82		0.06	-0.61	RIL6013	
	ICIM			TPD	7.28	1.83		-6.59	RIL6013	
	SMA	sat_272	14.32 cM	TPD	3.47		0.10	-2.21	RIL6013	
*qPN-B1-3*	ICIM	sat_272~satt583	14.32~84.19 cM	PNMD	14.53	1.06		-3.56	RIL6013	[3][13]
	CIM			PNMD	15.02		0.53	-3.41	RIL6013	
	ICIM			TPD	9.78	1.98		-8.89	RIL6013	
	CIM			TPD	8.63		0.27	-4.16	RIL6013	
*qPN-B1-1*	ICIM	satt197~sat_123	46.38~100.87 cM	PNBA	10.72	5.84		-3.17	RIL6013	[3][13]
	CIM			PNBA	9.53		0.29	-3.11	RIL6013	
	SMA	Satt197	46.38 cM	PNBA			0.03	-0.14	RIL3613	
	SMA	satt197	46.38 cM	PNBA	0.85		0.03	-0.25	RIL6013	
	ICIM			PNBC	4.26	1.34		-3.46	RIL6013	
	CIM			PNBC	5.66		0.35	-3.53	RIL6013	
	SMA	sat_123	100.87 cM	PNBC	1.24		0.04	-1.79	RIL6013	
	ICIM			PNMD	10.71	0.99		-2.52	RIL6013	
	CIM			PNMD	10.24		0.40	-3.11	RIL6013	
	ICIM			PNUA	12.38	1.05		-2.12	RIL6013	
	CIM			PNUA	10.48		0.49	-2.07	RIL6013	
	SMA	sat_123	100.87 cM	PNUA	2.35		0.07	-1.27	RIL6013	
*qPN-B1-4*	ICIM	satt583~satt359	84.19~102.55 cM	PNBD	26.83	0.84		-1.46	RIL6013	
	CIM			PNBD	25.88		0.64	-1.39	RIL6013	
	SMA	satt583	84.19 cM	PNBD	1.11		0.03	-0.33	RIL6013	
	SMA	satt359	102.55 cM	PNBD	1.14		0.04	-0.17	RIL6013	
	ICIM			PNMD	11.97	0.99		-2.57	RIL6013	
	CIM			PNMD	14.70		0.53	-3.35	RIL6013	
	SMA	satt583	84.19 cM	PNMD	1.30		0.04	-0.74	RIL6013	
	ICIM			PNUD	23.13	0.91		-2.19	RIL6013	
	CIM			PNUD	20.69		0.58	-1.84	RIL6013	
	SMA	satt359	102.55 cM	PNUD	1.11		0.03	-0.25	RIL6013	
	ICIM			TPD	13.23	2.73		-3.96	RIL6013	
	CIM			TPD	11.08		0.53	-4.16	RIL6013	
	SMA	satt583	84.19 cM	TPD	1.25		0.04	-1.35	RIL6013	
	SMA	satt359	102.55 cM	TPD	0.90		0.03	-0.59	RIL6013	
*qPN-H-1*	ICIM	satt293~Satt181	89.08~91.12 cM	PNBA	2.89	1.26		0.38	RIL6013	
	CIM			PNBA	3.60		0.08	0.43	RIL6013	
*qPN-F-1*	ICIM	GMRUBP~Sat_262	0.00~9.69 cM	PNMA	13.34	1.85		-1.59	RIL3613	
	CIM			PNMA	9.03		0.56	-1.59	RIL3613	
*qPN-B2-1*	ICIM	Sct_094~Satt063	70.55~93.48 cM	PNBD	9.45	1.62		-2.23	RIL3613	
	CIM			PNBD	8.66		0.50	-2.20	RIL3613	
	ICIM			PNMD	4.72	2.77		-5.02	RIL3613	
	CIM			PNMD	3.75		0.31	-4.27	RIL3613	
	ICIM			TPD	4.95	1.95		-9.01	RIL3613	
	CIM			TPD	5.06		0.47	0.00	RIL3613	
*qPN-E-3*	ICIM	sat_136~satt651	32.09~39.16 cM	PNBA	2.85	3.40		-1.29	RIL6013	
	CIM			PNBA	5.56		0.29	-3.11	RIL6013	
	ICIM			PNUD	20.82	0.91		-2.21	RIL6013	
	CIM			PNUD	17.69		0.53	-2.20	RIL6013	
*qPN-E-1*	ICIM	satt685~satt231	56.70~70.23 cM	PNMA	4.77	2.23		-1.48	RIL6013	[13]
	CIM			PNMA	3.42		0.51	-1.35	RIL6013	
*qPN-E-2*	CIM	Satt553~Satt231	67.91`70.23 cM	PNMD	3.29		0.10	0.89	RIL3613	
	SMA	Satt553	67.91 cM	PNMD	2.53		0.10	0.84	RIL3613	
	SMA	Satt231	70.23 cM	PNMD	0.97		0.04	0.52	RIL3613	
*qPN-J-1*	ICIM	sat_228~Sat_366	23.91~52.84 cM	PNBC	4.25	0.72		-6.54	RIL6013	[13]
	CIM			PNBC	3.04		0.24	-3.87	RIL6013	
*qPN-J-2*	ICIM	Sat_366~sat_394	52.84~89.43 cM	PNUA	11.71	0.96		-2.33	RIL6013	
	CIM			PNUA	8.76		0.43	-2.06	RIL6013	
*qPN-D2-1*	ICIM	Sat_333~Sct_192	5.83~11.77 cM	PNBD	6.80	1.75		2.03	RIL3613	
	CIM			PNBD	4.52		0.60	1.91	RIL3613	
	CIM			PNMC	2.82		0.02	1.78	RIL3613	
	SMA	Sat_333	5.83 cM	PNMC	1.26		0.04	0.95	RIL3613	
*qPN-D2-2*	ICIM	Sct_192~Sat_284	11.77~30.79 cM	PNBD	8.99	1.65		-2.02	RIL3613	[12]
	CIM			PNBD	6.04		0.54	-1.89	RIL3613	
	ICIM			PNMA	4.52	1.74		-1.43	RIL3613	
	CIM	Sct_192~Sat_284	11.77~30.79 cM	PNMA	4.86		0.59	-1.31	RIL3613	
*qPN-G-4*	ICIM	satt352~satt564	50.52~57.32 cM	PNBD	24.79	0.84		-1.47	RIL6013	[13]
	CIM			PNBD	21.35		0.63	-1.41	RIL6013	
	SMA	satt352	50.52 cM	PNBD	2.23		0.07	-0.64	RIL6013	
	ICIM			PNMD	13.86	0.92		-2.71	RIL6013	
	CIM			PNMD	13.15		0.39	-2.47	RIL6013	
	SMA	satt564	57.32 cM	PNMD	1.37		0.04	-0.59	RIL6013	
	ICIM			TPD	9.10	2.37		-4.24	RIL6013	
	CIM			TPD	7.88		0.37	-3.74	RIL6013	
	SMA	satt564	57.32 cM	TPD	0.92		0.03	-0.90	RIL6013	
*qPN-G-3*	ICIM	satt352~sat_117	50.52~100.00 cM	PNUA	7.58	1.00		-2.28	RIL6013	[6][13]
	CIM			PNUA	5.60		0.42	-2.04	RIL6013	*qPN-G-1* in RIL3613
	ICIM			PNUD	20.05	0.89		-2.27	RIL6013	
	CIM			PNUD	16.15		0.53	-2.25	RIL6013	
*qPN-G-1*	CIM	Sat_203~Satt503	62.08~68.76 cM	TPD	2.61		0.10	0.00	RIL3613	qPN-G-3 in RIL6013
	SMA	Sat_203	62.08 cM	TPD	1.01		0.04	-1.10	RIL3613	
*qPN-G-2*	CIM	sat_210~satt309	3.7~4.53 cM	PNMB	4.17		0.11	-1.57	RIL6013	
	SMA	satt309	4.53 cM	PNMB	2.01		0.06	-0.96	RIL6013	
	CIM	sat_210~satt309	3.7~4.53 cM	TPB	4.47		0.12	-3.01	RIL6013	
	SMA	satt309	4.53 cM	TPB	2.26		0.07	-2.09	RIL6013	
	CIM	sat_210~satt309	3.7~4.53 cM	TPC	4.92		0.13	-3.17	RIL6013	
	SMA	satt309	4.53 cM	TPC	1.23		0.04	-1.50	RIL6013	
*qPN-L-2*	ICIM	sat_405~sat_195	29.62~30.83 cM	PNBD	23.85	0.84		-1.46	RIL6013	[13]
	CIM			PNBD	20.91		0.56	-1.35	RIL6013	
*qPN-L-3*	ICIM	sat_195~satt448	30.83~64.66 cM	PNBC	3.94	1.76		-3.06	RIL6013	[9][13]
	CIM			PNBC	3.58		0.31	-2.87	RIL6013	*qPN-L-1* in RIL3613
	ICIM			PNMD	11.43	0.97		-2.57	RIL6013	
	CIM			PNMD	11.50		0.40	-3.20	RIL6013	
	ICIM			PNUD	24.35	0.90		-2.23	RIL6013	
	CIM			PNUD	20.43		0.53	-2.23	RIL6013	
	ICIM			TPD	9.04	2.32		-4.45	RIL6013	
	CIM			TPD	8.17		0.39	-4.67	RIL6013	
*qPN-L-1*	ICIM	Satt497~Sat_099	33.70~78.23 cM	PNMA	15.92	1.86		-1.57	RIL3613	[9][13]
	CIM			PNMA	12.78		0.61	-1.46	RIL3613	*qPN-L-3* and *qPN-L-4* in RIL6013
*qPN-L-4*	CIM	satt313~satt373	34.54~107.23 cM	PNMC	4.14		0.11	1.31	RIL6013	[5] [8][9] [13]
	SMA	satt373	107.23 cM	PNMC	1.88		0.06	0.86	RIL6013	*qPN-L-1* in RIL3613
*qPN-I-2*	ICIM	satt571~satt367	18.50~27.98 cM	PNMA	4.93	1.96		-1.72	RIL6013	*qPN-I-1*, *qPN-I-3* in RIL3613
	CIM			PNMA	3.16		0.40	-1.66	RIL6013	
	ICIM			PNMD	11.20	0.98		-2.59	RIL6013	
	CIM			PNMD	9.06		0.43	-2.61	RIL6013	
	ICIM			PNUD	18.02	0.91		-2.21	RIL6013	
	CIM			PNUD	13.75		0.54	-1.88	RIL6013	
	ICIM			TPD	8.34	2.69		-4.06	RIL6013	
	CIM			TPD	4.51		0.33	0.00	RIL6013	
*qPN-I-1*	ICIM	Satt571~Satt292	18.50~82.77 cM	PNUA	6.00	2.06		-0.86	RIL3613	[6] [12] [14]
	CIM			PNUA	5.66		0.52	-0.75	RIL3613	*qPN-I-2* in RIL6013
	SMA	Satt292	82.77 cM	PNUA	0.94		0.03	-0.18	RIL3613	
*qPN-I-3*	ICIM	Satt571~GMGLPSI2	18.50~97.04 cM	PNBA	3.53	2.16		-0.67	RIL3613	[6][12][14]
	CIM			PNBA	4.49		0.33	-0.70	RIL3613	*qPN-I-2* in RIL6013
	SMA	GMGLPSI2	97.04 cM	PNBA	2.20		0.08	-0.31	RIL3613	
	ICIM			TPA	2.92	6.51		-1.18	RIL3613	
	SMA	GMGLPSI2	97.04 cM	TPA	2.70		0.09	-0.86	RIL3613	

A: ICIM, the inclusive compositive interval mapping method; CIM, compositive interval mapping method; SMA, single marker analysis.

B: Interval in public map [22].

C: PNUA, number of pods containing one seed in the upper part of the plant; PNMA, number of pods containing one seed in the middle plant section; PNBA, number of pods containing one seed in the lower part of the plant; PNUB, number of pods containing two seeds in the upper part of the plant; PNMB, number of pods containing two seeds in the middle plant section; PNBB, number of pods containing two seeds in the lower part of the plant; PNUC, number of pods containing three seeds in the upper part of the plant; PNMC, number of pods containing three seeds in the middle plant section; PNBC, number of pods containing three seeds in the lower part of the plant; PNUD, number of pods containing four seeds in the upper part of the plant; PNMD, number of pods containing four seeds in the middle plant section; PNBD, number of pods containing four seeds in the lower part of the plant; PVE, phenotypic variation explained; TPA, total number of pods containing one seed; TPB, total number of pods containing two seeds; TPC, total number of pods containing three seeds; TPD, total number of pods containing four seeds.

D: LOD, logarithm of odds.

E: PVE, phenotypic variation explanation ratio from ICIM via QTL IciMapping 4.1.

F: R^2^, coefficient of determination obtained from CIM and SMA via WinQTLCart 2.5.

#### QTLs for the numbers of one-seed pods

Two QTLs for PNUA (*qPN-C1-3*, and *qPN-I-1*) were detected in RIL3613. At these two QTLs, the alleles from Heihe 36 could increase PNUA. In RIL6013, seven PNUA QTLs (*qPN-D1a-1*, *qPN-D1b-1*, *qPN-N-1*, *qPN-O-2*, *qPN-B1-1*, *qPN-J-2*, and *qPN-G-3*) were detected, The additive effects of these seven QTLs were negative, indicating that the alleles that increased PNUA derived from Henong 60.

A total of five QTLs for PNMA (*qPN-A1-1*, *qPN-C2-1*, *qPN-F-1*, *qPN-D2-2*, and *qPN-L-1*), located in linkage groups (LGs) A1, C2, A2, F, D2 and L, were detected in RIL3613. The alleles that increased PNMA were carried by Heihe 36. In RIL6013, three QTLs (*qPN-D1b-2*, *qPN-E-1* and *qPN-I-2*) underlying PNMA were detected in LGs D1b, E and I,. Alleles expressing positive additive effects on PNMA were derived from Henong 60 at all three of these QTLs.

Two QTLs (*qPN-M-1* and *qPN-I-3*) controlling 4.65% and 2.16% of the phenotypic variation in PNBA were detected in RIL3613 in LGs M, and I. The Dongnong L13 allele for *qPN-M-1* and the Heihe 36 alleles for *qPN-I-3* enhanced PNBA. Six QTLs associated with PNBA (*qPN-N-1*, *qPN-C1-2*, *qPN-B1-1*, *qPN-H-1*, and *qPN-E-3*) were detected in LGs N, C1, B1, H, and E in RIL6013. The Henong 60 alleles for five QTLs (*qPN-N-1*, *qPN-C1-2*, *qPN-B1-1*, and *qPN-E-3*) and the *qPN-H-1* allele from Dongnong L13 increased PNBA.

Four QTLs (*qPN-D1b-3*, *qPN-C1-1*, *qNP-M-1* and *qNP-I-3*) for TPA were located in LGs D1b, C1, M and I in RIL3613. The additive effects of QTL *qPN-D1b-3*, *qPN-C1-1*, and *qNP-I-3* were negative, indicating that the alleles carried by Heihe 36 could improve TPA. The additive effects of QTL *qPN-M-1* were positive which showed that the alleles carried by Dongnong L13 could improve TPA.

#### QTLs for the numbers of two-seed pods

Two QTLs (*qPN-M-2* and *qPN-O-4*) for PNMB were detected in RIL3613, which explained 2.12% and 10.70% of the phenotypic variation, respectively. Synergistic alleles for *qPN-O-4* and *qPN-M-2* for PNMB were carried by Dongnong L13 and Heihe 36, respectively. One QTLs (*qPN-G-2*) for PNMB were detected in RIL6013, in which synergistic allele were carried by Henong 60. Two QTLs (*qPN-C1-1* and *qPN-O-3*) associated with PNBB were discovered in RIL3613, which explained 3.30% and 11.67% of the phenotypic variation, respectively. The *qPN-O-3* allele with a positive additive effect on PNBB was carried by Dongnong L13, while that of *qPN-C1-1* derived from Heihe 36.

One TPB QTLs (*qPN-G-2*) were detected in RIL6013, and explained 12% (*R*^2^) of the phenotypic variation. The Henong 60 allele for *qPN-G-2* and the Dongnong L13 allele for *qPN-O-4* increased TPB.

#### QTLs for the numbers of three-seed pods

Two QTL (*qPN-D1b-2* and *qPN-L-4*) explained 7.91% and 11% of the PNMC phenotypic variation in RIL6013, and the allele providing the positive additive effect was derived from Henong 60 and Dongnong L13, respectively. Three PNBC QTLs (*qPN-B1-1*, *qPN-J-1*, and *qPN-L-3*) detected in RIL6013, and the alleles of these three QTLs that could increase PNBC were derived from Henong 60.

One TPC QTL (*qPN-G-2*) was detected in RIL6013, and explained just 13% (R^2^) of the phenotypic variation. The negative effect of this QTL demonstrated that the Henong 60 allele for this QTL increased TPC.

#### QTLs for the numbers of four-seed pods

In RIL6013, six QTLs associated with PNUD (*qPN-B1-3*, *qPN-B1-4*, *qPN-E-3*, *qPN-G-3*, *qPN-L-3*, and *qPN-I-2*) were identified, and the Henong 60 alleles for all 14 of these QTLs increased PNUD.

Five QTLs (*qPN-D1a-2*, *qPN-A1-2*, *qPN-O-1*, *qPN-B2-1* and *qPN-E-2*) for PNMD detected in RIL3613. The Heihe 36 alleles for *qPN-D1a-2*, *qPN-O-1*, and *qPN-B2-1*, the Dongnong L13 allele of *qPN-A1-2* and *qPN-E-2*, increased PNMD. In RIL6013, nine QTLs associated with PNMD (*qPN-N-1*, *qPN-O-2*, *qPN-B1-2*, *qPN-B1-3*, *qPN-B1-1*, *qPN-B1-4*, *qPN-G-4*, *qPN-L-3*, and *qPN-I-2*), and the additive effects of these nine QTLs were negative, indicating that the alleles that increase PNMD derived from Henong 60.

Four QTLs underlying PNBD (*qPN-A1-2*, *qPN-B2-1*, *qPN-D2-1*, and *qPN-D2-2*) were detected in RIL3613. Alleles of *qPN-A1-2* and *qPN-D2-1* from Dongnong L13 and those of *qPN-B2-1*, and *qPN-D2-2* from Heihe 36 improved PNBD. A total of 13 PNBD QTLs (*qPN-D1a-1*, *qPN-D1b-2*, *qPN-N-1*, *qPN-C2-2*, *qPN-C2-3*, *qPN-A2-1*, *qPN-K-1*, *qPN-O-2*, *qPN-B1-2*, *qPN-B1-4 qPN-G-4* and *qPN-L-2*) were found in RIL6013, and the Henong 60 alleles for all 13 of these QTLs increased PNBD.

Three TPD QTLs (*qPN-D1a-2*, *qPN-B2-1* and *qPN-G-1*) were detected in LGs D1a, B2 andG in RIL3613. Alleles of these three QTLs from Heihe 36, increased TPD. Eight QTLs (*qPN-N-1*, *qPN-O-2*, *qPN-B1-2*, *qPN-B1-3*, *qPN-B1-4*, *qPN-G-4*, *qPN-L-3*, and *qPN-I-2*) underlying TPD were detected in RIL6013 in LGs B1, G, L, O, N, and I, and the negative additive effects of these eight QTLs indicated that the alleles from Henong 60 increased TPD.

## Discussion

### Analyzing pod numbers in three sections of the plant increased QTL detection power and could facilitate yield improvement

Most genetic and breeding research has focused on the total pod number of entire plants [2–18], ignoring the impact of the vertical distribution of pods. In this research, the pod number of the entire plant was divided into three vertical sections, which enhanced the power of QTL detection. Only 16 QTLs for TPA, TPB, TPC, and TPD were detected in the two populations; however, when the QTL analysis was conducted on the three distinct sections of the plants, 31 other QTLs for pod number were identified. Furthermore, this result elucidated the molecular basis underlying the phenotypic variation in different types of pod in the different sections of the plant, which could enable breeders to improve seed set, by combining desirable genotypes controlling seeds per pod and pod number in the various parts of the plant. Our findings highlight the importance of distinguishing the genetic regulation of seed set and pod numbers in different regions of the plant. Thus, it is necessary to map QTLs by examining the pod numbers separately in upper, middle and lower parts of the plant.

### Identification of QTL alleles for use in molecular breeding

Mapping the QTLs that regulate the spatial distribution of pods will enable the practical improvement of seed yields, as breeders can combine QTLs controlling pod numbers in different areas of the plant. The favorable allelic genotypes should be transposed from specific parents to the offspring. In this research, we identified 21 and 26 QTLs associated with pod-number-related traits in RIL3613 and RIL6013, respectively. For RIL3613, QTLs with the favorable genotypes derived from Dongnong L13 include *qPN-A1-2*, *qPN-M-1*, *qPN-O-3*, *qPN-O-3*, *PN-E-2* and *qPN-D2-1*; and those from Heihe 36 include *qPN-D1a-2*, *qPN-D1b-3*, *qPN-C1-1*, *qPN-C1-3*, *qPN-A1-1*, *qPN-C2-1*, *qPN-M-2*, *qPN-O-1*, *qPN-F-1*, *qPN-B2-1*, *qPN-D2-2*, *qPN-G-1*, *qPN-L-1*, *qPN-I-1* and *qPN-I-3*. In the RIL6013 population, the QQ alleles from Dongnong L13 proved to be favorable allelic genotypes for *qPN-H-1* and *qPN-L-4* QTL, whereas Henong 60 carried favorable allelic genotypes for the other 24 QTLs. To integrate these favorable alleles into one line, individuals carrying different combinations of favorable alleles which could be predicted by Bayesian probability, should be selected for crossing. The offspring could then be screened using marker-assisted selection.

### Verification of QTLs

The authenticity of QTLs can be verified by a comparison of the genomic regions containing QTLs in different genetic backgrounds, or by identifying a single QTL that regulates multiple related traits.

To compare genome regions containing QTLs, we integrated all genomic fragments encompassing pod-number QTLs found in the present and previous studies into the pubic linkage map of the soybean genome [22] (S1 Table). Of all the genomic regions associated with the 47 QTLs identified in the present research, 14 and 21 regions associated with pod-number-related traits in RIL3613 and RIL6013 overlapped with those identified in previous reports, respectively.

A total of 11regions contained QTLs detected in both associated RIL populations (Table 2, S1 Table). The *qPN-C1-1* region (24.11–41.43 cM; Satt396–Sat_140) overlapped the *qPN-C1-2* region (28.04–41.43 cM; sat_367–sat_140) and the *qPN-C2-1* region (107.58–112.34 cM; Satt277–Satt289) overlapped the *qPN-C2-2* region (97.83–121.26 cM; satt376–satt307). The *qPN-D1a-1* region (45.75~56.43 cM; satt482~satt254) overlapped the *qPN-D1a-2* region (53.66–55.68 cM; Sat_346–Satt515). The *qPN-D1b-3* region (102.59–112.62 cM; Sat_069–Sat_183), the *qPN-D1b-1* region (0–131.91 cM; sat_096–sat_289), and the *qPN-D1b-2* region (87.19–126.44 cM; satt546–staga002) all overlapped each other. The *qPN-G-3* region (50.52–100 cM; satt352–sat_117) overlapped the *qPN-G-1* region (62.08~68.76 cM; Sat_203~Satt503). The *qPN-I-1* region (18.5–82.77 cM; Satt571–Satt292), the *qPN-I-3* region (18.5–97.04 cM; Satt571–GMGLPSI2) and the *qPN-I-2* region (27.98–77.83 cM; satt367–satt330) all overlapped each other. The *qPN-L-1* region (33.7–78.23 cM; Satt497–Sat_099) overlapped the *qPN-L-3* region (30.83–64.66 cM; sat_195–satt448) and the *qPN-L-1* region (33.7–78.23 cM; Satt497–Sat_099) overlapped the *qPN-L-4* region (34.54~107.23 cM; satt313~satt373) too. The *qPN-O-1* region (14.17–118.13 cM; Satt500–Satt153), the *qPN-O-2* region (28.95–51 cM; BF008905–Sat_221) overlapped the *qPN-O-2* region (54.2–67.93 cM; Satt479–Sat_341). These 11 regions containing overlapping QTLs represent strong candidates for breeding programs to affect seed set and pod number.

Pleiotropic effects detected for a QTL also indicate its validity. Among the 47 QTLs detected in the present study, 23 were found to control multiple traits. In RIL3613, seven QTLs (*qPN-A1-2*, *qPN-C1-1*, *qPN-D1a-2*, *qPN-D2-1*, *qPN-D2-2*, *qPN-I-3*, and *qPN-M-1*) controlled two traits, one QTLs (*qPN-B2-1*) affected three traits. In RIL6013, five QTLs (*qPN-B1-3*, *qPN-D1a-1*, *qPN-D1b-1*, *qPN-E-3* and *qPN-G-3*) controlled two traits, four QTLs (*qPN-B1-2*, *qPN-D1b-2*, *qPN-G-2* and *qPN-G-4*) were associated with three traits, five QTLs (*qPN-B1-1*, *qPN-B1-4*, *qPN-I-2*, *qPN-L-3* and *qPN-O-2*) regulated four traits, and one QTLs (*qPN-N-1*) conferred five traits. The association of these regions with multiple traits suggest the authenticity of these 23 QTLs.

## Conclusion

A total of 21 and 26 QTLs controlling pod-number-related traits were identified in RIL3613 and RIL6013, respectively. Of all the identified QTLs, 32 QTLs were confirmed by comparison with previous research, were found to regulate multiple traits, or were identified in both associated RIL populations.

## Supporting information

S1 Fig The frequency distribution of pod-number-related traits. (DOCX)

S2 Fig Linkage map of RIL3613. (DOCX)

S3 Fig Linkage map of RIL6013. (DOCX)

S1 Table Genomic region of QTLs associated with pod number-related traits detected in present and previous research. (DOCX)

S2 Table Linkage map information for RIL3613 and RIL6013. (DOCX)

S1 Dataset Genotypic and phenotypic data. (PDF)

## Abbreviations

LG: Linkage group
PNUA: Number of pods containing one seed in the upper part of the plant
PNMA: Number of pods containing one seed in the middle plant section
PNBA: Number of pods containing one seed in the lower part of the plant
PNUB: Number of pods containing two seeds in the upper part of the plant
PNMB: Number of pods containing two seeds in the middle part of the plant section
PNBB: Number of pods containing two seeds in the lower part of the plant
PNUC: Number of pods containing three seeds in the upper part of the plant
PNMC: Number of pods containing three seeds in the middle plant section
PNBC: Number of pods containing three seeds in the lower part of the plant
PNUD: Number of pods containing four seeds in the upper part of the plant
PNMD: Number of pods containing four seeds in the middle plant section
PNBD: Number of pods containing four seeds in the lower part of the plant
TPA: Total number of pods containing one seed
TPB: Total number of pods containing two seeds
TPC: Total number of pods containing three seeds
TPD: Total number of pods containing four seeds
QTL: Quantitative trait locus
ICIM: Inclusive complete interval mapping method
