# Supplementary material for: Identification of QTLs related to the vertical distribution and seed-set of pod number in soybean [Glycine max (L.) Merri]
Source: PLoS One. 2018 Apr 17;13(4):e0195830. doi: 10.1371/journal.pone.0195830 (PMC5903612; doi:10.1371/journal.pone.0195830)
Supplement: S1 Fig — (DOCX) [file pone.0195830.s001.docx]

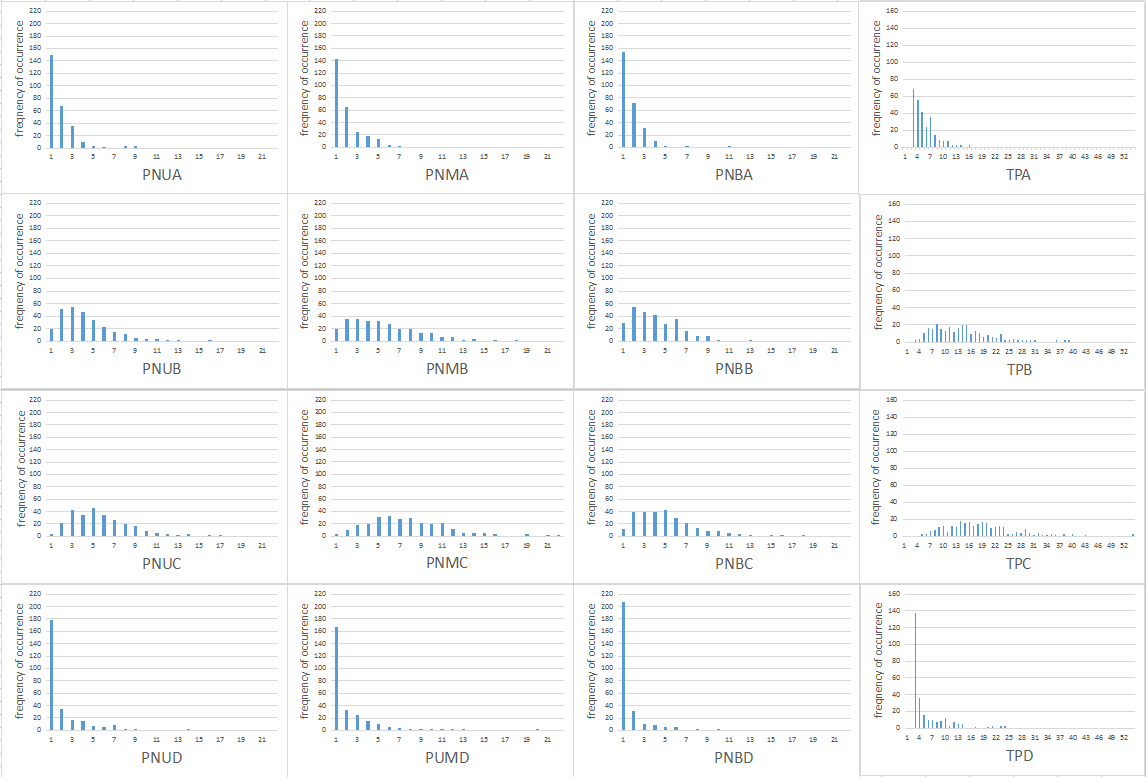


**Fig S1. Frequency distribution of pod-number-related traits**

PNUA, number of pods containing one seed in the upper part of the plant; PNMA, number of pods containing one seed in the middle plant section; PNBA, number of pods containing one seed in the lower part of the plant; PNUB, number of pods containing two seeds in the upper part of the plant; PNMB, number of pods containing two seeds in the middle plant section; PNBB, number of pods containing two seeds in the lower part of the plant; PNUC, number of pods containing three seeds in the upper part of the plant; PNMC, number of pods containing three seeds in the middle plant section; PNBC, number of pods containing three seeds in the lower part of the plant; PNUD, number of pods containing four seeds in the upper part of the plant; PNMD, number of pods containing four seeds in the middle plant section; PNBD, number of pods containing four seeds in the lower part of the plant; TPA, total number of pods containing one seed; TPB, total number of pods containing two seeds; TPC, total number of pods containing three seeds; TPD, total number of pods containing four seeds.
