# Supplementary figures and images for: Identification of QTLs related to the vertical distribution and seed-set of pod number in soybean [Glycine max (L.) Merri]
Source: PLoS One. 2018 Apr 17;13(4):e0195830. doi: 10.1371/journal.pone.0195830 (PMC5903612; doi:10.1371/journal.pone.0195830)

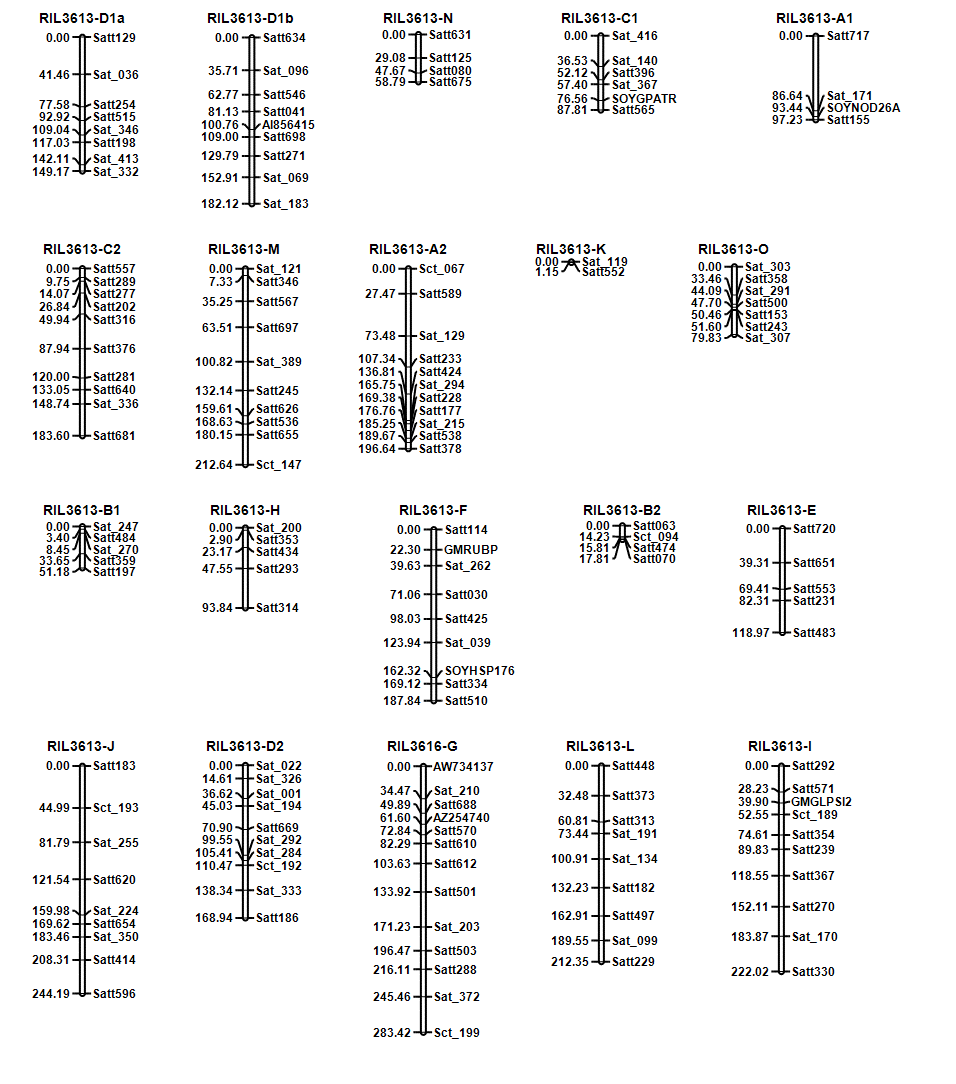


**Fig S2. Linkage map of RIL3613**

Supplement: S2 Fig — (DOCX) [file pone.0195830.s002.docx]

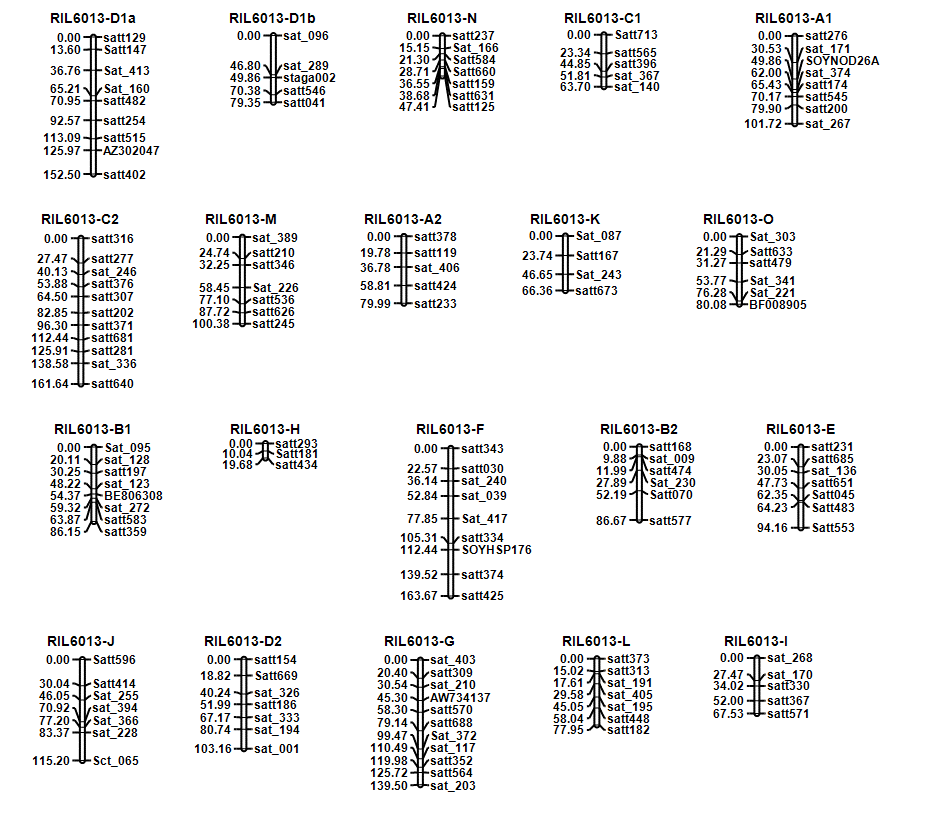


**Fig S3. Linkage map of RIL6013**

Supplement: S3 Fig — (DOCX) [file pone.0195830.s003.docx]
