# Supplementary material for: Identification of QTLs related to the vertical distribution and seed-set of pod number in soybean [Glycine max (L.) Merri]
Source: PLoS One. 2018 Apr 17;13(4):e0195830. doi: 10.1371/journal.pone.0195830 (PMC5903612; doi:10.1371/journal.pone.0195830)
Supplement: S1 Table — (DOCX) [file pone.0195830.s004.docx]

**Table S1 Genomic region of QTLs associated with pod number-related traits detected in presents and previous researches**

| QTL | Trait | LG | Method | Marker interval | Genomic Region | Population | Re-identification |
| --- | --- | --- | --- | --- | --- | --- | --- |
| *qPN-D1a-1* | PNBD | Ch01 (D1a) | ICIM/ CIM | satt482~satt254 | 45.75~56.43 cM | RIL6013 | qPN-D1a-2 in RIL3613 |
| *qPN-D1a-1* | PNUA | Ch01 (D1a) | ICIM/ CIM | satt482~satt254 | 45.75~56.43 cM | RIL6013 |  |
| *qPN-D1a-2* | PNMD | Ch01 (D1a) | ICIM/ CIM/ SMA | Sat_346~Satt198 | 53.66~68.62 cM | RIL3613 | qPN-D1a-1 in RIL6013 |
| *qPN-D1a-2* | TPD | Ch01 (D1a) | ICIM/ CIM | Sat_346~Satt198 | 53.66~68.62 cM | RIL3613 |  |
| *qPN-D1b-1* | PNUA | Ch02 (D1b) | ICIM/ CIM | sat_096~sat_289 | 0.00~131.91 cM | RIL6013 | [9][18], *qPN-D1b-3* in RIL3613 |
| *qPN-D1b-1* | PNBD | Ch02 (D1b) | ICIM/ CIM | sat_096~sat_289 | 0~131.91 cM | RIL6013 |  |
| *qPN-D1b-3* | TPA | Ch02 (D1b) | ICIM/ SMA | Sat_069~Sat_183 | 102.59~112.62 cM | RIL3613 | qPN-D1b-1 and qPN-D1b-2 in RIL6013 |
| *qPN-D1b-2* | PNMC | Ch02 (D1b) | ICIM /SMA | staga002 | 126.44 cM | RIL6013 |  |
| *qPN-D1b-2* | PNBD | Ch02 (D1b) | ICIM /CIM | satt546~staga002 | 87.19~126.44 cM | RIL6013 | *qPN-D1b-3* in RIL3613 |
| *qPN-D1b-2* | PNMA | Ch02 (D1b) | ICIM /CIM | satt546~staga002 | 87.19~126.44 cM | RIL6013 |  |
|  | TPNPP | Ch02 (D1b) |  | Sat_279~ Satt216 | 3.79~9.3 cM |  | [18] |
|  | TPNPP | Ch02 (D1b) |  | BE475343~Satt095 | 25.6~30.74 |  | [9] |
| *qPN-N-1* | PNBA | Ch03 (N) | ICIM/ CIM | Sat_166~satt237 | 38.59~74.98 cM | RIL6013 | [19] |
| *qPN-N-1* | PNBD | Ch03 (N) | ICIM/ CIM | Sat_166~satt237 | 38.59~74.98 cM | RIL6013 |  |
| *qPN-N-1* | PNMD | Ch03 (N) | ICIM/CIM/ SMA | Sat_166~satt237 | 38.59~74.98 cM | RIL6013 |  |
| *qPN-N-1* | PNUA | Ch03 (N) | ICIM/ CIM | Sat_166~satt237 | 38.59~74.98 cM | RIL6013 |  |
| *qPN-N-1* | TPD | Ch03 (N) | ICIM/ CIM | Sat_166~satt237 | 38.59~74.98 cM | RIL6013 |  |
|  | TPNPP | Ch03 (N) |  | Satt152~Satt080 | 22.67~45.13 cM |  | [19] |
| *qPN-C1-1* | PNBB | Ch04 (C1) | ICIM/ CIM/ SMA/ SMA | Satt396~Sat_140 | 24.11~41.43 cM | RIL3613 | [5][17], *qPN-C1-2* in RIL6013 |
| *qPN-C1-1* | TPA | Ch04 (C1) | CIM/ SMA/ SMA | Satt396~Sat_140 | 24.11~41.43 cM | RIL3613 |  |
| *qPN-C1-2* | PNBA | Ch04 (C1) | ICIM/ CIM/ SMA/ SMA | sat_367~sat_140 | 28.04~41.43 cM | RIL6013 | [5][17], qPN-C1-1 in RIL3613 |
| *qPN-C1-3* | PNUA | Ch04 (C1) | CIM/ SMA | Sat_140~Sat_416 | 41.43~76.41 cM | RIL3613 | [2][5][19] |
|  | TPNPP | Ch04 (C1) |  | Satt565~Satt180 | 0~127.76 |  | [5] |
|  | TPA | Ch04 (C1) |  | Soygpatr ~Sat_367 | 10.34~28.04 |  | [17] |
|  | TPB | Ch04 (C1) |  | Soygpatr ~Sat_367 | 10.34~28.04 |  | [17] |
|  | TPA | Ch04 (C1) |  | Sat_367~Satt578 | 28.04~65.08 |  | [5] |
|  | TPNPP | Ch04 (C1) |  | Satt578~Satt661 | 65.08~74.36 |  | [19] |
|  | TPNPP | Ch04 (C1) |  | Satt661~Satt294 | 74.36~78.65 |  | [19] |
|  | TPNPP | Ch04 (C1) |  | Satt139~Satt476 | 74.5~85.70 |  | [2] |
| *qPN-A1-1* | PNMA | Ch05 (A1) | ICIM/ CIM | Satt717~Sat_171 | 51.95~57.79 cM | RIL3613 |  |
| *qPN-A1-2* | PNBD | Ch05 (A1) | ICIM/ CIM | SOYNOD26A~Sat_171 | 57.24~57.79 cM | RIL3613 |  |
| *qPN-A1-2* | PNMD | Ch05 (A1) | ICIM/ CIM | SOYNOD26A~Sat_171 | 57.24~57.79 cM | RIL3613 |  |
| *qPN-C2-1* | PNMA | Ch06 (C2) | ICIM/ CIM | Satt277~Satt289 | 107.58~112.34 cM | RIL3613 | [3][6][7], qPN-C2-2 in RIL6013 |
| *qPN-C2-3* | PNBD | Ch06 (C2) | ICIM/ CIM | satt307~satt202 | 121.26~126.23 cM | RIL6013 | [20] |
| *qPN-C2-2* | PNBD | Ch06 (C2) | ICIM/ CIM/ SMA | satt376~satt307 | 97.83~121.26 cM | RIL6013 | [3][6][7][17][20][21], qPN-C2-1 in RIL3613 |
|  | TPNPP | Ch06 (C2) |  | Satt277 | 107.58 |  | [3] |
|  | TPNPP | Ch06 (C2) |  | Satt277 | 107.58 |  | [3] |
|  | TPNPP | Ch06 (C2) |  | Satt277 | 107.58 |  | [7] |
|  | TPNPP | Ch06 (C2) |  | Satt277~Satt489 | 107.58~113.38 |  | [6] |
|  | TPNPP | Ch06 (C2) |  | Satt277~Satt489 | 107.58~113.38 |  | [6] |
|  | TPNPP | Ch06 (C2) |  | Satt100 | 113.95 |  | [7] |
|  | TPNPP | Ch06 (C2) |  | Satt100 | 113.95 |  | [21] |
|  | TPNPP | Ch06 (C2) |  | Satt489~Satt557 | 113.38~112.19 |  | [6] |
|  | TPNPP | Ch06 (C2) |  | Satt489~Satt557 | 113.38~112.19 |  | [6] |
|  | TPNPP | Ch06 (C2) |  | Satt460~Satt460 | 116.76~121.76 |  | [20] |
|  | TPNPP | Ch06 (C2) |  | Satt460~Satt079 | 117.76~117.87 |  | [17] |
| *qPN-M-1* | PNBA | Ch07 (M) | ICIM/ SMA | Sat_389~Satt697 | 0.00~85.34 cM | RIL3613 | [5] |
| *qPN-M-1* | TPA | Ch07 (M) | CIM/ SMA | Sat_389~Satt697 | 0.00~85.34 cM | RIL3613 |  |
| *qPN-M-2* | PNMB | Ch07 (M) | ICIM/ CIM/ SMA/ SMA | Satt626~Satt536 | 58.59~62.13 cM | RIL3613 |  |
|  | TPC | Ch07 (M) |  | Satt540~Sat_244 | 35.84~48.86 |  | [5] |
|  | TPD | Ch07 (M) |  | Satt551~Sat_121 | 95.44~103.98 |  | [5] |
| *qPN-A2-1* | PNBD | Ch08 (A2) | ICIM/ CIM | sat_406~satt424 | 25.90~60.59 cM | RIL6013 | [5][18][19] |
|  | TPD | Ch08 (A2) |  | Sat_383~Satt480 | 0~28.44 |  | [5] |
|  | TPNPP | Ch08 (A2) |  | Sat_215~Sat_409 | 35.68~53.75 |  | [18] |
|  | TPNPP | Ch08 (A2) |  | Sct_067~Satt589 | 14.99~33.95 |  | [19] |
| *qPN-K-1* | PNBD | Ch09 (K) | ICIM/ CIM/ SMA | satt673~Sat_243 | 50.79~86.77 cM | RIL6013 | [2] |
|  | TPNPP | Ch09 (K) |  | Satt475 | 66.43~90.93 |  | [2] |
| *qPN-O-3* | PNBB | Ch10 (O) | ICIM/ SMA | Satt153~Satt243 | 118.13~119.50 cM | RIL3613 | [5][9] |
| *qPN-O-4* | PNMB | Ch10 (O) | ICIM/ CIM | Satt243~Sat_307 | 119.50~123.43 cM | RIL3613 | [5][9] |
| *qPN-O-1* | PNMD | Ch10 (O) | ICIM/ CIM | Satt500~Satt153 | 14.17~118.13 cM | RIL3613 | [5][9][19][20], *qPN-O-2* in RIL6013 |
| *qPN-O-2* | PNBD | Ch10 (O) | ICIM/ CIM | satt479~Sat_341 | 54.2~67.93 cM | RIL6013 | [19][20], *qPN-O-1* in RIL3613 |
| *qPN-O-2* | PNMD | Ch10 (O) | ICIM/ CIM | satt479~Sat_341 | 54.2~67.93 cM | RIL6013 |  |
| *qPN-O-2* | PNUA | Ch10 (O) | CIM/ SMA | satt479~Sat_341 | 54.2~67.93 cM | RIL6013 |  |
| *qPN-O-2* | TPD | Ch10 (O) | ICIM/ CIM | satt479~Sat_341 | 54.2~67.93 cM | RIL6013 |  |
|  | TPA | Ch10 (O) |  | Sat_291~Satt576 | 51.9~55.81 |  | [19] |
|  | TPNPP | Ch10 (O) |  | Satt173~Satt581 | 58.4~106.02 |  | [20] |
|  | TPNPP | Ch10 (O) |  | Satt581~Satt243 | 106.02~119.5 |  | [9] |
|  | TPA | Ch10 (O) |  | Sat_274~Sat_038 | 107.58~112.16 |  | [19] |
|  | TPC | Ch10 (O) |  | Sat_038~Satt243 | 112.16~119.5 |  | [5] |
| *qPN-B1-2* | PNMD | Ch11 (B1) | ICIM/ SMA | BE806308~sat_272 | 0.00~14.32 cM | RIL6013 |  |
| *qPN-B1-2* | PNUD | Ch11 (B1) | ICIM/ CIM/ SMA | BE806308~sat_272 | 0.00~14.32 cM | RIL6013 |  |
| *qPN-B1-2* | TPD | Ch11 (B1) | ICIM/ SMA | BE806308~sat_272 | 0.00~14.32 cM | RIL6013 |  |
| *qPN-B1-3* | PNMD | Ch11 (B1) | ICIM/ CIM | sat_272~satt583 | 14.32~84.19 cM | RIL6013 | [3][18] |
| *qPN-B1-3* | TPD | Ch11 (B1) | ICIM/ CIM | sat_272~satt583 | 14.32~84.19 cM | RIL6013 |  |
| *qPN-B1-1* | PNBA | Ch11 (B1) | ICIM/ CIM/ SMA | satt197~sat_123 | 46.38~100.87 cM | RIL6013 | [3][18] |
| *qPN-B1-1* | PNBC | Ch11 (B1) | ICIM/ CIM/ SMA | satt197~sat_123 | 46.38~100.87 cM | RIL6013 |  |
| *qPN-B1-1* | PNMD | Ch11 (B1) | ICIM/ CIM | satt197~sat_123 | 46.38~100.87 cM | RIL6013 |  |
| *qPN-B1-1* | PNUA | Ch11 (B1) | ICIM/ CIM/ SMA | satt197~sat_123 | 46.38~100.87 cM | RIL6013 |  |
| *qPN-B1-4* | PNBD | Ch11 (B1) | ICIM/ CIM/ SMA/ SMA | satt583~satt359 | 84.19~102.55 cM | RIL6013 |  |
| *qPN-B1-4* | PNMD | Ch11 (B1) | ICIM/ CIM/ SMA | satt583~satt359 | 84.19~102.55 cM | RIL6013 |  |
| *qPN-B1-4* | PNUD | Ch11 (B1) | ICIM/ CIM/ SMA | satt583~satt359 | 84.19~102.55 cM | RIL6013 |  |
| *qPN-B1-4* | TPD | Ch11 (B1) | ICIM/ CIM/ SMA/ SMA | satt583~satt359 | 84.19~102.55 cM | RIL6013 |  |
|  | TPNPP | Ch11 (B1) |  | Satt426 | 28.33~40.33 |  | [3] |
|  | TPNPP | Ch11 (B1) |  | Satt509 | 32.5~47.6 |  | [3] |
|  | TPNPP | Ch11 (B1) |  | Satt197~Satt251 | 36.48~46.38 |  | [18] |
| *qPN-H-1* | PNBA | Ch12 (H) | ICIM/ CIM | satt293~Satt181 | 89.08~91.12 cM | RIL6013 |  |
| *qPN-F-1* | PNMA | Ch13 (F ) | ICIM/ CIM | GMRUBP~Sat_262 | 0.00~9.69 cM | RIL3613 |  |
| *qPN-B2-1* | PNBD | Ch14(B2 ) | ICIM/ CIM | Sct_094~Satt063 | 70.55~93.48 cM | RIL3613 |  |
| *qPN-B2-1* | PNMD | Ch14(B2 ) | ICIM/ CIM | Sct_094~Satt063 | 70.55~93.48 cM | RIL3613 |  |
| *qPN-B2-1* | TPD | Ch14(B2 ) | ICIM/ CIM | Sct_094~Satt063 | 70.55~93.48 cM | RIL3613 |  |
| *qPN-E-3* | PNBA | Ch15(E) | ICIM/ CIM | sat_136~satt651 | 32.09~39.16 cM | RIL6013 |  |
| *qPN-E-3* | PNUD | Ch15(E) | ICIM/ CIM | sat_136~satt651 | 32.09~39.16 cM | RIL6013 |  |
| *qPN-E-1* | PNMA | Ch15(E) | ICIM/ CIM | satt685~satt231 | 56.70~70.23 cM | RIL6013 | [18] |
| *qPN-E-2* | PNMD | Ch15(E) | CIM/ SMA/ SMA | Satt553~Satt231 | 67.91~70.23 cM | RIL3613 |  |
|  | TPNPP | Ch15(E) |  | Sat_381~Satt231 | 64.18~70.23 |  | [18] |
| *qPN-J-1* | PNBC | Ch16(J ) | ICIM/ CIM | sat_228~Sat_366 | 23.91~52.84 cM | RIL6013 | [18] |
| *qPN-J-2* | PNUA | Ch16(J ) | ICIM/ CIM | Sat_366~sat_394 | 52.84~89.43 cM | RIL6013 |  |
|  | TPNPP | Ch15(E) |  | Satt596~Satt622 | 39.63~42.25 |  | [18] |
| *qPN-D2-2* | PNBD | Ch17( D2 ) | ICIM/ CIM | Sct_192~Sat_284 | 11.77~30.79 cM | RIL3613 | [17] |
| *qPN-D2-2* | PNMA | Ch17( D2 ) | ICIM/ CIM | Sct_192~Sat_284 | 11.77~30.79 cM | RIL3613 |  |
| *qPN-D2-1* | PNBD | Ch17( D2 ) | ICIM/ CIM | Sat_333~Sct_192 | 5.83~11.77 cM | RIL3613 |  |
| *qPN-D2-1* | PNMC | Ch17( D2 ) | CIM/ SMA | Sat_333~Sct_192 | 5.83~11.77 cM | RIL3613 |  |
|  | TPNPP | Ch17( D2 ) |  | Satt256~Satt458 | 24.52~124.3 |  | [17] |
| *qPN-G-2* | PNMB | Ch18(G ) | CIM/ SMA | sat_210~satt309 | 3.7~4.53 cM | RIL6013 |  |
| *qPN-G-2* | TPB | Ch18(G ) | CIM/ SMA | sat_210~satt309 | 3.7~4.53 cM | RIL6013 |  |
| *qPN-G-2* | TPC | Ch18(G ) | CIM/ SMA | sat_210~satt309 | 3.7~4.53 cM | RIL6013 |  |
| *qPN-G-3* | PNUA | Ch18(G ) | ICIM/ CIM | satt352~sat_117 | 50.52~100.00 cM | RIL6013 | [6][18], qPN-G-1 in RIL3613 |
| *qPN-G-3* | PNUD | Ch18(G ) | ICIM/ CIM | satt352~sat_117 | 50.52~100.00 cM | RIL6013 |  |
| *qPN-G-4* | PNBD | Ch18(G ) | ICIM/ CIM/ SMA | satt352~satt564 | 50.52~57.32 cM | RIL6013 | [18] |
| *qPN-G-4* | PNMD | Ch18(G ) | ICIM/ CIM/ SMA | satt352~satt564 | 50.52~57.32 cM | RIL6013 |  |
| *qPN-G-4* | TPD | Ch18(G ) | ICIM/ CIM/ SMA | satt352~satt564 | 50.52~57.32 cM | RIL6013 |  |
| *qPN-G-1* | TPD | Ch18(G ) | CIM/ SMA | Sat_203~Satt503 | 62.08~68.76 cM | RIL3613 | qPN-G-3 in RIL6013 |
|  | TPNPP | Ch18(G ) |  | Satt138~Satt564 | 55.99~57.32 |  | [18] |
|  | TPB | Ch18(G ) |  | AF162283~Satt472 | 87.94~94.83 |  | [6] |
| *qPN-L-2* | PNBD | Ch19 (L ) | ICIM/ CIM | sat_405~sat_195 | 29.62~30.83 cM | RIL6013 | [18] |
| *qPN-L-3* | PNBC | Ch19 (L ) | ICIM/ CIM | sat_195~satt448 | 30.83~64.66 cM | RIL6013 | [9][18], qPN-*L*-1 in RIL3613 |
| *qPN-L-3* | PNMD | Ch19 (L ) | ICIM/ CIM | sat_195~satt448 | 30.83~64.66 cM | RIL6013 |  |
| *qPN-L-3* | PNUD | Ch19 (L ) | ICIM/ CIM | sat_195~satt448 | 30.83~64.66 cM | RIL6013 |  |
| *qPN-L-3* | TPD | Ch19 (L ) | ICIM/ CIM | sat_195~satt448 | 30.83~64.66 cM | RIL6013 |  |
| *qPN-L-1* | PNMA | Ch19 (L ) | ICIM/ CIM | Satt497~Sat_099 | 33.70~78.23 cM | RIL3613 | [9][18], *qPN-L-3* and *qPN-L-4* in RIL6013 |
| *qPN-L-4* | PNMC | Ch19 (L ) | CIM/ SMA | satt313~satt373 | 34.54~107.23 cM | RIL6013 | [5][8][9][18],qPN-*L*-1 in RIL3613 |
|  | TPNPP | Ch19 (L ) |  | Satt373~Satt495 | 0~107.23 |  | [18] |
|  | TPD | Ch19 (L ) |  | Satt182~Satt388 | 14.03~23.54 |  | [5] |
|  | TPNPP | Ch19 (L ) |  | Satt076~Satt561 | 61.34~71.44 |  | [9] |
|  | TPNPP | Ch19 (L ) |  | Sat_099~Satt229 | 78.23~93.88 |  | [8] |
|  | TPNPP | Ch19 (L ) |  | Sat_286~Satt664 | 87.41~92.66 |  | [9] |
| *qPN-I-2* | PNMA | Ch20 (I ) | ICIM/ CIM | satt571~satt367 | 18.50~27.98 cM | RIL6013 | qPN-I-1 and *qPN-I-3* in RIL3613 |
| *qPN-I-2* | PNMD | Ch20 (I ) | ICIM/ CIM | satt571~satt367 | 18.50~27.98 cM | RIL6013 |  |
| *qPN-I-2* | PNUD | Ch20 (I ) | ICIM/ CIM | satt571~satt367 | 18.50~27.98 cM | RIL6013 |  |
| *qPN-I-2* | TPD | Ch20 (I ) | ICIM/ CIM | satt571~satt367 | 18.50~27.98 cM | RIL6013 |  |
| *qPN-I-1* | PNUA | Ch20 (I ) | ICIM/ CIM/ SMA | Satt571~Satt292 | 18.50~82.77 cM | RIL3613 | [6][17][19], *qPN-I-2* in RIL6013 |
| *qPN-I-3* | PNBA | Ch20 (I ) | ICIM/ CIM/ SMA | Satt571~GMGLPSI2 | 18.50~97.04 cM | RIL3613 | [6][17][19], *qPN-I-2* in RIL6013 |
| *qPN-I-3* | TPA | Ch20 (I ) | ICIM/ SMA | Satt571~GMGLPSI2 | 18.50~97.04 cM | RIL3613 |  |
|  | TPNPP | Ch20 (I ) |  | Satt292~Satt354 | 46.22~82.77 cM |  | [17] |
|  | NSPP | Ch20 (I ) |  | Sat_105~Sat_268 | 49.34~55.09 cM |  | [25] |
|  | TPB | Ch20 (I ) |  | Sat_268~Satt671 | 55.09~72.08 cM |  | [6] |
|  | TPC | Ch20 (I ) |  | Sat_268~Satt671 | 55.09~72.08 cM |  | [6] |
|  | TPD | Ch20 (I ) |  | Sat_268~Satt671 | 55.09~72.08 cM |  | [6] |
|  | TPD | Ch20 (I ) |  | Sat_268~Satt671 | 55.09~72.08 cM |  | [19] |
|  | TPA | Ch20 (I ) |  | Sat_268~Satt671 | 55.09~72.08 cM |  | [19] |
|  | TPNPP | Ch20 (I ) |  | Sat_268~Satt671 | 55.09~72.08 cM |  | [19] |
|  | TPNPP | Ch20 (I ) |  | Sat_418~Satt330 | 74.26~77.83 cM |  | [6] |

A: Items with bold typeface were detected of present research.

B: PNUA, number of pods containing one seed in the upper plant; PNMA, number of pods containing one seed in the middle plant section; PNBA, number of pods containing one seed in the lower plant; PNUB, number of pods containing two seeds in the upper plant; PNMB, number of pods containing two seeds in the middle plant section; PNBB, number of pods containing two seeds in the lower plant; PNUC, number of pods containing three seeds in the upper plant; PNMC, number of pods containing three seeds in the middle plant section; PNBC, number of pods containing three seeds in the lower plant; PNUD, number of pods containing four seeds in the upper plant; PNMD, number of pods containing four seeds in the middle plant section; PNBD, number of pods containing four seeds in the lower plant; TPA, total number of pods containing one seed; TPB, total number of pods containing two seeds; TPC, total number of pods containing three seeds; TPD, total number of pods containing four seeds; NSPP , number of seed per pod; TPNPP, Total pod number per plant.

C: ICIM, inclusive compositive interval mapping method () ; CIM, compositive interval mapping method; SMA, single marker analysis method.
