# Supplementary material for: Identification of QTLs related to the vertical distribution and seed-set of pod number in soybean [Glycine max (L.) Merri]
Source: PLoS One. 2018 Apr 17;13(4):e0195830. doi: 10.1371/journal.pone.0195830 (PMC5903612; doi:10.1371/journal.pone.0195830)
Supplement: S2 Table — (DOCX) [file pone.0195830.s005.docx]

**Table S2 Linkage map information for RIL3613 and RIL6013**

| Linkage group | RIL3613 | | RIL6013 | |
| --- | --- | --- | --- | --- |
|  | No. of markers | Length (cM) | No. of markers | Length (cM) |
| D1a | 8 | 149.17 | 9 | 152.50 |
| D1b | 9 | 182.12 | 5 | 79.35 |
| N | 4 | 58.79 | 7 | 47.41 |
| C1 | 6 | 87.81 | 5 | 63.70 |
| A1 | 4 | 97.23 | 8 | 101.72 |
| C2 | 10 | 183.60 | 11 | 161.64 |
| M | 10 | 212.64 | 7 | 100.38 |
| A2 | 11 | 196.64 | 5 | 79.99 |
| K | 2 | 1.15 | 4 | 66.36 |
| O | 7 | 79.83 | 6 | 80.08 |
| B1 | 5 | 51.18 | 8 | 86.15 |
| H | 5 | 93.84 | 3 | 19.68 |
| F | 9 | 187.84 | 9 | 163.67 |
| B2 | 4 | 17.81 | 6 | 86.67 |
| E | 5 | 118.97 | 7 | 94.16 |
| J | 9 | 244.19 | 7 | 115.20 |
| D2 | 10 | 168.94 | 7 | 103.16 |
| G | 13 | 283.42 | 11 | 139.50 |
| L | 9 | 212.35 | 7 | 77.95 |
| I | 10 | 222.02 | 5 | 67.53 |
| Total | 150 | 2849.54 | 137 | 1886.8 |
