# Supplementary material for: Identification of QTLs related to the vertical distribution and seed-set of pod number in soybean [Glycine max (L.) Merri]
Source: PLoS One. 2018 Apr 17;13(4):e0195830. doi: 10.1371/journal.pone.0195830 (PMC5903612; doi:10.1371/journal.pone.0195830)
Supplement: S1 Dataset — (PDF) [file pone.0195830.s006.pdf]

Genotype and phenotypic data for RIL3613

Linkage group

Satt129	1	0
Sat_036	1	41.46
Satt254	1	77.58
Satt515	1	92.92
Sat_346	1	109.04
Satt198	1	117.03
Sat_413	1	142.11
Sat_332	1	149.17
Satt634	2	0
Sat_096	2	35.71
Satt546	2	62.77
Satt041	2	81.13
AI856415	2	100.76
Satt698	2	109
Satt271	2	129.79
Sat_069	2	152.91
Sat_183	2	182.12
Satt631	3	0
Satt125	3	29.08
Satt080	3	47.67
Satt675	3	58.79
Sat_416	4	0
Sat_140	4	36.53
Satt396	4	52.12
Sat_367	4	57.4
SOYGPATR	4	76.56
Satt565	4	87.81
Satt717	5	0
Sat_171	5	86.64
SOYNOD26A	5	93.44
Satt155	5	97.23
Satt557	6	0
Satt289	6	9.75
Satt277	6	14.07
Satt202	6	26.84
Satt316	6	49.94
Satt376	6	87.94
Satt281	6	120
Satt640	6	133.05
Sat_336	6	148.74
Satt681	6	183.6

Sat_121	7	0
Satt346	7	7.33
Satt567	7	35.25
Satt697	7	63.51
Sat_389	7	100.82
Satt245	7	132.14
Satt626	7	159.61
Satt536	7	168.63
Satt655	7	180.15
Sct_147	7	212.64
Sct_067	8	0
Satt589	8	27.47
Sat_129	8	73.48
Satt233	8	107.34
Satt424	8	136.81
Sat_294	8	165.75
Satt228	8	169.38
Satt177	8	176.76
Sat_215	8	185.25
Satt538	8	189.67
Satt378	8	196.64
Sat_119	9	0
Satt552	9	1.15
Sat_303	10	0
Satt358	10	33.46
Sat_291	10	44.09
Satt500	10	47.7
Satt153	10	50.46
Satt243	10	51.6
Sat_307	10	79.83
Sat_247	11	0
Satt484	11	3.4
Sat_270	11	8.45
Satt359	11	33.65
Satt197	11	51.18
Sat_200	12	0
Satt353	12	2.9
Satt434	12	23.17
Satt293	12	47.55
Satt314	12	93.84
Satt114	13	0
GMRUBP	13	22.3
Sat_262	13	39.63
Satt030	13	71.06

Satt425	13	98.03
Sat_039	13	123.94
SOYHSP176	13	162.32
Satt334	13	169.12
Satt510	13	187.84
Satt063	14	0
Sct_094	14	14.23
Satt474	14	15.81
Satt070	14	17.81
Satt720	15	0
Satt651	15	39.31
Satt553	15	69.41
Satt231	15	82.31
Satt483	15	118.97
Satt183	16	0
Sct_193	16	44.99
Sat_255	16	81.79
Satt620	16	121.54
Sat_224	16	159.98
Satt654	16	169.62
Sat_350	16	183.46
Satt414	16	208.31
Satt596	16	244.19
Sat_022	17	0
Sat_326	17	14.61
Sat_001	17	36.62
Sat_194	17	45.03
Satt669	17	70.9
Sat_292	17	99.55
Sat_284	17	105.41
Sct_192	17	110.47
Sat_333	17	138.34
Satt186	17	168.94
AW734137	18	0
Sat_210	18	34.47
Satt688	18	49.89
AZ254740	18	61.6
Satt570	18	72.84
Satt610	18	82.29
Satt612	18	103.63
Satt501	18	133.92
Sat_203	18	171.23
Satt503	18	196.47
Satt288	18	216.11

Sat_372	18	245.46
Sct_199	18	283.42
Satt448	19	0
Satt373	19	32.48
Satt313	19	60.81
Sat_191	19	73.44
Sat_134	19	100.91
Satt182	19	132.23
Satt497	19	162.91
Sat_099	19	189.55
Satt229	19	212.35
Satt292	20	0
Satt571	20	28.23
GMGLPSI2	20	39.9
Sct_189	20	52.55
Satt354	20	74.61
Satt239	20	89.83
Satt367	20	118.55
Satt270	20	152.11
Sat_170	20	183.87
Satt330	20	222.02

Marker Types

Satt129 B A A A B A B A B A A A B B A A B A
B A B A A B B B A B A B A A A B B A A
A A B A B A B B B B A A A A A B A A A
A B A B A B B A B A A B A B B B B A B
A A B A A B A B A A A B A A B A A A B
B B B A A X A B A B X X B A B A A A B
B A A B A B A A A B A B B A A A A B A
B B
Sat_036 A A A A A A B B A A B A A B A B A B
A B A B A B B B A B A B A A B B A A B
B B B X B A A A A B A A A A A B B X A
A B B B B A X A A B B B A B X B B A A
A A B A A A A A B B X B B A A A A B B
A A B B B A A A A A B B B A A A A A A
B A A B B A B A A A A B B A A A B B A
A A
Satt254 A A A B B A B B A A A A A B B B A B
A A B A A A B A B B B A A A B A A A A
A B A B A A A X X X X X A B A A A A A
B A B B A A B A A A B A A A B B A A A

A A A A X B A A A A B A A A A B A A B
A A A A A A A A A B B A B A A A A A A
B A A A A A A A A A A A B A A B A A A
A A
Satt515 A A B B A X X X A X A A A A B A X A
X X A B A A A B A X B A X A A B A A A
A B A B A X A B A X X B A A A X A A A
B A A A A A B A A A B A X X B B A A A
A A A A A A A A X A A X A A A A X B B
A A A A A B A A B A B A A A A A A A A
B A A A X A A A A A A X A X B B A A A
A A
Sat_346 A X A A X A A X A A A A A X A A B X
X A A X A A A A A A A A A X X X A A A
A B A A A A A A A A A A A A A A B A A
A A A A X A A A A B A A A A A B A A X
A A A A A B B A A A A A A A A A A A A
A A A A A A A A B A B A X X A A A A A
B A A A A A A A A B A A B A A A A A A
A B
Satt198 B B A A A A A A A A A A A A A A A A
A A A A A A A A A A A A A A A A A A A
A A A A A A A A A A A A A A A A B A A
A A A A A A A A B A A A A A B A A A A
A A B A A A A A A A A A A A X A A A A
A A A A A X X A A A A A A A A A A A A
A A A A A A A A A A A A A A A A A A A
A A
Sat_413 B A B A B B A B B A A A A B B A A B
A B A B A B A A A A A A B A A B B B A
A A A A B A B A A A A A A A A A A A A
A X A A A A B B A A A A B B B B A A A
B A B B A X A B B B A A A A A A X B A
X B A A X B X A A X B B X A A A B A A
B A A A A A A A A A A B B X X A A X X
A A
Sat_332 B A B A B B A B B A A A A B B A A B
A B A B A B A A A A A A B A A B B A A
A A A A B A B A A A A A A A A A A B B
B A B A A B B B B A A A A B B B A B A
B B A B B B B B B B A A A A B A B B A
B B A A A B B A A B B B B A A A B A A
B A A A A A A A A A A B B A A B A A A
A A

Satt634 B B B B B B B B B A B B A A A A B A

B A A B A A A A A A A A B A A A B A A

A A B A A A B A A B A A A B A A B A A

A B A B B B A B A B A A B B B B A A B

B A B B A A A B B A B A A A B A A A B

B A A A A A A A A A B A B A A A B A A

B B A A B A B A A B A A B A A A B A B

A B

Sat_096 A B X B A X B A B A A A A A B A B A

B A A A A A B B A X A B A A A B B B A

B A A A B A X B B A A A A A A B B X A

B A B B A B B B A B A B X X A A B B B

B A B B B A A A B B A A A A B A A A B

X A A A X B A A A X B B B B A A A X X

A B A A B A A A A B A X A A B B A A B

A A

Satt546 A A A A A A B A B A A A A A B A B A

B B A A A A A A A A A A B A B A B B B

B A B A A A B A A B A A A A A A B A A

A B A B B B A B A B A A B B A A A B B

B A A B B B A A A B A B A A B A B B A

B A A A A A A A B X A A A B A A A A A

A B A A B B A A A B A B A A A A A A A

B A

Satt041 A A B A A B B A A A A A A A A A A A

B A A A A A A B A B A A A A B A A A B

B A A B X A B B A B B B A A A A A X A

X B A B B A A A A A A A B B B B A A B

A A B B X B A A B X A B A A B A X A A

B A A A A A B A A B A A A B A A X A A

X A A A A B A A A X A B A A A X A A A

A A

AI856415 A A A A X A X A A A A A A A A A A A

B A A B A A A A A A A A A A A A A A A

A A B A A A A A A B A A A A A A A X A

A A A B A A A A A A B A A A A X X X B

A A A A A A A A X A B B A B A A A A A

A A A A A A A A A B A A B A A A A A A

B A X A A A X A X B A X A A A X A A A

A A

Satt698 B A A A A A A A A A A A A A A A A A

A A A A A A A A A A A A A A A A A A A

A A A A A A A A A A A A A A A A B A A

A A A A A A A A B A A A A A A A A A A

A A A A A B X A A A A A A A A A A B A

A A A A A A A A A B A A A A A A A A A

B A A A A A A A A A A A A A A A A A A

A A

Satt271 A A B A B B A A B A A A B A B A A A

A B A A A A A A A A B A A A B A B B B

B A A A A A B B B B A X A A A X A A B

A X X B A B A B A A A A A B B B B B A

A A A A X X A B A A A A A A X A B B X

A A A A A B A A A X A A A A A A A A A

B A A A A A A A A A A B A A A A A A A

B B

Sat_069 A A B A B B A B B A A A B B B A B A

A B A B B A B A B B B A B A B B B B B

B A A X A A A A B A A A B B A A A A B

B B A A B B B B A A B X A A A X A B A

B A B B A B A A A A B A A A A A B B A

X A A A A A X X B A B B X A X A B A A

B A A A A A X A A A A B B A A B A A A

B B

Sat_183 X X A B A B B A B B B B A A B B B B

B A B A B B B B B A A B A B A A A A A

A B B B B A X X X A B A A B B B X A B

A A B A A A A A A A B B A B B B A B A

B A A B B B A A B A B B B B B B A A A

A A B B A B B B B B B A A B A B A A A

B B B B B B B B B A B A A B B B B A A

A A

Satt631 B A A A A B A A A B A B A A B B B B

B B B A B A A B B B A A B B A B A B A

A A A A B B A B B B B B A A B A B A B

A A B A A A B A B B A A A A A A A B B

A B B B A X B B B B A A B B X B B B B

B B A B B A X A B B B B X A B A A A X

A B A B B A A A B B A B B A A B A B B

B B

Satt125 A B B B B B A B B A B A A B A A A A

B B A B A A B B A B A A A A B A B A B

B B B B A A B A B B A A A B A B A B A

A B A B B B A B A X A B B B A A B A B

B A A B B A B A A A B A A A B A A A B

B B B B A B A A B A B A A B B A A A B

A B B B A A B B B B A B A A A A B B A

B A

Satt080	B	A	X	A	A	A	B	A	A	B	A	B	B	B	B	B	B	A
A	B	B	B	B	A	A	A	B	A	B	A	B	B	A	B	A	B	A
A	A	X	B	A	A	B	B	B	X	B	B	B	A	B	A	B	B	A
B	A	B	B	A	A	B	A	B	B	A	A	A	A	B	B	A	B	A
A	B	B	A	X	X	X	B	B	B	A	B	B	B	B	B	A	A	B
A	X	B	A	A	B	X	A	B	B	B	B	B	A	A	B	B	B	A
B	A	B	A	A	X	B	B	X	X	B	A	B	B	B	A	A	A	A
A	A																	
Satt675	B	A	A	A	A	A	A	A	A	B	A	B	B	A	B	B	B	B
A	A	B	B	B	A	A	A	B	A	B	A	A	B	A	B	A	B	A
X	A	B	X	B	B	A	B	B	B	A	B	B	A	B	A	A	B	B
B	A	B	A	A	A	B	A	B	A	B	A	X	A	A	X	A	B	A
X	B	B	B	B	B	B	B	B	B	A	B	B	B	B	B	B	B	B
B	B	B	A	A	B	X	A	B	B	B	B	X	A	A	B	B	B	A
B	A	B	A	A	A	B	B	B	A	B	A	B	B	B	B	A	A	A
B	A																	
Sat_416	A	A	B	X	A	B	B	A	A	A	B	A	A	A	A	B	A	A
A	B	A	B	A	B	B	B	A	B	A	A	B	B	B	A	A	B	A
A	B	A	B	A	B	X	A	A	B	A	A	A	A	B	A	A	B	B
A	B	A	B	A	A	A	B	B	B	A	A	A	B	A	B	A	B	B
A	A	B	B	A	B	B	A	B	A	B	A	B	B	B	B	B	B	B
B	A	A	A	A	A	B	A	B	A	A	B	A	B	B	A	B	A	B
X	B	B	A	A	B	A	B	B	B	A	B	A	A	A	A	B	X	X
A	A																	
Sat_140	A	A	B	A	A	B	A	A	A	A	A	A	B	A	B	A	A	A
A	A	A	B	A	A	A	B	A	B	B	A	B	A	A	A	A	A	A
A	B	B	B	A	A	B	A	B	B	A	B	B	B	A	A	A	X	A
B	A	A	A	A	A	A	A	A	A	B	A	B	B	X	B	A	A	A
A	A	B	B	A	A	A	B	B	B	B	A	A	A	B	A	A	A	B
B	A	A	A	A	A	B	A	X	A	A	B	A	B	A	A	B	A	A
B	X	A	A	B	A	A	A	A	A	A	B	A	A	A	A	A	A	A
A	A																	
Satt396	B	A	A	B	B	A	A	A	A	A	A	A	B	A	B	A	B	A
A	A	A	A	A	B	A	A	A	A	B	A	B	A	A	A	A	A	A
A	B	A	B	A	A	A	A	A	B	A	B	B	B	A	A	B	X	A
A	A	A	A	A	A	A	A	X	B	A	A	A	A	B	B	A	A	A
A	A	B	A	A	B	A	A	B	A	B	A	A	A	B	A	A	A	B
A	A	A	A	A	A	A	A	B	A	X	B	A	A	A	A	X	A	A
A	A	A	A	A	X	A	A	A	A	A	A	A	A	A	A	X	A	A
A	A																	
Sat_367	B	B	B	B	B	A	A	A	A	A	A	A	B	A	B	A	A	A
A	A	A	A	A	B	A	A	A	A	B	A	B	A	A	A	A	A	A
A	B	B	B	A	A	A	A	A	B	A	B	B	B	A	A	B	X	A
A	A	A	A	X	A	A	A	A	B	A	A	A	A	B	B	A	A	A

A	A	B	A	A	B	A	B	B	B	B	A	A	A	B	A	A	A	B
A	A	A	A	A	A	A	A	A	A	B	B	B	A	A	B	B	A	A
B	A	A	A	X	B	B	A	B	A	A	A	A	A	A	A	A	A	A
A	A																	
SOYGPATR	B	B	B	B	B	A	A	B	A	A	A	A	A	A	A	A	A	A
A	A	A	A	A	B	A	A	A	A	A	A	A	A	B	A	A	A	A
A	A	B	A	B	A	B	A	A	B	A	A	A	X	A	A	B	X	B
B	B	A	A	A	A	A	A	B	A	A	A	A	B	B	B	A	A	A
A	A	B	B	A	B	A	B	A	A	B	B	A	A	B	A	X	A	B
B	A	A	A	A	X	B	X	A	B	A	B	A	A	A	A	B	A	A
B	A	A	A	B	B	A	B	B	B	A	B	A	A	A	A	A	A	A
B	B																	
Satt565	B	A	B	B	B	A	B	A	A	A	A	A	A	A	A	A	A	A
A	A	A	A	A	B	A	A	A	A	A	A	A	A	A	B	A	A	B
B	B	A	B	B	A	B	A	A	B	A	A	A	B	A	A	B	B	A
B	B	A	A	A	A	A	A	A	B	A	A	A	B	B	A	A	A	A
A	A	A	B	A	A	B	B	A	A	B	A	A	A	A	A	A	A	A
B	A	A	A	A	A	B	A	A	B	A	A	B	A	A	A	B	A	A
B	A	A	A	B	B	A	A	X	X	A	B	A	A	A	A	A	A	A
B	B																	
Satt717	B	B	A	X	A	A	B	A	B	B	A	B	B	A	A	B	B	A
A	B	A	B	B	A	A	B	B	B	B	A	B	B	B	A	A	A	A
A	X	A	A	B	A	A	A	A	A	A	B	B	B	B	B	B	A	B
A	B	A	A	X	A	B	B	A	B	B	B	A	B	B	A	A	A	B
A	B	A	A	B	A	B	B	B	A	B	B	A	B	B	A	A	B	B
B	B	B	B	X	A	A	A	A	A	A	A	B	B	A	A	A	A	B
B	B	B	A	A	A	B	A	B	B	B	A	B	B	B	B	B	A	A
B	B																	
Sat_171	B	A	A	A	B	A	A	A	A	A	A	A	A	B	B	A	X	B
B	A	A	A	B	A	A	A	A	A	A	A	A	A	A	A	A	A	A
A	A	A	A	A	A	B	A	A	A	A	A	A	A	A	A	A	B	A
A	A	A	A	A	A	A	A	A	B	A	A	A	B	A	A	A	A	A
A	A	A	A	B	A	B	B	A	A	A	A	A	A	A	A	A	A	B
A	A	A	A	A	A	A	A	A	B	A	A	A	A	A	A	A	A	B
A	A	A	A	A	A	B	X	A	A	A	B	A	A	A	A	A	A	A
A	A																	
SOYNOD26A	B	B	B	B	B	B	B	B	B	B	B	B	B	B	B	B	B	B
B	B	B	B	B	B	B	B	B	B	B	B	B	B	B	B	B	B	B
B	B	A	B	B	B	B	B	B	B	B	B	B	B	B	X	B	B	B
B	B	B	B	B	B	B	X	B	B	B	B	B	B	B	B	B	B	B
B	B	B	B	B	B	X	X	B	X	B	B	B	B	B	B	B	B	A
B	B	B	B	B	B	B	B	B	B	B	B	B	B	B	B	B	B	A
B	B	B	B	B	B	X	B	B	B	B	B	B	B	B	B	B	B	B
B	B																	

Satt155 A A A A A A A A A A A A A A A A A A

A A A A A A A B A A A A A A A A A A A

A A A A A A A A A A A A A A A B A A A

A A A A A A A A B A A A A A A A A A A

A A A A A B A B A B A A A A A A A A A

A A A B B A A A A A A A A A A A A A A

B A A A A A B A A A A A A A A A A A A

A A

Satt557 A A A A A A A A A A A A A A A A B A

A A A A A A A A A A A A A X B B A A B

B B A B B B A B A B B A A A A X B X A

A A A A A A A A X B A A A A A A A A A

A A A A B B A A A A B B B B A A B A A

A A A A A A A A A B A A A A A A A A A

B A A A A A B A A A A A A B A A A A A

A A

Satt289 A A A A A A A A A A A A A A A A B A

A A A A A A A A A A A A A A A A A A A

A A A A A A A B A A A A A A A A B A A

A A A B A A A A A A A A A A A A A A A

A A A A A A A A A A A A A A A A A A A

A A A A A A A A A B A A A A A A A A A

A A A A A A B A A A A A A A A A A A A

A A

Satt277 A X A A X A A X X A A A A A A A B X

X A A X A A A A A A B X X A A X A A X

X A A A A A A A A X A A A X A X B A A

A X X A X X X X X X A A A A A A A A X

X A A A A B A A A A A A A A A A X B A

A A A A A A A A X B X A A X A A A A A

B X A A X A A A A A A A X A A X A A A

A B

Satt202 B B A A B B A A A A A A A A B A B B

B A A B A B A A A A A A B A A B A A B

B A B A B A A A A A A B A A A A A B A

B B B A A A B A X B A B A A A A A A B

A A A B A B A A A A A X A A X A B B B

B X A A A A A A B A B A X B A A A A A

B B A A B B A A A X A A B A B B A A X

B B

Satt316 B A A B B B A A B B B B A A B A B A

X B A B B A A A A A A A B B A B A A B

B A B A B B A B B B B B A A A B A B A

B B B A A A A A A B B A A A A A A A A

A A A B A B A A B A B B B A A A A A A
A B B B B B A B B B B A B B A B A B B
B B A A B B A A A B A A B A A B A A B
B B
Satt376 A B B A B B A B B A A A A B A A B B
B A A B A A A A A A A A B A B B B A B
B A B A A X B A B A A A A A A B B X A
A B B A B B B B B B B A B B B B A A B
B A A B B B A A B A A B B A B A B B X
B B B A B B B X B B B B B B A A A A B
B A A A B B A B X B A B B A B B A A B
B B
Satt281 A B X B B B A A B A A A B A B X B B
A X A B A B B B A B B B A B A B A B B
B B A B B A B A B B X A A A A A B X X
B B B A A B B B X B B A B B B B A A A
B A B B A A A A B A A A A A A A A A B
B A A A A A B A A X B B B B A A B A A
B A A A B X X A B B A B A A X B A A B
B B
Satt640 A A B B B B A A B A A A B A B A B B
A B A B A B A B A B B A A B B A B A B
B B B B B A B A A B A A B A A A A B B
B B A A A B A B B A A A A B A B A A A
B A B B A A B A B B A A A A A A A A B
B A A A B B B A A B A B B B A A B A A
B A A A A B B A A B A B A A A A A A B
B B
Sat_336 A A A B B B B A A A A A B A A A A B
A A A B A B A B A B B A A A B B A A A
B B B B B A B A B A A B B A A B B B B
B B A A A A A A A B A A B B A B A A A
A A B B A B B A B B A B A X B A B B B
B B A A A A B A B B A B B A A A B A A
B A A A A B B A A B A B A A A A A A B
B B
Satt681 B A B B A B A A B A A A B B A A A A
A A A A A B A B A A B A A A A A B B A
A A B A A A B A A A A A B A A X A X A
B B A A A B A B A B A A A B A A A B A
B A B A B A B A B X A X A A B A A A A
A X A A B A A A B A X B A A A B X A A
B A A B A A A X A A A B A B B A A A A
B A

Sat_121 B A B A B B A A A A B A B B B A A B
A B B B A A B A A A B B A A B A A B A
B A B A B A B A A A A B B A A B B B B
B A A A A A A A A B B B B B B B B B A
A A A B B A A A A A A A A A B A B B B
A A A B B A A A A A A B A B B A A A B
B A A B B B A A A A A B A A A A B B B
A A
Satt346 B B B A B B A B A A B A B A B A B A
A B A B A A B A A A B B A A B A A B A
B A X X A A B A A A A A B A A B B A B
A B A A A A A A A B B B X X X B B B A
A A A B X X B A X A A A A A X A B B B
B A A B B A X X A X A X A B B A X A X
B X A B B B A A A A A B A A A A B B A
A B
Satt567 B B B A B B A B A A A A B A B A B A
A A A A A A A A A A B A A X B B A A B
B A A B B A B B B A A A B A A A B B A
B B B A A A B A X B A A B B A A A A A
A A A B A B X B A A A A A A B A B B A
B B X A A B B A A A B B B B B A B A A
B B A A B A A A A B A B B A X B A A A
A A
Satt697 B B A A A A B A A B B B A B B B B A
B A B B B B B B B B A A A B A A A B B
B A B A A B A B X X B B A B B B A A A
A B A B B A A A B A B A B B A B B B B
A B B A B B B B B B B B B B B B B B B
A B B B B A A B A B A A B B B B A B B
B B B B B A B B B B A A A B A A B B A
A A
Sat_389 B A B A X B B B B B A A B A A A B A
A A A A A B A B A B B B A B X B B B B
B A X A A A B B B A A A B A A A A B A
B B A B A B A B A A A A A B A A B B A
B A A B B B A B A A A B A A B A B B A
B A A A B B B A B B A B A A A A B A A
B A A A A A A B A B A B B A A A A A A
A B
Satt245 A A B A B B B A A A A A B B B A B A
A B A B A A A A A A B A B A A B A B A
A A B A A A B A A A A B B B A A A A B
A A X B A A B A X A B A A B A A A B A

A A A B A A A B A X B A A A B A B A B
B B X A A B B A B B A A B A A A A A A
B A A A A A A A A B A B B A X B A A A
A A
Satt626 B A B B B B B A B A A B B B B B B A
A B B A B A A B B B B A B A A B B B A
A B B B A B B A A A B B B B B A B B A
B A B B A B B B A B A A A B A A A B A
B B B B B B B B B B B A A A B A B B A
B B A A A B A A B A B B B A A A B B A
B A A A A A A A B B B B B B A B A A A
A A
Satt536 B A B B B B B A B B A B A B A B B A
A B B A B B A B B B B A B A A B B A A
A B B B A B B A A B B A B B B A B B A
B A B B A B A B A B A A A B B X A B A
B B B A B X A A B B A A A A B A B B B
B B A A A B B A B A B B B A A A B B A
B A A A A A B A A A B A A B A B A A A
A A
Satt655 B A B B B B A B B B A B B B B B B B
A B B A B A A B B B B A B A A B B A A
A B B B B B B A A B B A B A B B B B B
A B A B A B B B A B A A A B B B A A A
B B B B B A A B A B A A A A X A A B B
B B A A A B A A B A B B A A A A B B B
B A A A A B B A A A B A A B A A X A A
A A
Sct_147 B A A A A B A B A B A B A B A B B A
A A B A B A A B B B A A A A A A A A A
A A B A A B A A A A B A A A B A B B A
A A A A A A A A B B A A A A B B A A A
A B B A B B B B B B A A A A B A B A B
A A A A A A B B A B A A A A A A A B A
B A A A A A B A A A B A A A A A A A B
A A
Sct_067 B B A B A A B A A A B A B A B A A B
B B A B A B B A A A B B A A A B A B A
A B A B B A B A A A A B B A A B A A B
B A B B B A B A A A B B A B B A B B B
A A A B A B B X A A A B A A B A A A B
B B A B B B A B A A B X B A B A A A B
A A A B A A A A A A A B B A A B B B B
A A

Satt589 B B A B B B B A B A B A B A A A B B
B A A A A A B A A A B B A A B B B A B
B A B A B A B A A B A A B B A B A B B
B B B B B B B B A A A B B B B A B A B
B A A B B A A A A A B A A A B A A A B
B A A B B B A B A B B A B B B A A A A
B B A B B B A A A B A A B A A B B B A
B A
Sat_129 X B A A B B B A B B A A A B A A B B
B B A A A A B A B A A A B A B A B A B
B B X B A A B B B B A A A A A B X X A
B B A A B B A B A X A B A B A X A A A
X B X B A B B B B B B B B B B A A A B
B B B B B B B A B B A B A B B A A A A
B B B A B A A B A A A A A A A B B A B
B B
Satt233 A A A A A A A B B A A A A A B A A A
B A A A A B A A A A A A B A B A B B B
B B A B A B B B B B A A A B A B A X A
X B B A A B B B X A A A A B B B A B B
B A B B B B A B B B B B A A B A B B B
B B A A A A A A B B A B A A A A B A A
B B A A B B A A A A A B A A A A A A A
B B
Satt424 X X A A A A A A B A A A A B A A B B
A B A A A B A B A B A A B X B A B B B
B A B A B A A B X X A A A B A X B X B
A B A A B B A B X B A A B A B A A B A
B A B A A B X A X X B X X A X A X A X
A X X X A X A X X A A A B X A A A X X
B B A A B X B A X X A A A A A A A A X
B B
Sat_294 B A A A A A A A A A A A A A A A B A
A A A A A A A A A A A A A A A A A A A
A A A A A A A A A A A A A A A B A A A
A A A A A A A A A B A A A A A A A A A
A A A A A A A A A A A A A A A A A A B
A A A A A A A A A A A A A A A A A A A
B A A A A A A A A A A A A A A A A A A
A A
Satt228 A A A A A A A A A A A A A A A A A A
A A A A A X A A A A A A A A A A A A A
A A B A A A A A A A A A A A A A A A A
A A A A A A A A A A A A A A A A A A A

A A A A A B B A A B A A A A A A A A B
A A A A A A A A A B A A A A A A A A A
B A A A A A A A A A A A A A A A A A A
A A
Satt177 B A B A B B A A A A A A A A A A B A
A A A A A A A A A A A A A B A A A A A
A A B A A A B A A A A A A A A A B A A
A A A A A A A A A B A A A A A A A A A
A A A A B B B A A A A A A A B A A A B
A A A A A A A A A B A B A A A A B A A
A A A A A A A B A A A B A A A A A A A
A A
Sat_215 A A B A B B A A A A A A A A A A B A
A A A A A A A A A A A A B A A A A A A
A A B B A A B A A A A A A A A A B B A
A A A A A A A A A A A A A B A A A A A
A A A B A B B A A A A A A A A A A A A
B A A A A B A A A B A B A A B A X A B
B A A B A X A A A A A B B A A A A A A
A A
Satt538 X A X X X X A X A A A A A A A A A X
A A A X A A A X A X A X A A A X X X A
A X B X X A X X X A A A A A A A X X X
X A X A A X X A A X X A A X A A A A A
A A X A A A B A X X A X X X X A A A B
X X A A A X X A X X X X X A A A X A X
B A A X A X X X A X A X X A A X A X X
X X
Satt378 B A B B B A A B A A A A A A A A A B
A A A B A A A B A B A A B A A B B B A
A B B B B B A B B B B A A A A A A B B
A A B A A B B A A B B A A A A A A B A
A B B B A A B A B B A B A A A A A B B
A A A A A B A A B A A A A A A A A A A
B A A A A B A A A A A B B A A B B A B
B B
Sat_119 A A A A A A A A A A A A A A A A A A
A A A A A A A A A A A A A A A A A A A
A A A A A A A A A A A A A A A B A A A
A A A A A A A A A A A A A A A A A A A
A A A A A A A A A A A A A A A A A A B
A A A A A A A A A A A A A A A A A A A
B A A A A A A A A A A A A A A A A A A
A A

Satt552 A A A A A A A A A A A A A A A A A A
A A A A A A A A A A A A A A A A A A A
A A A A A A A A A A A A A A A B A A A
A A A A A A A A A A A A A A A A A A A
A A A A A B A B A A A A A A A A A A B
A A A A A A A A A A A A A A A A A A A
A A A A A A A A A A A A A A A A A A A
A A
Sat_303 B A A A B A A A A A B A A B B A A B
B A A B A A A A A A A B B A A A B A A
A B A B B A A A A A B A A A B A B B X
B B B B A B B A A A B A B B B A A B B
A A B B B B B B B B B B A B B B B A B
X B A A A B B B B X A A A A A A A A A
A B A X A B B A A A A B A A A A X A A
A A
Satt358 B A B A A B A A A A A A A X A X A A
A A A A A A A A B A A B A A A A A A A
B A B A A A A A A A A A A A A A A B A
B A A A A A A A A A A A A B A A A A A
A A A B A B B B A A A B A A B A X A A
B A A A X X B A A B A B A A A A X A A
B A A A A A A X A A A B A A A A A A A
A A
Sat_291 B A A A A A A A A A A A A A A A A A
A A A A A A A A A A A A A A A A A A A
A A B A A A A A A A A A A A A B B A A
A A A A A A A A A B A A A A A A A A A
A B A A A A A B A A A A A A A A A A B
A A A A B A A A A B A A A A A A A A A
B A A A A A A A A A A A A A A A A A A
A A
Satt500 A A A A A A A A A A A A A A A A A A
A A A A A A A A A A A A A A A A A A A
A A A A A A A A A A A A A A A A A A A
A A A A A A A A A A A A A A A A A A A
A A A A A A A A A A A A A A A A A A A
A A A A A A A A A B A A A A A A A A A
B A A A A A A A A A A A A A A A A A A
A A
Satt153 A A A A A A A A A A A A A A A A A A
A A A A A A A A A A A A A A A A A A A
A A B A A A A A A A A A A A A B A A A
A A A A A A A A A A A A A A A A A A A

A A A A B B B A A B A A A A A A A A A
A A A A A A A A A A A A A A A A A A A
B A A A A A A A A A A A A A A A A A A
A A
Satt243 B B B B B B B B B B B B B B B B B B
B B B B B B B B B B B B B B B B B B B
B B B B B B B B B B B B B B B B B B B
B B B B B B B B B B B B B B B B B B B
B B B B A A A A B A B B B B B B B B B
B B B B B B B B B B B B B B B B B B B
A B B B B B B B B B B B B B B B B B B
B B
Sat_307 A A B A A B A B A B A B A A A B A A
B A B A B B B B B A A A B B A A A A B
B A A A B A A A B A A B A A B A A A A
A A B A A A A B B B A A A A A A B A A
B A B B A A B B A B B A B A B B A A A
A A X A A B B A B A A A A B A A A A B
A B B A A A A A A A A A B A A A A B A
A A
Sat_247 A A A A A A A A A A A A A A A A A A
A A A A A X A A A A A A A A A A A A A
A A A A A A A A A A A A A A A A B X A
A A A A A A A A B A A A A A A A A A A
A A A A A A X X A X A A A A X A A A B
A A A A A A A A A A A A A A A A A A A
B A A A A A A A A A A A A A A A A A A
A A
Satt484 B A A A A A A A A A A A A A A A B A
A A A A A A A A A A A A A A X A A A A
A A B A A A A A A A A A A A A A A X A
A A A A A A A A A A A A A A A A A A A
A A A A A B A B A A A A A X A A A B A
A A A A A A A A A A A A A A A A A A A
B A A A A A A A A A A A A A A A A A A
A A
Sat_270 B A A A B A A A A A A A A A A A B A
A A A A A A A A A A A A A A A A A A A
A A B A A A A A A A A A A A B A B A A
A A A A A A A A A B A A A A A A A A A
A A A A A A A B A B A A A B A A A A A
B A A A A A A B A A A B A A A A A A A
B A A A A A A A A B A A B A A A A A A
A A

Satt359 B A B A B B A A A A A A A B A A A B

B A A B A X B A B A A A B A A B A A A

A A B A A A B A A A A A A B A A B A B

A A B A A A A B X B B A A B A B A A B

A A A B A A A B B A B B A A A A A A B

B B B A A B B A B A B B B A A A X A A

B A A A X X A A X X A B B A X B A A A

B B

Satt197 B B B A B B A A A A A A A A A A A A

A B A A A B A A A A A A A A A B A B A

A A B B A A B A A A A B A A A A B X A

X A B A A A B A X B A A B B B B A B A

A A B B B B A B B B A B A A B A X A B

B B A A A B B A A A B B B B A A B A A

B B A A B B A A A B A B B A B B A A A

B B

Sat_200 A A A A A A A A A A A A A A A A A A

A A A A A A B A A A A A B A A A A A A

A A A A A A A A A A A A A A A A A A A

A A A B A A B A B A A A B A A A A A A

A A B A B A A A A A A A A A A A A A A

A A A A A A A A A A A A A A A A A A A

A A A A A A A A A A A A A A A A A A A

A A

Satt353 A A A A A A A A A A A A A A A A A A

A A A A A X A A A A A A A A A A A A A

A A A A A A A A A A A A A A A A A A A

A A A A A A A A A A A A A A A A A A A

A A A A X A X A A X A A A A A A A A A

A A A A A A A A A X A A A A A A A A A

A A A A A A X A A A A A A A A A A A A

A A

Satt434 B A B A B B B B A A A A A B B A B B

A A A A A A A B A B A A A B A A A A A

A A A A B A B B B A A B A B A A A X B

X A A B A A A A A A A A A B A A A A A

A A A B B B A B B B B A A A B A B B A

B B A A A A B A A B A A A A A A A A A

B A A A A A A A A A A A B A A A A A A

A A

Satt293 B A B A B B B A A A B A B B A A B B

A B A B A B B B A B B B A X A B A A A

X A B A B A B B B A X B B B A B A A B

B A B B A A B A A A B B A B B B B A A

X A B B A A A B B B B A A A B A B B A
B B A B B B X A A A B B B A B A B A B
X A A B A A A A X X A B B A B A A B X
X A
Satt314 X X B B B B A A B A X A A B B A B B
A A A B A B A A A A A A B A A B B B A
A B B B B A B A A A A A A B A A B B A
A A B A A B B B B B B A A B A B A B A
B A B B B A A A B A B A A A A A A A B
B B A B A B A A B A B B B A A A B A A
B A A A A B A A A B A B B A A B A A B
B B
Satt114 A B B A B B A B B A A A B B B A A B
A A B A X A A A A B A B X A B B B B A
A B A X B X A A A B B B B A A B A B X
B A A B A A A A A A A B B A A A A A A
A A A A X X X B A X A A A A A A A A B
A A A A A A A B B B A A A B A A A A A
B A A A A A A A A A A A B A A B A A A
A A
GMRUBP A A A A A A A A A X A A A A A A A A
A A A A A A A A A A A A A A A A A A A
A A A A A A A A A A A A A A A X A A A
A A A A A A A A A A A A A A A A A A A
A A A A A X A A A X A A A A A A A A A
B A A A A A A A A B A A A A A A A A A
B A A A A A X A A A A A A A A A A A A
A A
Sat_262 B B A A A B A A A A A A A A A A A A
A A A A A A A B A B A B A A B A A B B
B A B A A A B B B B A A A A A B B A A
A B A A A A A A B B A A B B A A A B A
A A A B B B A A A B A A A A B A A A B
A A A A A B A A A A A A A B A B A A A
B A A A A A B X A A A B A A A A A A A
A A
Satt030 A X B A B B B A X A A A A A B A A B
A A A B A A A B A B A A X A X A X X X
X A X A B A B X X B A A A B A A B B B
B X A B A B A A A B A A X B A B A X A
X A B B A B A A B B B B A X B A B B A
B A A A A A B A X B A B A X A A X X A
A X A A B B A A X X X A A A A A A A A
B B

Satt425 B A B B B B A A B A A A A B B B B B
A A A B A B A A A A A A B A A B B B A
A B B B B A B A A A A A A B A A B A B
A A B A A X B B A B B A A B A B A B A
B A B B B A A A B X B B A A X A A A B
B B X A X B X A B A B B X A A A A A X
B A A A B X A A B B A B B A X A A A B
B B
Sat_039 B B A A A A B B B A A A A B B A A B
A B A B B A A A A A A A B A B B B A B
B A B A B A A A A A A B A B A A B A A
A B B B A B B B A B A A B A A A A A A
B A B A B B A A B B B A A A B A B A B
A B A A A B A A B A B A B B A A A A A
A B A B B B A A B B A A B A B B A A B
B B
SOYHSP176 A X A A B A A A A A B A A B A A A B
B B A A A B B A A A A B B B A B A A B
B A B A X A A B B B A A A A A B B A B
A X B A B A B A B A B B A A A A B A B
A A B A B B A A B B A B A A A A A A B
A B B A A B A B B A B B B B A A B A A
B A A B A B B B A A B A B A B B A X B
B B
Satt334 A B A B B X A A A A B A A B A A B B
B B A B A B B A A A A B B B A B A A B
B A B A B X A B B B A A A A A B B X B
B B B A B A B A B A B B B A A B B A B
A A B A B B A B B B A B B A A A A A B
A B B A X B A B B A B B B B A A B B B
B B A B B B B A A B A A B A B B A B B
B B
Satt510 B B A B B A A A A A B A A B A B B A
B B A B A A B A A A A B B B A B A A B
B A B B B A A B B A A A A A A B B A A
A B B A B A B A A B B B B A A A B A B
A A B A A A B A B B B A A A A A A A B
B A A B B B A B B A B A B B B A B A B
B A B B B B X B A A B A A A A B B B A
B B
Satt063 A A A A A A A A A A A A A A A B A A
A A A A A A A A A A A A A A A A A A A
A A B A A A A A A A A A A A A X A A A
A A A A A A A A A A A A A A A A A A A

A A A A A A A A A A A A A A A X A A A
A A A A A A A A A A A A A A A A A A A
B A A A A A A A A A A A A A A A A A A
A A
Sct_094 B A B A B B A A B A A A B B A B A A
A B A A A A A A A A B A A A A A B A A
A A B A A A B A A A A B B A A A B B A
B A A A A B A B A B A A A A A A A A A
B A B B A A B B B B A A A A X A A A A
B A A A A A A A A A A X A A A A X A A
B A A A A A A A A A A B A A A A A A A
A A
Satt474 B A B A B B A A B A A A B B A A B A
A B A A A A A A A A B A A B A A B A A
A A B A A A B A A A A B B A A A B A A
B A A A A B A B A B A A A A A A A A A
B A B B A A B B B B A A A A A A A A A
B A A A A A A A A A A B A A A A A A A
B A A A A A A A A A A B A A A A A A A
A A
Satt070 B A B A B B A A B A A A B B A A B A
A B A A A A A A A A B A A B A A B A A
A A B A A A B A A A X B B A A A B B A
B B A A A B A B A B A A A A A A A A A
B A B B A A B B X B A A A A X A A A A
B A A A A A A A A A A B A A A A A A A
A A A A A X B A A A A A A A A A A A A
A A
Satt720 A B B A B A A B A A A A A B A A A A
A A A B A B A B A B A B B A B A A A B
B B B B B A B A A A A A B B A B B X X
A A A B B A A A X B A A B A A B A A B
A A B B A B A B B A B A A A B A B B B
B B B A A A A B B B A B A B A A B A A
B B A A B A B A B B A B A A A A A A B
A A
Satt651 A B B A X B B B B A B A A A A A A A
B A A B A B B B A B A B A A A B B B B
X A A A A A B A A X A B A B A X A X A
X B B B B B B B A B B B X B B B B X B
B A B B A B X A B X X X X A X A X B X
B X A B B B X B B X A A A A B A B A X
B B A B B X X A X X A B A A X B B B X
A A

Satt553 A B X A B B X A A A B A B A A B B A

B B A A A B B A A B B B A B A X A B A

A A A A A A X B A A A A B B X B B B A

B A B A B A B A A A B B B B A B B B B

A A A X B A A B A B B B B A A A B B A

A B A B B B A B A B B A A B B A B B A

B B A B B B B A A B A X A A A B B X A

B B

Satt231 B B A A B A A A A A B A B A A A B A

B B A A A B B A A A B B A A A B A B A

A A B A A A X B B B B A B B A B B B X

A A B A B A B A B B A B B A A A B B B

A A A B B B A B A B B B A A A B B B B

A B A B B B A B A B B B X B B A B A B

B B A B B B A A A A A X B A A B B B A

B B

Satt483 A A A A B A A A A A B A A B A A B B

B B A A A B A A A B A A B B A A A B B

A B A B A A B B A A A A A A A B B A B

B A B A B A B B B A B B A B A A B B B

B A A B A B A A B B A B A A A A A B B

A B A A A B A B B A B A B A A A B B A

B A B B A B B A A B A B B A A B B A A

B B

Satt183 B A A A A A A B A A B A A A A A A A

B A A B A B B B A B A B A A B B A A A

A B B B B B B A A B B B B A B A X B A

A B B A A B B A B A A A B B B B A B A

B B A B B A A A A A A A B B A A A A B

A A A B A A A B A B B A B A B A B A A

B A A B A A B B B A X A B B B B B B A

A X

Sct_193 A B B B B A B A B B A B B B A B B A

B A A B A X A A A B A B A A B A B A B

B A A B A A B B B X A B B A B A B A B

B B B B B B B A X A B B A A A B A A B

A B B A B A A A A A A B B B B B B B A

A B B A B X B A B B A B B B B B B B B

A B B A A B B B B B B A A B B A A A B

B B

Sat_255 A A B A B B B B A A B A B B A A B B

B A A B A A A A B A A A A X A B A A B

B A B A B A B B B B A B B A B A X B A

B B B B B B B A X A B B A A A B B B B

A	A	X	X	A	A	A	A	A	B	B	B	A	A	B	A	X	X	B
A	B	B	A	B	A	A	A	A	A	A	A	A	A	A	B	A	B	X
A	A	B	A	X	A	B	B	B	A	B	A	B	A	B	A	A	A	A
A	A																	
Satt620	B	B	B	A	B	B	B	B	A	A	B	A	B	B	B	A	B	B
B	A	A	B	A	B	B	B	A	B	B	B	B	A	B	B	A	B	B
B	A	B	A	B	A	B	B	B	B	A	X	X	X	X	X	X	X	X
X	X	X	X	B	A	B	A	A	B	B	B	B	A	A	B	B	B	B
A	A	B	B	B	B	A	A	B	B	A	B	B	B	B	B	B	A	A
A	B	A	B	B	B	B	X	X	X	X	X	X	X	X	X	X	X	X
A	B	A	B	A	A	B	A	B	A	A	B	B	A	A	B	B	B	B
B	B																	
Sat_224	B	A	B	A	A	B	A	A	A	A	A	A	A	A	A	A	B	A
A	A	A	A	A	A	A	A	A	A	A	A	A	B	A	A	A	A	A
A	A	A	A	A	A	B	A	A	A	A	A	A	A	A	A	A	B	A
B	A	A	B	A	A	A	A	A	X	A	A	A	B	A	A	A	A	A
A	A	A	B	A	B	B	B	A	A	A	B	A	A	B	A	X	A	B
B	A	A	A	A	A	B	A	A	A	A	B	A	A	A	A	B	A	A
A	A	A	A	A	A	A	X	A	A	A	B	A	A	A	A	A	A	A
A	A																	
Satt654	A	A	A	A	A	A	A	A	A	A	A	A	A	A	A	A	A	A
A	A	A	A	A	A	A	A	A	A	A	A	A	A	A	A	A	A	A
A	A	A	A	A	A	A	A	A	A	A	A	A	A	A	X	A	A	A
A	A	A	A	A	A	A	A	A	A	A	A	A	A	A	A	A	A	A
A	A	A	A	X	X	X	B	A	X	A	A	A	A	A	A	A	A	B
A	A	A	A	A	A	A	A	A	B	A	A	A	A	A	A	A	A	A
B	A	A	A	A	A	X	A	A	A	A	A	A	A	A	A	A	A	A
A	A																	
Sat_350	B	A	A	A	B	A	B	B	A	A	A	A	B	B	B	A	B	A
A	A	A	A	B	A	A	A	A	A	A	B	B	A	A	A	B	B	B
B	A	B	A	A	A	A	B	B	B	A	B	B	A	A	B	A	A	B
A	A	A	B	A	A	A	A	A	A	B	A	A	A	A	A	A	A	A
A	A	A	A	A	B	A	B	A	B	A	A	A	A	A	B	X	B	B
A	A	A	A	A	A	A	A	A	A	A	A	A	A	A	A	A	A	A
B	A	A	A	A	A	A	A	A	A	A	A	A	A	A	A	A	A	A
A	A																	
Satt414	B	B	B	A	B	B	A	B	B	B	B	B	B	B	B	A	A	B
A	B	A	A	X	X	A	A	A	A	B	A	B	A	A	A	A	A	B
A	B	A	B	B	A	A	A	B	B	B	B	B	B	A	A	A	A	B
A	A	A	B	A	A	A	A	A	B	A	A	B	A	A	X	A	A	A
A	B	A	A	A	A	B	X	A	B	A	A	A	A	B	A	B	A	X
A	B	B	A	A	A	A	A	B	A	A	A	A	B	A	A	A	A	A
X	B	A	A	B	B	A	A	A	A	A	A	A	A	A	A	A	A	A
A	A																	

Satt596 B A A B X A A A B B B B A A A B A A
B B B B B B B B B B A B A B B B B B B
B A A A X B A B B A A A A B B B B A B
A B B A B B B B A B B B A A B B A A A
A A A A B B B B A B A A A A A A A A B
A A B B B B B B A B A A A A B A A A A
B A B B A A B B B A B A B B B B B B A
A A
Sat_022 B B A A A A B A B A A A A A A A B A
A B A A B A A B A B A A A A B A B B B
B A X A A A A B B B A A A B A A A A B
A B A B B A B A B A B B A B A X A B A
B A A A B A A A A X B X X A B A B B A
A A A A B A A B B B A A A X A A A A A
A B B A B B A A X X A A A A A A A A A
B B
Sat_326 A B A A A A B A B A A A A A B A B A
B B A A A A A B A B A A X A B A B B B
B A A A A A A B B B A A A B A A B A A
A B A B A B A B A B A A B A B B A B B
B A A A X X A A A A B B X A B A B B A
A A A A A A B A B B X A B B A A A A A
B B A A B B A A A X B A A A A A A B B
B B
Sat_001 A B A A A A B A B A A A B A X A B B
A B A A A A A A A A A X X A B B B B B
B A B A B X A A A A A B A A A A B A A
A A B B B B X B A X A A B A A A A B A
B A B A X A A B B B A A A A A B A B A
B A A A A A A A B A B A B B A A A A A
B B A A B B A A A B A A B A A B A A A
B B
Sat_194 A B A A A A B A B A A A B A A A B B
A B A A A A A A A A B A B A B B B B B
B A A A B A A A A B A A B A A A B X X
B B B B A B B B A B X B A A A A A B A
B A A A B X X B A X X A A A A B A B A
A B A A A B A A B A B A B B A A A A A
A B A A B B A A A B A A B A X B A A B
B B
Satt669 A A B A B A A A A A A A B A A A A B
A A A A A A A B B A B B A B A B B B B
B A A B A A A B A A A A B A A B A A B
X B B A B A A B A A A A B A A A A B B

A A B A A B A A B B A X A A A X A A A

A B A A A X A B B B B X B B A A A A A

A B A A B B B A A B A A A A B B A A B

A A

Sat_292 A A A A A A A A A A A A A A A A A A

A A A A A A A A A A A A A A A A A A A

A A A A A A A A A A A A A A A A A A B

A A A A A A A A A A A A A A A A A A A

A A A A A A A B A B A A A A A A B A A

A A A A A A A A A B A A A A A A A A A

B A A A A A B A A A A A A A A A A A A

A A

Sat_284 B B A A A A A A A A A A A A A A B A

A A A A A A A A A A A A A B A A A A A

A A B A A A A A A A A A A A A A B A A

A A A A A A A A A B A A A A A A A A A

A A A A A A A A A A A A A A A A A A A

A A A A A A A B A A A A A A A A A A A

B A A A A A A A A A A A A A A A A A A

A A

Sct_192 B B B B A B B B B B B B B B B A A B

B B B B B B B B B B B B B A B B B B B

B B B B B B A B B B B B B B B B A X B

B B B B B B B B B A B B B B B X B B A

X B B B B B A B B B A B B B B B B B A

B B B B B B B B B B B B B B B B B B B

A B B B B B B B A B B B B B B B B B B

B B

Sat_333 A B B A B B A B B A A A A A A A B B

A B A A A B A A A A A A A B A A B A A

B A A A B A B B B A X B A A A A B A A

A B X A A B A B X A A A B B B A A A A

B A B B A A B A B B A B A A B A B A B

B A A A A B A A A A A B A A A A B A X

A B A B B X A A A X A B A A A A A A B

A A

Satt186 B B B B B B A B B A A A B A A A B A

B A A B A X A A A A B A B A A B B A B

B B A B B A B A A A A A B B A A A B A

B B A A B B A B A A A A B B A B A B B

B A B B B A A B B B B B A A X A X A B

B X A A X X B B B X A B A B A A B A X

B A A A B B X A X X A A B A A A A A B

B B

AW734137 B A B A B B B A B A A A B B A B B B
B B B A B B A B A B A A B A A B A B A
A B B A A A B A A A A B A A A A B A A
A A A B B B B A B A B X A B A A A B A
B A A B A A A A A A A A A A B A B B B
A B B B A B B X A A A A A B A A A B A
X A A B B A A B B A A B A A A B B A A
B A

Sat_210 B A A A A A B A B A A A B B B A A A
A A B A B A A A A A B A B A A A B B A
A B A A A A B B B A A B B B A A A A B
A A A B A B A B A A A A B A A A A B A
B A A B A A A A A A B A B A B A A B B
A A A A A A A A B A A B A B A A B A A
A A A A A B A A A A A B A A A A A A A
A A

Satt688 B A B A B B B A B A A A A B B A A A
A A A B A X A B A A A A B A A A B B A
A A B A A A A B A A A B A A A A A A X
A A A B A B A B X A B A A B B B A B A
B A A X A A A A A A A A A A B X B B A
B A A A X A X A B A A B A A A A B A A
A A A A A B B A A X A B A A A A A A A
B B

AZ254740 B A B A B B B A B A A A B B B A A A
A A A B A B A B B A B X B A A A B B A
A B A A A A B A A A A B B A A A A B A
X A A B A B A A A A A A B B B B A A B
A B A B A A A A A A A B A A B A B B B
B A A A A A A A B A A B A A A A B B A
X B A A B A A A A A A B A A A A A A A
B B

Satt570 B A A A A A B A B A A A B B B A A A
A A A B A B A B A B B A B A A A B B A
A B A A A A A A A A A B B A A A A B B
A A A B A B B A X A B A A A B B A B A
B A A B A A A A A A A A A A B A B B A
A A A A A A A A B A A A A A A A X A A
B A A A A X X A A A A A A A A A A A A
B B

Satt610 X A A A A A B A B A A A X B B A A A
A A A B A X A A A B A A A A A A A X A
A A A X A A X A A A B X B A A A B A X
A X A A A B A B A B B A A X X A A X A

X A A A A A A A X X A X A A X X X B X
A A A A X A A A B A A X B A A A X A A
B A A A A B X B A B A A A A A A A A A
B B
Satt612 B A A A A A A A A A A A A A A A B A
A A A A A A A A B A B A A B A A A A A
B A B A A B A A B B B A A A A A B X A
A A A A A A A A A B A A A B A B B B B
B B A X B A B B A A A B A A B A A B B
A A A A A A A A A A A A A A A A A A A
B A A A A A A B A B A A A A A A A A A
A A
Satt501 A A B A B X A B A A B B B B A A A A
A B A A A A A A A B A A A A A A A A A
A A A A A A B A A A A A A A A A A B B
A B A A A A A A A A B A A B A A A A A
B A A B A A A B A A A A A A A A A A B
B A A A A A B A A B A B A A A A B A B
B B A A A A A A A A A B A A A A A A A
A A
Sat_203 A A B B B B A B A A A A A A B A B A
B A A B A A A A A A A A B A A B A B B
A B B B A A B A B A A A A B A A B X A
A A B A B A B A A B B B A B B A A B B
A A B B X A X A B A A B A A A A B B A
B A A A A A A B B X B B A A A A A A A
B A A A A A A A A A A B B A A B A B B
A A
Satt503 A A B B B B A A A A A A B A B A A A
B A A B A A A B A B A A B A B B A B B
B B A B A A B B B A A B A A A B A X A
A A B A B A B A B A B B B B B B A B B
A A B B B A A A B B A B B B X B A A B
B B B A A B B B B B B B B A A B B A A
B A B A A B B B B A A B B A B B A A A
B B
Satt288 B B A A B B B A A B B B A A B B B B
A B B A B B B A B A A B A X B B A A B
B A B A B B B A A X B B A B B X B B A
B B B B A A B A A X A B B A B A B A A
A B A A A A A A A A B X A A X A B B A
A A A B B B A A A A B A B B B A A B B
B B A B B A X A A A X A B B A B B B B
A A

Sat_372 A B B B A B A A B A A A B B A A X B
B A A B A A A B A B B A B A A B A B A
X B A B B A B A A B X B B A A A X A B
B A A A B B A B A B B A X A X B A B B
B A A B B A X B X A B X A A X A A A B
B B X A X B X B B X A B A B A A X X X
B B A A B X A A A A A A B A A B A A B
A A

Sct_199 A B A A B A B B A B A B A A A B X B
A B B A B B A B B X X A A B B B A A B
B A A A B B A A A B B A A B B A X X A
B A B B A A B A B X A A B A B A A X A
A B A A X B B X X A A X X A X X A B B
A B B A A X A B A A A X A B A B A X A
A A B A B B B B B B B A B B B B A A B
B B

Satt448 A B A A A A B A B A A A B A A A B B
A B B B A A A A A X B A B A B B B B B
B A B A B A X A A A A A B A A X B X A
B B B B A B B B A B A A B A A A A B A
B A B A B A X A A A B A A A A A B X A
A A A A A X A A B A B A B B A A A A A
B B A A B B A A A B A A B A A B A A B
B B

Satt373 A A B B B B A B B B B B A B B A B A
A A A B A X B B B B A B B A B A B A B
B B A B B B B A A A B A A A B B A X B
B B A A B B A B X A A B A B B X B A B
X B B B X B B A B X A B A A A A A A B
B A A B B A A B B A A B A A B A B B B
B A A B A B B A A B B B A B A A B A A
A A

Satt313 A A B B B B B B B A B A A B B A B A
A B A A A X B B A B B B A B B A B B B
B B B B A A B B B B B B B A A B A A A
A B A A A B A B A A A B A B A A B B A
B A B B A B A A B B A B A A B A B B A
B B A B B X X X X B A B A A B A B A B
A A A A A B X A A X A B A A A A X B X
X B

Sat_191 B A B B B B B B B A B A B A B A B A
A B A A A B B B A A B B A B B A B B B
B B B A A A B B B A A B B A A B A B B
B B B B A B A B A A A B A B B X B B A

B A B B X B X A B B A B A A B A B B A
B B A B B A B B B A B A B A B A B A B
A A A A A B X A B A B A B B B B A B A
B B
Sat_134 A A X B B A B A B A B A B A X A A A
A B A A A B B B A B B B A A B A B A A
A B B B B B B A A B B B B A A B B B B
A A B B A B A B B B B A A A A A B B A
B A A B X X B A A A A B A A B A B B A
B B A B B A B X A B A A A A B A A A B
A A A A A B A A A B A A A A A A B B B
B B
Satt182 B A A B B B B B B A A A B A B B A A
B B A A A B A B A B B A A A B A B A B
B B A B A A B B B B A B B B A A A A B
B B A B A B A B A A A A A B A A A A B
B A A B B A A A A X A A A A B A B B A
B A A A A A X B A B A B A A A A B A A
B A A A A X A A A A A B A A A A A A A
B B
Satt497 X X X X X X X X X X X X A A A A A B
A A A A A A A A A A A A A A A A B A A
A A B A B A A B B A A B A B B B B A B
A A A B A B A B A B A A A X B B A A B
B A B X B A A B B B B B B B B A B B B
B B A A A A A A A A A B A A A A B A A
B A A A A A A A X B A B A A A A A A B
B B
Sat_099 A A A B A A B A A A A A A A A A A B
A A B A A A A A A A A A A A A A A A A
A A A A A A A A A A A A A A A A B A A
A A A A A A A A A B A A A A A A A A A
A A A A A A A B A A A A A A A A A A B
A A A A A A A A A B A A A A A A A A A
B A A A A A A A A A A A A A A A A A A
A A
Satt229 B A B A B B A A B A A A B A B B B B
B B B A A A A A A B A X X B A A A A A
X A A A B A X A A A A A A A A X A X A
X A A A A A A A A A A B A A A X B B A
A A A B A A A A A A B B B B A A A A A
A A A A A A X A B X A B A A A A A X X
X A A B A X X X B X A A A A A A A A B
B B

Satt292 B B A A A A A A A A A A A A B B B A
A B A B A B B B A A A A B A B A B A A
A A A B A A A A A A B B B A A A A A B
A A A A A B A B B A A B B B B A B B B
A A A A A B A A X A A A A A A A A A B
B B A A A B A A A A B A A B A B A A A
B A A A A A B A A A A A A A A B A A A
A B
Satt571 A A A A B A A A A A A A A A B A A A
B A A A A A A A A A A A A A A B A A A
A A A A A A A A A A B B A A A A A A A
A A A A A A A A A A A A A A A A A A A
A B A A A A B B A A A A A A A A A A A
A A A A A A A A A B A A A A A A A B A
B A A A A A A A A A B A A A A A A A A
A A
GMGLPSI2 A B A A B A A A A A A A A A A A B A
A A A A A A A A A B A A A A A A A A A
A A A A A A A A A A A A A A A A A B A
B A A A A A A A B A A A A B A A A A A
A A A B A A A B A A A A A A B A A A B
B A A A A A B A A A A B A A A A B A A
B A A A A A A A A A A B A A A A A A A
A A
Sct_189 B A B A B B A A A A X X A A A A A B
A A A A A A A A A A A A B A A A A A A
A A A A A A B B A A A A A A A B B B B
A A A A A A A A A A A A A A B A A A A
A A A A B A A B A B A B A A B A A A A
B A A A X A B A B A A B A A A A A A A
B B A A A A A A A B A B A A A A A A A
A A
Satt354 B A A B X A A A X A A A A X A A B A
A X A A A A A A A A A A A B X B X A X
B B B B X A A B X A A B B A B B B B A
A X B A A X A X A B A A A A B A A A A
A A A A B X B A B A A A B A X A A A A
A A A A A A A A A B A A A A A A A A B
B A A A A A A A A A A B B X A A A A A
A A
Satt239 A X A B A A A A B A A A B B A A B A
A B A A A X A A A A B A A B A A B A A
A B A B A A A B B A A A B A A A A B B
A B A A B A B A A B A A B A A A A A A

B A A A A A B A A A A A A A X A A A A
A X A A A X A A A B A A A B A A A X A
B B A A B A A A A A A A A A A A A A A
A A
Satt367 A B B B A B B B A A B X B B B A B X
B B A A A A B A A A B A B A A A A X B
X B A B A A B B B A A A B B A A A B A
A B A A A B A A A A A A B B A A A B B
A A A B A A A A A A B B A A B A A A A
B B B B A X B B B A A A B A X B A A A
A B A A A B A A A A A B A A A A A A A
A A
Satt270 A A B A A B B B B A A A A B B A B A
A B A A A A A A A A A A A B B B B B B
B B B X X X X X X X X B A A A A B X B
A B B B A B B B A B A A A B A B A B A
B A X A A A A A A A A A A A X X X X X
X B A A A B B A B A B B B A A A A A A
A A A A A A A A A X A B B A A B A A A
A A
Sat_170 A A B A A B A B A B A A A B B A B A
A A A A A A B B A B A A A A A A B B A
A A B A A A B A B A A B A A A A A X B
A B A B A B A B A X A A A B B B A A A
B A A A A X B B B B A B B B B B A A B
A A A B B A B A A A A A A A B A B A B
B A A A A B B A A B A B A A A X B A A
A A
Satt330 B X X B X X X A A B B B B A X X B X
B X B B A A A X X X A B A A A B B X X
X X X A X A X A A B A B A B B B B B X
B A A B A B A B B A A A A X B B X B A
B A B A A A A B B A A X A X B A B X X
X B B X X A B A A A A B B A X A B A X
A A A A A X A A A A A X A A A A X X X
X X

Phenotypic Data

PNUA 1 3 1 2 1 1 1 1 1 2 3 1 4 1 2 2 1 -100 1
1 1 2 1 3 -100 2 1 3 1 2 2 1 2 1 -100 -100 1 1
2 1 3 1 1 1 1 1 -100 3 1 3 1 3 3 -100 1 1 1
1 1 1 3 2 1 3 1 2 1 1 1 1 -100 1 2 2 1 2
1 1 3 1 3 1 2 1 2 4 1 1 1 1 1 1 1 3 2

	2	1	1	1	1	4	1	2	1	2	2	1	2	2	−100	4	3	1	4
	4	1	2	2	1	2	3	1	1	3	3	2	1	1	−100	2	1	1	2
	1																		
PNMA	1	2	1	1	1	1	1	2	1	1	3	3	2	2	1	1	4	−100	1
	1	2	1	2	2	−100	2	2	2	1	4	1	1	4	2	−100	−100	1	1
	2	1	5	1	1	1	2	4	−100	1	1	1	2	1	1	−100	2	1	2
	1	1	1	1	5	1	1	1	4	1	2	1	2	−100	5	2	1	1	4
	2	2	1	2	1	1	1	4	1	1	1	1	2	1	3	1	5	1	5
	2	1	1	1	1	2	2	1	5	2	1	1	5	1	−100	4	1	1	2
	1	1	3	1	3	2	2	1	1	3	6	1	1	1	−100	1	1	1	1
	5																		
PNBA	1	3	1	1	3	1	1	1	1	1	1	1	3	1	1	1	1	−100	2
	1	2	1	2	3	−100	1	3	2	1	1	3	1	1	3	−100	−100	1	1
	2	1	2	1	2	1	2	2	−100	3	1	3	1	1	1	−100	1	1	2
	2	1	1	1	1	1	1	1	2	1	2	1	2	−100	1	1	1	2	3
	1	1	3	1	1	1	1	1	2	2	1	1	3	1	2	1	1	1	1
	1	1	1	1	2	3	1	1	1	3	5	2	3	1	−100	3	1	1	2
	2	1	1	1	2	2	2	1	1	2	2	1	1	2	−100	1	1	2	2
	1																		
PNUB	1	6	1	3	4	2	3	1	2	1	1	5	6	1	2	4	3	−100	3
	5	8	4	6	5	−100	4	2	1	1	8	6	6	1	3	−100	−100	2	2
	4	4	5	2	3	2	4	3	−100	6	3	5	3	7	2	−100	1	4	9
	3	1	5	1	2	5	4	3	5	6	3	7	3	−100	3	4	3	2	4
	2	7	4	7	3	2	3	7	2	2	4	2	5	5	4	1	6	5	5
	3	2	3	6	3	4	4	5	7	3	6	2	4	4	−100	8	3	3	4
	2	3	3	2	2	4	11	7	2	4	5	3	2	2	−100	3	2	2	4
	2																		
PNMB	4	8	1	8	9	4	3	1	2	4	8	8	4	1	4	4	4	−100	3
	8	10	4	6	7	−100	4	6	4	2	8	7	4	3	5	−100	−100	3	2
	9	1	3	1	8	2	4	4	−100	6	2	8	7	6	2	−100	6	2	10
	1	3	2	1	5	4	3	5	10	5	5	2	3	−100	11	5	2	1	6
	2	5	8	6	9	2	4	11	7	5	1	3	10	3	5	2	6	5	8
	9	2	4	10	4	3	3	2	4	7	10	5	5	1	−100	9	5	4	12
	7	1	2	5	2	4	7	1	1	10	9	2	1	2	−100	2	2	5	4
	5																		
PNBB	2	3	1	3	4	3	3	3	1	4	5	4	9	4	7	2	4	−100	2
	2	6	7	5	8	−100	3	6	2	1	5	8	3	2	2	−100	−100	1	2
	4	3	3	4	4	5	5	5	−100	7	1	5	4	1	2	−100	2	4	3
	3	5	1	1	1	3	1	3	4	2	7	2	2	−100	7	6	2	2	5
	1	7	2	2	6	3	8	5	6	7	2	1	5	2	4	4	5	3	7
	2	1	1	3	3	6	2	7	6	2	4	6	6	2	−100	6	1	1	8
	6	2	4	4	4	6	5	2	3	6	7	1	2	2	−100	3	3	2	6
	3																		
PNUC	7	5	4	4	6	5	8	6	3	7	6	9	8	8	10	4	9	−100	3

	16	10	4	11	5	-100	5	5	6	4	14	12	3	13	5	-100	-100	3	6
	5	11	9	5	6	7	7	11	-100	5	5	4	7	8	1	-100	5	4	4
	3	3	6	1	7	14	8	5	6	8	7	4	3	-100	13	9	7	8	6
	2	8	10	6	5	6	4	3	6	5	3	6	9	7	6	4	2	6	5
	5	3	5	9	3	4	3	9	5	2	7	8	3	3	-100	3	6	2	5
	3	4	1	5	2	3	7	7	5	5	6	2	4	5	-100	3	3	4	7
	5																		
PNMC	10	8	3	10	6	7	14	10	3	7	15	9	19	11	11	4	3	-100	5
	7	14	15	10	5	-100	5	10	10	5	14	21	6	19	12	-100	-100	2	5
	5	13	10	10	4	16	6	13	-100	8	5	4	11	11	6	-100	16	10	6
	2	5	10	8	10	16	11	5	8	10	11	7	3	-100	9	12	12	9	9
	4	6	15	8	7	6	6	5	11	19	9	8	5	6	10	11	5	6	5
	11	6	7	8	5	4	10	7	8	3	6	11	8	5	-100	6	12	2	5
	2	5	3	15	3	8	4	4	5	6	9	9	4	14	-100	15	2	12	9
	1																		
PNBC	5	5	6	6	7	2	10	5	2	5	13	5	6	4	15	2	2	-100	1
	6	8	4	3	4	-100	2	5	16	3	8	10	2	6	2	-100	-100	4	5
	3	7	10	2	2	11	2	7	-100	2	6	2	6	9	2	-100	7	5	5
	3	2	3	4	7	13	10	4	6	6	1	7	1	-100	5	7	5	11	1
	4	3	8	9	9	12	5	6	3	5	4	5	3	5	11	4	7	3	10
	8	8	1	5	3	1	6	7	5	3	4	10	11	5	-100	9	6	3	8
	2	8	2	10	1	2	3	3	2	4	3	5	2	6	-100	4	2	4	5
	6																		
PNUD	4	1	1	5	1	2	6	4	3	1	1	1	2	3	7	1	7	-100	1
	2	1	2	14	1	-100	1	1	1	1	2	4	1	4	4	-100	-100	2	4
	2	9	6	2	1	4	1	2	-100	1	2	1	1	1	3	-100	7	1	1
	2	7	1	8	4	5	4	1	1	6	1	1	1	-100	1	2	1	2	1
	3	1	1	1	1	6	1	2	1	4	1	1	6	1	7	4	3	1	1
	5	1	5	9	1	1	1	5	1	1	1	1	2	4	-100	1	2	1	1
	1	2	2	2	1	1	1	1	2	2	1	2	1	3	-100	2	3	4	1
	1																		
PNMD	6	2	2	2	1	1	2	5	3	1	2	4	1	2	10	1	4	-100	1
	3	1	1	8	1	-100	1	2	3	1	3	7	1	6	3	-100	-100	2	8
	3	9	6	2	2	4	1	4	-100	1	1	1	1	1	1	-100	5	3	1
	3	3	2	4	5	1	8	1	1	4	1	1	1	-100	1	1	1	5	2
	2	5	5	1	2	13	1	1	2	1	1	1	1	1	9	2	3	1	1
	3	1	6	3	1	1	3	20	1	1	1	1	2	4	-100	1	1	2	1
	1	3	1	3	1	1	1	7	4	1	3	1	1	4	-100	1	1	4	1
	1																		
PNBD	3	1	2	2	1	1	1	1	3	2	1	1	1	5	4	1	2	-100	1
	2	1	1	1	1	-100	1	1	1	1	1	1	1	4	2	-100	-100	1	1
	5	6	2	1	1	4	1	1	-100	1	1	1	2	1	1	-100	2	2	1
	1	1	4	5	3	2	2	1	1	1	1	2	1	-100	1	1	1	2	1
	3	1	6	1	1	1	1	1	1	2	1	1	1	1	8	3	2	1	1

4 1 6 2 1 1 5 10 1 1 1 1 1 2 −100 1 2 3 1
1 1 1 1 1 1 1 5 2 1 1 1 1 3 −100 1 2 2 1
1

TPA 3 8 3 4 5 3 3 4 3 4 7 5 9 4 4 4 6 −100 4
3 5 4 5 8 −100 5 6 7 3 7 6 3 7 6 −100 −100 3 3
6 3 10 3 4 3 5 7 −100 7 3 7 4 5 5 −100 4 3 5
4 3 3 5 8 3 5 3 8 3 5 3 5 −100 7 5 4 4 9
4 4 7 4 5 3 4 6 5 7 3 3 6 3 6 3 7 5 8
5 3 3 3 4 9 4 4 7 7 8 4 10 4 −100 11 5 3 8
7 3 6 4 6 6 7 3 3 8 11 4 3 4 −100 4 3 4 5
7

TPB 7 17 3 14 17 9 9 5 5 9 14 17 19 6 13 10 11 −100 8
15 24 15 17 20 −100 11 14 7 4 21 21 13 6 10 −100 −100 6 6
17 8 11 7 15 9 13 12 −100 19 6 18 14 14 6 −100 9 10 22
7 9 8 3 8 12 8 11 19 13 15 11 8 −100 21 15 7 5 15
5 19 14 15 18 7 15 23 15 14 7 6 20 10 13 7 17 13 20
14 5 8 19 10 13 9 14 17 12 20 13 15 7 −100 23 9 8 24
15 6 9 11 8 14 23 10 6 20 21 6 5 6 −100 8 7 9 14
10

TPC 22 18 13 20 19 14 32 21 8 19 34 23 33 23 36 10 14 −100 9
29 32 23 24 14 −100 12 20 32 12 36 43 11 38 19 −100 −100 9 16
13 31 29 17 12 34 15 31 −100 15 16 10 24 28 9 −100 28 19 15
8 10 19 13 24 43 29 14 20 24 19 18 7 −100 27 28 24 28 16
10 17 33 23 21 24 15 14 20 29 16 19 17 18 27 19 14 15 20
24 17 13 22 11 9 19 23 18 8 17 29 22 13 −100 18 24 7 18
7 17 6 30 6 13 14 14 12 15 18 16 10 25 −100 22 7 20 21
12

TPD 13 4 5 9 3 4 9 10 9 4 4 6 4 10 21 3 13 −100 3
7 3 4 23 3 −100 3 4 5 3 6 12 3 14 9 −100 −100 5 13
10 24 14 5 4 12 3 7 −100 3 4 3 4 3 5 −100 14 6 3
6 11 7 17 12 8 14 3 3 11 3 4 3 −100 3 4 3 9 4
8 7 12 3 4 20 3 4 4 7 3 3 8 3 24 9 8 3 3
12 3 17 14 3 3 9 35 3 3 3 3 5 10 −100 3 5 6 3
3 6 4 6 3 3 3 13 8 4 5 4 3 10 −100 4 6 10 3
3

Genotypic and phenotypic data of RIL6013

Linkage map

satt129	1	0
Satt147	1	13.6
Sat_413	1	36.76
Sat_160	1	65.21
satt482	1	70.95
satt254	1	92.57
satt515	1	113.09
AZ302047	1	125.97
satt402	1	152.5
sat_096	2	0
sat_289	2	46.8
staga002	2	49.86
satt546	2	70.38
satt041	2	79.35
satt237	3	0
Sat_166	3	15.15
Satt584	3	21.3
Satt660	3	28.71
satt159	3	36.55
satt631	3	38.68
satt125	3	47.41
Satt713	4	0
satt565	4	23.34
satt396	4	44.85
sat_367	4	51.81
sat_140	4	63.7
satt276	5	0
sat_171	5	30.53
SOYNOD26A	5	49.86
sat_374	5	62
satt174	5	65.43
satt545	5	70.17
satt200	5	79.9
sat_267	5	101.72
satt316	6	0
satt277	6	27.47
sat_246	6	40.13
satt376	6	53.88
satt307	6	64.5
satt202	6	82.85
satt371	6	96.3

satt681	6	112.44
satt281	6	125.91
sat_336	6	138.58
satt640	6	161.64
sat_389	7	0
satt210	7	24.74
satt346	7	32.25
Sat_226	7	58.45
satt536	7	77.1
satt626	7	87.72
satt245	7	100.38
satt378	8	0
satt119	8	19.78
sat_406	8	36.78
satt424	8	58.81
satt233	8	79.99
Sat_087	9	0
Satt167	9	23.74
Sat_243	9	46.65
satt673	9	66.36
Sat_303	10	0
Satt633	10	21.29
satt479	10	31.27
Sat_341	10	53.77
Sat_221	10	76.28
BF008905	10	80.08
Sat_095	11	0
sat_128	11	20.11
satt197	11	30.25
sat_123	11	48.22
BE806308	11	54.37
sat_272	11	59.32
satt583	11	63.87
satt359	11	86.15
satt293	12	0
Satt181	12	10.04
satt434	12	19.68
satt343	13	0
satt030	13	22.57
sat_240	13	36.14
sat_039	13	52.84
Sat_417	13	77.85
satt334	13	105.31
SOYHSP176	13	112.44

satt374	13	139.52
satt425	13	163.67
satt168	14	0
sat_009	14	9.88
satt474	14	11.99
Sat_230	14	27.89
Satt070	14	52.19
satt577	14	86.67
satt231	15	0
satt685	15	23.07
sat_136	15	30.05
satt651	15	47.73
Satt045	15	62.35
Satt483	15	64.23
Satt553	15	94.16
Satt596	16	0
Satt414	16	30.04
Sat_255	16	46.05
sat_394	16	70.92
Sat_366	16	77.2
sat_228	16	83.37
Sct_065	16	115.2
satt154	17	0
Satt669	17	18.82
sat_326	17	40.24
satt186	17	51.99
sat_333	17	67.17
sat_194	17	80.74
sat_001	17	103.16
sat_403	18	0
satt309	18	20.4
sat_210	18	30.54
AW734137	18	45.3
satt570	18	58.3
satt688	18	79.14
Sat_372	18	99.47
sat_117	18	110.49
satt352	18	119.98
satt564	18	125.72
sat_203	18	139.5
satt373	19	0
satt313	19	15.02
sat_191	19	17.61
sat_405	19	29.58

sat_195	19	45.05
satt448	19	58.04
satt182	19	77.95
sat_268	20	0
sat_170	20	27.47
satt330	20	34.02
satt367	20	52
satt571	20	67.53

Marker Types

satt129 A B B A A A A B B A B A A B A B B B
B B A B B B A A A A A A B B B A B B A
A A B A A B A A A A A A A A A A A A A
A B B B A B A A A A A B B A A A B B B
B A B A A A B B A B A B A A B A A A B
A B A B A A B B B A A B A A A A A A A
X A B A A A B A X B A B A A B A A B B
B B B A A B B B B B A B A A A A X X A
A A B X X
Satt147 A A A B B A A X B B A B A A B A B B
A B A A A A A A A A A A B B B A B B A
A A B A A B A A A A A A A A A A A A A
A B B A A B A A A X A A B A A A B B B
X A B B A A B B A B A B A A B A A A B
A A A A A A A A A B A B A A A A A A X
B A B A A A A A A B A B A A B A A B A
B B A A A A X A B B X A A A A A A A X
B A B A B
Sat_413 A B A A A B A A A B A A A A B A B B
A A A B A A A A A A A A B B B A A A A
A B A A A B B A A A A A A B A A A B A
A A A A A A A A A A A A B A B A B B B
B A A B B A B A A B B A A A B B A A A
A B A B B A A A A A A A B B A B A A A
A A A B B A A A A B A B B A B A A B A
B A A A A A B A A B A A A A B B A A A
A A A A B
Sat_160 A A A A A A A A A A A A A A A A A A
A A A A A A A A A A A A A A A A A A A
A A A A A A A A A A A A A A A A A A A
A A A A A A A A A A A A A A A A A A A
A A A A A A A A A A A A A A A A A A B
B A A A A A A A A A A A B A A A B A A

A	A	A	A	A	A	A	B	A	A	B	A	A	A	B	A	A	A	B
A	A	A	B	A	B	A	A	A	A	A	A	A	A	A	A	A	A	A
A	A	A	A	A														
satt482	A	A	A	A	A	A	A	A	A	A	A	B	A	A	A	A	A	A
A	A	A	A	A	A	A	A	A	A	A	A	A	A	A	A	A	A	A
A	A	A	A	A	A	A	A	A	A	A	A	A	A	A	A	A	A	A
A	A	A	A	A	A	A	A	A	A	A	A	A	A	A	A	A	A	A
A	A	A	A	A	A	A	A	A	A	A	A	A	A	A	A	A	A	B
A	A	B	A	A	A	A	A	A	A	A	A	A	A	A	A	A	A	A
A	A	A	A	A	A	A	A	A	A	A	A	A	A	A	A	B	A	B
A	A	A	A	A	A	A	B	B	A	A	B	B	B	A	A	A	A	A
A	A	A	A	A														
satt254	A	A	A	X	A	A	A	A	B	X	A	A	B	A	A	A	A	X
A	A	A	A	B	A	B	A	B	A	A	A	B	B	A	A	X	A	A
A	A	B	B	B	B	A	B	A	A	A	A	A	A	A	A	A	B	A
A	A	A	A	A	B	A	A	A	A	A	B	A	B	A	A	B	A	B
A	A	B	A	A	A	B	A	A	A	A	A	B	A	A	A	A	B	B
A	A	B	A	A	B	A	A	A	A	A	A	A	B	A	A	A	B	A
B	A	A	A	A	A	A	A	B	A	A	A	B	A	A	B	A	B	A
B	A	B	A	A	A	A	A	B	A	A	A	B	A	B	A	A	B	B
A	A	B	B	B														
satt515	A	A	A	B	A	A	A	A	B	B	A	B	B	A	B	A	A	B
X	A	A	B	B	A	A	A	B	A	A	A	B	B	A	A	B	A	A
A	A	A	A	A	A	A	A	A	A	A	B	A	B	B	A	A	A	A
B	A	A	A	A	B	A	A	A	A	A	B	B	B	A	A	B	A	A
B	A	B	A	A	A	B	A	A	A	A	B	A	A	A	B	A	A	B
A	A	A	A	A	A	B	A	A	A	A	X	A	B	A	A	A	A	A
A	A	B	A	A	A	B	B	A	B	A	A	A	B	A	A	A	A	A
A	B	B	B	A	A	A	A	B	A	A	A	B	A	B	A	A	A	A
B	A	A	B	B														
AZ302047	A	A	A	B	A	A	A	A	B	A	A	B	B	A	B	A	A	B
B	A	A	B	B	A	A	A	B	A	A	A	B	B	A	A	B	A	B
B	A	A	A	A	B	A	A	A	A	A	A	A	A	A	A	A	A	A
A	A	A	B	A	B	A	A	B	B	A	A	B	B	A	A	A	A	A
B	A	B	A	A	A	B	B	A	A	A	B	B	A	A	B	A	A	B
A	B	A	A	A	A	A	B	A	A	A	B	A	B	A	A	A	A	A
A	B	B	A	B	A	A	A	A	B	A	B	B	B	B	B	A	A	A
A	B	B	A	A	A	A	A	B	B	B	B	B	A	A	A	A	A	A
B	A	A	B	B														
satt402	A	A	B	B	A	A	A	A	B	B	A	B	B	A	B	A	A	B
B	A	A	B	A	A	A	A	B	A	A	A	B	B	A	A	A	B	A
A	A	A	A	A	A	A	A	A	A	A	A	A	A	A	A	A	B	B
A	B	B	B	B	B	A	A	B	A	A	B	B	B	B	A	B	B	B
B	A	B	B	B	A	B	B	B	A	A	B	B	A	A	B	B	B	B

B B B B B B B B A A A A A A B A A B A

A A B A A A B A B A A B A A A A A A A

A B A A A A A A B A A A B A A A A A B

A A B B A

sat_096 B B B B B B B A A A A B B B B B A B

B A B B B A A A A A A A A A B A B A A

A B B B A A A B B B B A A A A A A A A

A B B A B A A A B A A B A B A A A B A

A B B A A A B A A A A A A B B A A B B

A B A A B B A A A A A A A A B A A A B

A B B A A B A A A B A A B B A A B A B

B A A B A A A B B A A B B A B A A A A

B B A A B

sat_289 A A A A A A A A A A A A A A A A A A

A A A A A A A A A A A A A A A A A A A

A A A A A A A A A A A A A A A A A A A

A A A A A A A A A A A B A A A A A A A

A A A A A A A A A A A A A A A A A A A

A A A A A A B B A A A A A A A A A A A

B A A A B B A A A A A A A A A A A B A

B A A A A A B B A A A A A A A A B A A

A A A A A

staga002 A A A A A A A A A A A A A A A A A A

A A A A A A A A A A A A A A A A A A A

A A A A A A A A A A B A A A B A A A A

A A A A A A A A A A A A A A A A A A A

A A A A A A A A A A A A A A A A A A A

A A A A A A A A A A A A A A A A A A A

B A A A B A A A A A A A A A A B A B A

B A B A A A B B A A A A A A A A A A A

A A A A A

satt546 B B B B A A A A A A A A B A A A A A

A B A A B A A A A A A A A B B A A B A

A B A A A B A A A A X A A A A A A A A

A A A A A A A A A B A A B B A A B A B

B A B A A A B A A A A A A A A A A A A

A B A B A A B B A A X B A A A B A A A

B A A B B A B A B A X B A B B A A B A

B B B B A A B X B B X X B A A A A A B

A B A A B

satt041 A A B B A A A A A A A A B A A A A A

A B A B B B A A A A A X A B B A B B A

A B B A A B A A A A A A A A A A A A A

A A A A A B A A B A A A B A A A A A B

B A A A A X X B A A A A A A A A A A A
A B A B A A B B B A B B A B A B A A A
A A B B B A B A A A A A X B B A A B A
B B A B A A B A B B A A B A A B A A B
A B B A B
satt237 A A A A A A A B A A B A A A A B A A
A A B A B A A A A B A B A A B A A A B
A A B A A A A A A A A B A A B A A A A
A A A B A A A A A A A A A A A A B A X
A B A B A X B A A B B A A B A B A A A
A A A B A A A A A A A A A A A B A A A
A A A A A A A A A A B A A A A A A A A
B B A A A A B A A B B A A B A A A A A
A A A A A
Sat_166 A A A A A A A A A A A B A A A A A A
A A A A A A A A A A A A A A A A A A A
A B A A A A A A A A A A A A A A A A A
A A A A A A A A A A A A A A A A A A A
A A A A A A A A A A A A A A A A A A A
A A A A A A A A A A A A A B A A A A A
A A A A A A B A A A A A A A A A A A A
A A A A A A A A A A A A A A A A A A A
A A B A A
Satt584 A A A A A A A A A A A A A A A A A A
A A A A A A A A A A A A A A A A A A A
A A A A A A A B A A A A A A A A A A A
A A A A A A A A A A A A A A A A A A A
A A A A A A A A A A A A A A A A A B A
A A A A A A B A A A A A A A A A A A A
A A A B A A A A A A A A A A A A A A B
A B A A B A B A A B A B A A A A A B B
A A A A A
Satt660 A A A A A A A A A A A A A A A A A A
A A A A A A A A A A B A A A A A A A A
B A A A A A A A A A B A A B A A A A A
A A A A A A A A A A A A A A A A A A A
A A A A A A A A A A A A A A A A A A A
A A A A A B B A A A A A A A A A A A B
A A A B A A A A A A A A B A B A B A B
A B A B A B A B A A B B A A B A A A B
A A A A A
satt159 A A A A A A A A A A A A A A A A A A
A A A A A A A A A A A A A A B A A A B
A A A A A A A A A A A A A A A A A A A

A	A	A	A	A	A	A	A	A	A	A	A	A	A	A	A	A	A	A
A	A	A	A	A	A	A	A	A	A	A	A	A	A	A	A	A	A	B
A	A	A	A	A	A	B	A	A	A	A	A	A	A	A	A	A	A	A
A	A	A	A	A	A	A	A	A	A	A	A	A	B	A	A	A	A	A
A	A	A	A	A	A	A	B	A	A	B	A	A	A	A	A	A	A	A
A	A	A	A	A														
satt631	A	X	X	X	A	A	A	A	X	X	A	A	X	A	A	A	A	A
A	A	A	X	X	X	A	A	A	A	A	A	X	X	X	A	X	X	X
X	X	A	A	A	A	X	A	A	A	A	A	A	A	A	A	A	X	A
A	X	A	X	A	X	A	X	A	X	A	X	X	A	A	A	X	X	X
X	A	A	A	X	A	X	X	A	A	X	X	X	A	A	A	A	A	X
A	A	A	X	A	A	B	B	A	A	X	X	A	A	A	X	A	A	A
B	A	X	A	B	A	A	A	A	A	A	X	X	B	X	A	A	A	A
B	X	A	X	A	A	A	X	X	X	X	A	X	A	X	X	A	A	X
A	X	X	X	A														
satt125	A	A	B	A	A	A	A	A	B	A	A	A	A	A	A	A	B	A
A	A	A	B	A	B	A	A	B	A	A	A	A	A	B	A	A	B	A
A	B	A	B	A	B	A	A	A	A	X	A	A	A	A	A	A	B	A
A	A	B	A	A	A	A	B	A	B	A	A	B	A	A	A	B	B	B
B	A	A	A	A	A	B	A	A	B	A	B	A	A	B	A	A	X	A
A	A	A	A	A	B	A	A	A	A	A	A	A	A	A	B	A	A	A
B	A	B	A	A	A	A	A	A	B	A	A	A	B	B	A	A	B	A
B	A	B	A	A	A	X	B	B	B	X	A	X	A	B	B	A	A	B
A	B	A	A	A														
Satt713	A	B	A	B	A	A	A	A	A	B	A	B	A	A	A	A	B	B
B	A	A	A	A	A	A	A	B	A	A	B	B	B	B	B	B	B	A
A	B	B	B	A	B	A	A	A	A	A	A	A	A	A	A	A	A	A
A	A	A	B	A	B	B	B	B	B	A	B	B	A	A	A	A	B	A
B	A	A	A	A	A	B	A	A	B	X	A	A	A	B	B	A	A	B
A	B	A	B	A	A	A	A	B	A	A	A	A	B	A	A	A	A	A
X	A	B	B	A	A	B	A	X	A	X	B	A	A	X	A	A	X	A
X	X	X	B	A	A	A	X	B	B	X	A	B	A	B	A	A	A	A
A	B	B	B	B														
satt565	B	A	A	A	A	A	A	A	A	B	A	A	A	A	A	B	A	A
A	A	A	B	A	A	A	A	B	A	A	A	A	B	B	A	A	B	B
A	B	B	A	A	A	A	A	A	A	A	A	A	A	A	A	A	A	A
A	A	A	A	A	A	A	B	A	B	A	A	A	A	A	A	A	A	A
A	A	B	A	B	A	A	A	A	A	A	A	B	A	A	B	A	A	B
A	B	A	A	A	A	B	A	B	A	B	B	A	B	A	B	A	A	A
A	A	B	A	B	A	A	A	A	B	B	B	A	B	B	A	B	B	A
B	A	B	B	A	A	B	B	B	A	A	A	B	A	B	B	A	A	A
A	B	B	B	B														
satt396	A	A	A	A	A	A	A	A	A	B	A	B	B	A	A	A	A	A
A	A	A	B	B	B	A	A	B	A	A	A	A	A	A	A	A	B	A

A A A B A A A A A A A A A A A A A B A
A B A A A A A B A A A A A B A A A B A
A A B A B A A A A A A B B A A B A B A
A A A A A A A A A A A B A A A A A A A
A A A B A A A A A A A B A B B A A A A
A B B A A A A A B B A A A A B A A A A
A A A A A
sat_367 A B A A A A A A A B A B B A A A A A
X A A A A A A A B A A A A A A A A B A
A A A B A A A A A A B A A A A A A A X
B X A A A A A A B A A A A B A A A B A
A A A A B A A A A A B B A A B A A B A
A A A A A A A B A A A B A X A A A A A
A A A B B A A A A B X B A B B A A X A
A B B A A A A A B B A A A A B A A A X
A X B A A
sat_140 A A B B A A A A A B A B B A B B A A
A A A A B A A A B A A A A A A A A B A
A A A A A A A A A A B A A B A A A A A
A A A A A A A A B A A A B A A A B A A
A A A A B A A A A A B A B A B A A A A
A B A A A A A A A A A A A A A B A A A
A A A B A A A A A A A A B B B A A A A
A B A A A A B B B B A A A A B A A A A
A A A A A
satt276 A A A A A A A A A B A B B A B A A A
A B A B B B B A A A A A B B B A B B A
A A B A A A B A A A A A A B A A A B A
B B B B A B A B B A B B B B B A A A B
B B B B B A A B B A B A B A A A B A B
B B A A B A B A A A A A A A A B B B A
B B A B A A B A A A B B B B B A A B A
A B A B A B B A B A B A B A B A A A B
B A A B B
sat_171 A A A A A A A A B B A A A A B A B B
B B B B A B B A A A A A B B A A A B A
A A B A A B B A A A A A A B A A A A A
A A B B A A A A B A B B A A A A A A A
A A B B B B A A A B B B B A A B A A B
B A A B A B A B B A A A A B A A A A A
A A A A A A A A A A A B B B B A A B A
B A A B A A B B B A B B A A B B A A A
B A B A B
SOYNOD26AA A A A A A A A A A A B B A B A B B

A	B	A	B	A	B	A	B	A	A	A	A	A	A	A	A	A	A	B
A	A	B	A	A	A	A	A	B	A	B	A	A	A	A	A	A	A	A
A	A	A	A	A	A	A	A	B	A	A	B	A	B	A	A	A	A	A
A	A	B	A	A	A	A	A	A	A	B	A	A	A	A	B	A	A	B
A	A	A	A	A	A	A	B	A	A	A	A	A	B	A	A	A	A	A
A	A	A	A	A	A	A	A	A	A	A	A	B	B	A	A	A	A	B
A	A	A	A	A	A	B	A	B	A	B	A	B	A	A	A	A	A	A
B	A	B	A	B														
sat_374	A	A	A	B	A	A	A	A	A	A	A	A	A	A	B	A	A	B
B	A	A	A	A	A	A	A	A	A	A	A	A	A	B	A	A	A	B
A	A	A	A	A	A	A	A	A	A	A	A	A	A	A	A	A	A	A
A	A	A	A	A	A	A	A	A	A	A	A	A	A	A	A	A	A	A
A	A	A	A	A	A	A	A	A	A	A	A	A	A	A	A	A	A	B
A	A	A	A	A	A	B	A	A	A	A	A	A	A	A	A	A	A	A
A	A	A	A	A	A	A	A	A	A	A	A	A	B	A	A	A	A	A
A	A	A	A	A	A	A	A	A	A	B	B	B	A	A	A	A	A	A
A	A	A	A	A														
satt174	A	A	A	A	A	A	A	A	A	A	A	A	A	A	A	A	A	A
A	A	A	A	A	A	A	A	A	A	A	A	A	A	B	A	A	A	A
A	A	A	A	A	A	A	A	A	A	A	B	A	A	B	A	A	A	A
A	A	A	A	A	A	A	A	A	A	A	A	A	A	A	A	A	A	A
A	A	A	A	A	A	A	A	A	A	A	A	A	A	A	A	A	A	B
A	A	A	A	A	A	B	A	A	A	A	A	A	A	A	A	A	A	A
A	A	A	A	A	A	A	A	A	A	A	A	A	B	A	A	A	A	A
A	A	A	A	A	A	A	A	A	A	B	A	B	A	A	A	A	A	A
A	A	B	A	B														
satt545	A	A	A	A	A	A	A	A	A	A	A	B	A	A	A	A	A	A
A	A	B	A	B	A	A	A	A	A	A	A	X	X	A	A	A	X	A
A	A	A	A	A	A	X	A	A	A	A	A	A	A	A	A	A	A	A
A	A	A	A	A	A	A	A	X	A	X	X	X	A	A	A	A	X	A
A	A	A	A	X	A	A	A	A	A	A	X	X	A	A	A	A	X	A
A	A	A	A	A	X	B	A	A	A	A	A	A	A	A	A	A	A	A
A	A	A	A	A	A	A	A	A	A	A	A	A	B	A	A	A	A	A
A	A	A	X	B	A	B	A	X	A	A	A	A	A	A	A	A	A	A
A	A	X	A	A														
satt200	B	B	B	A	B	B	B	B	B	A	B	B	B	B	A	B	B	A
A	B	B	B	B	B	A	A	A	A	A	A	X	X	X	A	X	A	A
A	A	X	A	A	A	X	A	A	A	A	A	A	X	A	A	A	A	A
A	A	A	A	A	X	A	A	A	A	A	A	A	A	A	A	A	X	A
A	A	X	A	X	A	A	A	A	A	X	A	X	A	A	A	A	A	B
A	X	A	A	A	A	B	X	A	A	A	A	A	A	A	X	A	A	A
A	A	A	A	A	A	X	A	A	A	A	A	X	B	X	A	A	A	A
A	A	A	A	A	A	A	A	X	A	A	A	A	A	X	A	A	A	A
X	A	B	B	A														

sat_267	A	A	A	B	B	A	B	B	B	A	A	A	A	A	B	A	A	B
B	B	A	B	B	A	A	A	A	A	A	A	B	B	A	A	B	B	A
B	B	A	B	A	A	A	B	A	A	B	B	A	B	A	A	A	A	A
A	A	B	A	A	A	A	A	B	A	A	B	A	B	A	A	A	A	A
A	A	B	A	B	A	A	A	A	A	A	A	B	A	A	A	A	A	A
A	B	A	A	B	B	A	A	B	A	B	B	A	A	A	A	A	A	A
A	A	A	A	A	A	A	A	A	A	A	B	A	B	A	A	A	A	A
A	A	A	A	A	A	B	A	A	A	B	A	A	A	B	A	A	A	A
B	A	A	A	A														
satt316	A	B	A	B	A	A	A	A	B	B	A	B	B	A	A	A	A	B
A	B	A	B	B	B	A	A	A	A	A	A	B	B	A	A	B	B	A
B	A	B	B	A	B	A	A	A	A	A	A	A	A	B	A	A	B	A
A	B	B	B	B	A	A	A	B	A	A	B	B	B	A	A	A	A	B
A	B	B	A	B	A	A	B	A	A	B	B	B	A	A	B	B	B	B
A	A	A	A	A	B	A	B	B	A	A	B	A	B	A	B	A	A	A
A	B	B	B	A	A	B	B	A	A	A	A	A	A	B	A	A	B	A
A	A	A	B	A	A	B	B	B	A	A	B	B	A	A	A	B	A	B
A	B	A	A	B														
satt277	A	A	A	B	A	A	A	A	B	B	A	A	A	A	A	A	A	A
A	A	A	A	A	A	A	A	A	A	A	A	B	B	B	A	B	A	A
A	A	A	A	A	A	A	A	A	A	A	A	A	A	A	A	A	B	A
A	B	A	B	A	B	A	A	B	A	A	B	A	A	A	A	A	B	A
A	A	A	A	A	A	A	A	A	A	B	A	A	A	A	A	A	A	B
A	A	A	B	A	A	A	A	A	A	A	A	A	A	A	A	B	A	A
A	A	A	A	A	A	A	A	A	A	A	B	B	A	B	A	A	A	A
A	A	A	B	A	A	A	A	B	A	A	A	A	A	A	A	A	A	B
A	B	A	A	B														
sat_246	A	A	A	A	A	A	A	A	A	B	A	A	A	A	A	A	B	A
A	B	A	A	A	A	A	A	B	A	A	A	B	B	A	A	A	A	A
A	B	A	B	A	A	A	A	A	A	A	A	A	A	A	A	A	B	B
A	A	B	B	A	B	A	A	B	B	A	B	A	B	A	A	A	A	B
B	A	A	A	B	A	A	A	A	B	B	B	A	A	A	A	A	A	B
A	A	A	A	A	A	A	A	A	A	A	A	A	A	A	A	A	A	A
A	A	B	A	A	A	A	A	A	A	A	A	A	B	A	A	A	A	A
A	A	A	A	A	A	A	A	A	A	A	A	A	A	A	A	A	A	A
A	A	A	A	B														
satt376	A	A	A	A	A	A	A	A	A	B	A	A	A	A	A	A	A	A
A	A	A	A	A	A	A	A	A	A	A	A	A	A	A	A	A	A	A
A	A	A	A	A	A	A	A	A	A	A	A	A	A	B	A	A	A	A
A	A	A	A	A	A	A	A	B	B	A	B	A	B	A	A	A	A	A
B	B	A	B	A	A	A	A	A	B	B	A	A	A	A	A	A	A	A
A	A	A	A	A	A	B	A	A	A	A	B	A	A	A	A	A	A	A
B	A	A	B	A	A	A	A	A	B	B	A	B	A	A	A	A	A	A
B	A	A	B	A	A	B	A	A	A	A	A	A	A	B	A	A	A	A

A B A A B

satt307 A A B A A A A A A A A A A A A A A A

A A A A A A A A A A A A A A A A A A A

A A B A A A A A A A A A A A B A A A A

A A A B A A A A A A A A A A A B A A A

B A B A B A A A A A B A A A A A B A A

A A A A A A A A A A A B A A A A A A A

A A A B A A A A A A A A A A A A A A A

A A B B A A B B A A A A A A A A A A B

A A A A A

satt202 A A A A A A A A B B A A A A B A A B

B A A B A B A A B A A A B B A A A B A

A A A A A A A A A A A A A A A A A A A

A A A A A A A A A X A A A A A A A A B

A A A B A A B A A A A A X A A A A A A

B A A A B B A B A A A B A B A B A A A

A A B B A A B A B A A B A A B A A A A

A A X B A A X X A A A A B A A X A A B

B B A A B

satt371 A A B A A A A A B B A A A A B A A B

B B A A A B A A B A A A A A A A A B A

A A A B A X A A A A A A A A A A A B A

A A B A A A A A B B A A B B A A B B B

A A A A A A A B A A A B A A A B A A B

A A A A A B A A A A A B A B A B A A A

A A B B A A B A B B A B A A A B B A A

B A B A A A B A A B A A A A A A A B B

B B A A B

satt681 X A A B A A A A A B A A A A B A B B

B A A B A B A A A A A A X B B A B B A

A A A B A B A A A A A A A A A A A A X

A B A A A B A A A B A A B A A A A A B

A A A A A A A B A B A A A A A B A A X

A X A A A A A A A A A X A B A A A A X

X A A A B A B A B B A B A B A X A B A

B A X A A A A B A B A X B A A B A A X

B B B A B

satt281 A A B B A A A A B A A A B A B A B A

A A A A B B A A A A A A A A A A B A B

A A B A A B A A A A A A A A A A A A A

A B A A A B A B A B A B B A A A B A A

A A B A A A A B A B B A A A A B A A A

A B A A A A A B A A A B A B A A A A A

B A B A A A A A A B A B A B A A A B A

B B B A A B A B A B A A B A A A A A B
B B B B A
sat_336 A B B B A A A A B B A A B A A A B A
A A A A B A A B A A A A B B B A B A B
A A B B A B A A A A A A A A B A A B A
A B B B A B A B A B A B B A A A B A A
B B B A A A A B A B B A A A A B A A B
A B A A A A A A A A A A A B A A A A A
A A B B B A A A A A A B A B B B A B A
A B B A A A B B B B A A B A A A A A A
B B B A B
satt640 A B A B A A A A A A A A B A B A B A
A B A A B A A B A A A A A A A A B A A
A A B A A B A A A A A A A B A A B A A
A B B A A B A B A B A A A A A A A B B
B A A A A A A B A B A A B A A B A A A
A B B A A A A B A A A A B B A A A B A
A A B A B A B A B A B A A A B B A A A
A B B A B A A B B B B B B A B A A B B
A A A A A
sat_389 B A A A A A A A A A A A A A B A A B
B A A B A B A A B A A A A A A A B B A
A B A B A A A A A A A A A A A A A B A
A B A B A B A B A A A B A A A A A A B
B A A A A A B A A A A A A B A A A A A
A B A B A A A A A A B B A B A B A A A
B A A A A A B A A B A B A A A B A A B
B A B A A A B B A B A A B A A B A A B
B A B A A
satt210 A A B B A A A A A B A B A A B A B A
B A A B A A A A A A A A B B A A B A A
A A B A A A A A A A A A A A A A A A A
A A B A A B A A B B A B B A A A A A A
A B B A A A B A A B B A A A B B A A B
A A A A A A A B A A A B A B A B A A A
A A B A A A B A A B A B A A A B A A A
A A A A A A A B B A A A B A B A A A B
A B B A A
satt346 A A B B A A A A A B A B A A A A B A
A A A B A A A A A A A A B B A A A A B
A A B A A A A A A A A A A A A A A A A
A A B A A B A A B B A B B A A A B B B
B A A A A A B A A B B A A A B B A A B
A A A A A A A B A A A B A A A A A A A

A A A B A A B A A B A B A A B A A B A
A B B A A A X B B A X A B A B A A A B
A B B A B
Sat_226 A B B B B A A A A B A A A A B A B A
A B A A B A A A A A B A A A A A B A B
A B B A A B A A A B A B A B A A A A A
A A A B B A A B B B A B B A A A A B A
B A B A B A B B A B B A B A B B A A A
A A A B A A A A B B A B B A B A A B A
A A B A B B B A B A B A B A B B A B A
B B B B A A A B B A A A B A B B A A B
B B B A A
satt536 A B A B A A A A B A B A A B B A B A
A B A A B A A A A A A A A A A A B A B
A B B A A B A A A A A A A A A A A A A
A A B B A A B B A B B B A A A A B B A
B A B A B A B B A B B A A A B B A A A
A B A B A A A A B A B A A B A B A A A
B B A B A B B B B A B A B B B A A B A
B B B B A A B B A A A A B A B A A A A
B B B B B
satt626 A A A B A A A A B A A A B A B A B A
A B A A B A A A A A A A A A A A B A A
A A B A A B A A A A B A A A A A A A A
A A B B B B A A A A A B B A A A B B A
B A B A B A B B A B B A A A B B A A A
A B A B A A A A B A B A A B A B B A A
A A B B B A B A A A B A B B B A A B B
B B B B A A A A A A A A A A A A A A A
B B B B B
satt245 A A B A A A A A B A B A B A B A A A
A B A A B A A A A A A B B A A A B A A
A B A A A B A A A A A B A B A A A A A
A A B A A B A B A A B B B A A A B B A
B A B A B A B B A B A A A A B A A A A
A B A A A A A B A A A A A A A B A A A
A A B B B A B A B A A A B X B A A B A
A B X B A X X A A A A A A A A A A A A
A X A B A
satt378 A A B A A A A A B A A A B A A A B A
A A A A B A A A B A A A A A A A A A A
A A A A A B B A A A A A A B A A A B A
A A B A A B A B A B A A A A A A A B A
A A A A B A A B A B B B B A A B A A A

A	B	A	A	A	A	A	A	A	A	A	B	A	B	A	A	A	B	A
A	B	B	B	A	B	B	A	A	A	A	A	B	B	A	A	A	B	A
A	A	A	A	A	B	A	A	B	B	A	A	B	A	B	A	A	A	A
A	B	A	A	B														
satt119	A	A	B	A	A	A	A	A	B	A	A	A	A	A	B	A	A	B
B	A	A	A	A	A	A	A	A	A	A	A	A	A	A	A	B	A	X
A	A	B	A	A	X	A	A	A	A	A	A	A	A	A	A	A	A	A
A	A	B	A	A	B	A	B	B	A	A	A	B	A	A	A	B	A	B
X	A	A	A	A	A	B	A	A	A	A	A	A	A	B	B	A	A	A
A	B	A	A	A	A	B	A	A	A	A	A	A	B	A	A	A	A	A
A	A	A	B	A	A	A	A	A	B	A	A	A	B	A	A	A	B	A
A	A	A	B	A	A	A	X	A	B	A	A	X	A	B	A	A	A	X
B	B	B	A	A														
sat_406	A	A	A	A	A	A	A	A	A	A	A	A	A	A	A	A	A	A
A	A	A	A	A	A	A	A	A	A	A	A	A	A	A	A	A	A	A
A	A	A	A	A	A	A	A	A	A	A	A	A	A	A	A	A	A	A
A	A	A	A	A	A	A	A	A	A	A	A	A	A	A	A	A	B	A
A	A	A	A	A	A	A	A	A	B	A	A	A	A	A	A	A	A	B
A	A	A	A	A	A	A	B	A	A	A	A	A	A	A	B	A	A	A
A	A	A	A	A	A	A	B	A	B	A	A	A	A	A	B	A	A	B
A	A	A	B	A	A	A	B	B	B	A	A	A	A	A	A	A	A	B
A	A	A	A	B														
satt424	A	A	A	B	A	A	A	A	B	B	A	A	B	A	A	A	B	B
B	B	A	A	B	A	A	A	A	A	A	A	B	B	A	A	A	A	A
A	B	B	A	A	B	A	A	A	A	A	A	A	A	A	A	A	B	A
A	B	A	B	A	A	A	A	B	B	A	B	B	A	A	A	A	X	A
X	A	B	A	A	A	B	B	A	B	A	B	A	A	A	B	A	A	X
A	B	A	A	A	A	B	B	A	A	A	B	A	X	A	B	A	X	A
A	A	A	B	X	A	B	A	X	A	A	B	A	A	B	X	A	A	A
X	B	B	B	A	A	A	X	A	B	A	X	X	A	A	X	A	X	X
X	B	B	A	B														
satt233	A	A	A	B	A	A	A	A	B	A	A	B	A	A	A	A	A	A
A	A	A	B	A	B	A	A	B	A	A	A	B	B	A	A	A	B	A
A	A	B	B	A	A	B	A	A	A	B	A	A	B	A	A	A	B	A
A	B	B	B	A	A	A	A	B	B	A	A	B	B	A	A	A	B	B
B	A	B	A	B	A	B	A	A	A	B	B	B	A	B	B	A	A	A
A	B	A	A	A	B	A	B	A	A	A	B	A	B	A	B	A	A	A
B	A	B	B	B	A	B	A	B	B	A	B	B	B	B	A	A	B	A
B	A	A	B	A	A	B	B	B	B	A	A	A	A	A	B	A	A	B
A	B	B	A	B														
Sat_087	A	A	B	A	A	A	A	A	B	A	A	B	A	B	A	A	A	A
A	B	A	B	A	X	A	A	B	A	A	A	A	A	A	A	A	A	A
A	A	A	B	A	A	B	A	A	A	A	A	A	B	A	A	A	B	B
A	B	A	A	A	A	A	B	A	A	A	B	A	A	B	B	B	B	B

B A B A B A B A A A A B B A A A A A X
A B A B A B B B A A A A A A A A A B A
A A B B A A X B B B A A B A X A A B A
A A A B A A A A B B A A B X A B A A B
A A A B A
Satt167 A A B X A B B A A B A A A X A A B A
X X A A A A A A B A A A X B A A A X A
X A B B X A A A A A A A A A A A A B B
B A A X A A A A A A A A A A A A B B A
B A B A A A B A A A A B A A A B A A A
A B A B A A A A X A X A A B A B A A A
A A B X A B A A A A A A A B A A A B A
B A A A A X A A B A A A A A B A A A A
B A X A A
Sat_243 B B B B B B B B A A B B B B B B B A
B A B B B B B B A B B B A A A B A B B
B B B A B A A B B B B B B A B B B B B
B A A B B A B B A B B B A B B B B A B
X B A B B B A A B A A B B A B B B B A
B B B A B B B B B B B B B B B B B B B
B B B B B B B B B A B B A B B B B A B
B B A A B B B A B B B B B B A B B B B
X B B B A
satt673 A A A A A A A A B A A B A A A A A A
A A A A A A A A A A A A A A A A A A A
A A A A A A A A A A A A A A B A A A A
A A A A A A A A A A A A A A A A A A A
A A A A A A A B A B A B B A A B B A A
A A A A A A A A A A A A A A A A A A A
B A A B B A A A A A A A A A A B A B A
B A B B A A B B A A A A A A A A A B B
A A A A A
Sat_303 A A B A A A A B X B A A A A B A B B
B B A B A B A A A A A A B B B A A B A
A B B A A B A A A A A A A A A A A A A
A A A A A A A B A B B A B A A B A A B
B A B A A A B A A B A B A A B A A B X
A B A B A A A A B A B A A A A B A A A
A A A A A A B A A B A B A A B A A A A
A A B B A A A A A B A A B A A B A A B
B A A B B
Satt633 B A B A A A A A B B A A B B B B B B
A A A B A B A A A A A A A A A A A B A
X A A A A A A A A A A A A A A A A A B

A	A	B	B	A	A	A	B	A	B	A	X	A	A	B	A	A	A	B
A	A	B	A	A	A	A	B	A	A	A	B	A	A	B	B	A	A	B
A	B	A	B	A	A	A	B	B	A	X	B	A	B	A	A	A	B	B
B	A	A	A	A	A	B	A	A	B	A	B	A	A	A	A	A	B	A
B	A	A	B	A	X	A	B	B	B	A	A	A	A	A	B	A	A	A
B	B	A	B	A														
satt479	A	A	B	A	A	A	A	A	B	B	A	A	A	A	B	A	A	A
A	A	A	B	A	B	A	A	A	A	A	A	A	A	A	A	B	B	A
A	B	A	A	A	A	A	A	A	A	A	A	A	A	A	A	A	A	A
A	A	B	B	A	B	A	B	A	B	A	B	A	B	A	A	B	A	A
A	A	B	A	A	B	A	B	A	A	A	B	A	A	B	B	A	A	B
A	B	A	B	A	A	A	A	B	A	A	B	A	B	A	B	A	A	A
B	A	B	A	A	A	B	A	A	A	A	A	A	A	A	A	A	A	A
B	A	A	B	A	B	A	B	B	B	A	A	A	A	A	B	A	A	A
A	B	B	B	A														
Sat_341	A	A	A	B	A	A	A	A	B	B	A	A	B	A	B	A	A	A
A	B	A	A	A	A	A	A	B	A	A	A	B	B	A	A	B	A	B
A	A	B	B	A	A	A	A	A	A	A	A	A	A	A	A	A	B	A
A	B	A	B	A	B	A	A	B	B	A	B	A	A	A	A	B	B	A
B	A	B	A	A	A	B	A	A	B	X	B	A	A	A	B	A	A	A
A	B	B	B	A	A	A	A	A	A	A	A	A	B	A	B	A	A	A
B	A	B	A	B	A	A	A	A	A	A	A	A	B	A	A	A	B	B
B	A	A	A	A	B	A	B	A	A	A	A	A	B	A	A	A	A	A
B	A	A	A	B														
Sat_221	A	A	A	A	A	A	A	A	A	A	A	A	A	A	A	A	A	A
A	A	A	A	A	A	A	A	A	A	A	A	A	A	A	A	A	A	A
A	A	A	A	A	A	A	A	A	A	A	A	A	A	A	A	A	A	A
A	A	A	A	A	A	A	A	A	A	A	A	A	A	A	A	A	A	A
A	A	A	A	A	A	A	A	A	A	A	A	A	A	A	A	A	A	A
A	A	A	A	A	A	A	A	A	A	A	A	A	A	A	A	A	A	A
B	A	A	B	A	A	A	A	A	A	A	A	A	A	A	A	B	A	B
A	A	A	A	B	A	A	A	A	A	A	A	A	A	A	A	A	A	A
A	A	A	A	A														
BF008905	A	A	A	A	A	A	A	A	A	A	A	B	A	A	A	A	A	A
A	A	A	A	A	A	A	A	A	A	A	A	A	A	A	A	A	A	A
A	A	A	A	A	A	A	A	A	A	B	A	A	A	A	A	A	A	A
A	A	A	A	A	A	A	A	A	A	A	A	A	A	A	A	A	A	A
A	A	A	A	A	A	A	A	A	A	A	A	A	A	A	A	A	A	B
A	A	A	A	A	A	A	A	A	A	A	A	A	A	A	A	A	A	A
A	A	A	B	A	A	A	A	A	A	A	A	A	B	A	A	A	A	A
A	A	A	A	A	A	A	A	A	A	B	A	A	A	A	A	A	A	B
A	A	B	A	A														
Sat_095	B	B	A	A	B	B	B	B	A	B	B	A	B	X	A	A	B	A
B	A	A	A	B	A	B	B	B	B	B	B	B	B	A	B	B	B	B

B	A	B	A	B	A	A	B	B	B	B	B	B	A	B	B	B	X	B
B	A	B	B	B	B	B	A	A	B	B	B	B	B	B	B	A	A	B
B	A	A	B	A	B	A	A	B	B	A	B	A	B	B	A	A	B	B
X	B	B	B	A	A	X	B	B	B	A	B	B	B	B	A	B	B	B
B	B	B	B	B	B	A	B	B	A	B	B	A	B	A	B	B	B	B
B	B	B	A	B	B	B	B	A	B	B	B	A	A	A	A	B	B	B
A	B	A	A	B														
sat_128	A	A	A	B	A	A	A	A	B	A	A	B	A	A	A	A	B	B
A	B	A	A	A	B	A	A	B	A	A	A	B	B	B	B	A	B	B
A	B	A	B	A	B	B	A	A	A	A	A	A	B	A	A	A	B	A
A	B	A	A	A	A	A	B	A	B	A	A	A	B	B	A	A	B	A
A	A	B	A	B	A	B	B	A	B	B	A	B	A	A	B	A	A	A
A	A	A	A	A	A	A	A	A	A	B	A	B	A	A	B	A	A	A
A	A	B	A	B	B	B	A	B	A	A	A	A	A	A	A	A	B	B
B	B	B	B	A	A	A	A	B	A	A	A	A	A	A	A	A	A	A
A	B	A	B	A														
satt197	A	A	A	B	A	A	A	A	B	A	A	A	A	A	A	A	B	B
B	B	A	A	A	A	A	A	B	A	A	A	A	B	A	A	A	A	A
A	B	A	B	A	B	B	A	A	A	A	A	A	B	A	A	A	A	A
A	B	A	A	A	A	A	A	A	B	A	A	A	B	A	A	A	B	A
A	A	B	A	A	A	A	B	A	B	B	A	B	A	A	B	A	A	A
A	A	A	A	A	A	A	A	A	A	A	B	A	B	A	B	A	A	A
A	A	B	A	A	A	B	A	A	A	A	A	B	A	A	A	A	B	A
A	A	B	B	A	A	B	A	B	A	A	A	A	A	A	A	A	A	A
A	B	A	B	B														
sat_123	A	A	A	A	A	A	A	A	A	A	A	A	A	A	A	A	A	A
A	A	A	A	A	A	A	A	A	A	A	A	A	A	A	A	A	A	A
A	A	A	A	A	A	A	A	A	A	A	A	A	A	A	A	A	A	A
A	A	A	A	A	A	A	A	A	A	A	A	A	A	A	A	A	A	A
A	A	A	A	A	A	A	A	A	A	A	A	A	A	A	A	A	A	A
A	A	A	B	A	A	A	A	A	A	A	A	B	A	A	A	A	A	A
A	A	A	A	A	A	A	A	B	A	A	A	A	A	A	A	B	A	A
A	A	A	A	A	A	A	A	A	A	A	A	A	A	A	A	A	A	A
A	A	A	B	A														
BE806308	A	A	A	A	A	A	A	A	A	A	A	B	A	A	B	A	A	A
A	A	A	A	A	A	A	A	A	A	A	A	A	A	A	A	A	A	A
A	A	A	A	A	A	A	A	A	A	B	A	A	A	A	A	A	A	A
A	A	A	A	A	A	A	A	A	A	A	A	A	A	A	A	A	A	A
A	A	A	A	A	A	A	A	A	A	A	A	A	A	A	A	A	A	B
A	A	A	A	A	A	B	A	A	A	A	B	A	A	A	A	A	A	A
A	A	A	B	A	A	A	A	A	A	A	A	A	A	A	B	A	A	A
A	A	B	A	A	A	A	A	A	A	B	A	B	A	A	A	A	A	A
A	B	A	A	A														
sat_272	A	A	A	A	A	A	A	A	A	A	A	B	A	A	A	A	A	A

A	A	A	A	A	A	A	A	A	A	A	A	A	A	A	A	A	A	B
A	A	B	A	A	A	A	A	A	A	A	A	A	A	A	A	A	A	A
A	A	A	A	A	A	A	A	A	A	A	A	A	A	A	A	A	A	A
A	A	A	A	A	A	A	A	A	A	A	A	A	A	A	A	A	A	A
A	A	A	A	A	A	B	A	A	A	A	A	A	A	A	A	A	A	A
A	A	A	A	B	A	A	A	A	A	A	A	A	A	A	A	A	B	A
A	A	A	A	A	A	B	A	A	A	B	A	A	A	A	A	A	A	A
A	A	A	A	A														
satt583	A	A	A	A	A	A	A	A	A	A	A	A	A	A	A	A	A	A
A	A	A	A	A	A	A	A	A	A	A	A	A	A	A	A	A	A	B
A	A	B	A	A	A	A	A	A	A	A	A	A	A	A	A	A	A	A
A	A	A	A	A	A	A	A	A	A	A	A	A	A	A	A	A	A	A
A	A	A	A	A	A	A	A	A	A	A	A	A	A	A	A	A	A	A
A	A	B	A	A	A	B	B	A	A	A	A	A	A	A	A	A	A	A
A	A	A	A	A	A	A	A	A	A	A	B	A	B	B	A	A	A	A
A	A	A	B	B	A	A	A	B	A	A	A	A	A	A	A	A	A	A
A	A	A	A	A														
satt359	B	A	A	B	A	A	A	A	A	A	A	A	B	A	A	A	A	B
B	B	A	A	B	B	A	A	A	A	A	A	B	A	A	A	B	B	B
A	B	B	A	A	A	A	A	A	A	B	A	A	A	B	A	A	B	A
A	A	B	A	A	B	A	A	B	A	A	B	A	A	A	A	B	B	B
A	A	A	A	B	A	B	B	A	A	A	A	A	A	A	A	A	A	A
A	B	A	A	A	B	A	A	A	A	A	B	A	A	A	B	A	A	A
A	A	A	B	B	A	A	A	B	B	A	A	A	A	B	B	A	B	A
A	B	A	B	A	A	B	A	B	B	A	A	A	A	B	B	A	A	A
A	A	A	A	A														
satt293	A	A	A	B	A	A	A	A	B	B	A	B	B	A	A	A	A	B
A	B	A	A	A	A	A	A	A	A	A	A	B	B	A	A	A	A	A
A	A	B	A	B	A	A	B	A	B	A	A	A	B	A	A	A	A	A
A	A	B	B	A	B	A	B	B	A	A	A	B	A	A	A	A	B	A
A	A	A	A	A	A	A	A	A	B	A	B	B	A	A	B	A	A	B
A	A	A	A	A	A	A	A	A	A	A	B	A	B	A	B	A	A	A
A	A	A	A	B	A	B	A	B	A	A	A	B	A	A	A	A	B	A
A	A	B	A	A	A	B	A	A	A	B	A	A	A	B	A	B	A	A
A	A	B	B	B														
Satt181	X	X	X	X	X	X	X	X	X	X	X	X	X	X	X	X	X	X
A	A	A	A	A	A	A	A	X	A	A	A	B	B	B	A	A	A	A
A	A	X	A	A	A	B	A	A	A	A	X	A	A	A	A	A	A	A
A	A	B	B	X	X	X	B	X	A	A	B	A	A	X	A	A	B	A
B	A	A	A	B	A	B	B	A	A	A	X	B	A	A	B	A	A	B
A	B	A	B	A	A	A	A	A	A	A	B	A	B	A	B	A	A	A
A	A	A	A	X	A	B	A	B	A	A	B	B	A	A	A	A	B	X
A	A	B	A	A	A	A	A	A	B	A	A	A	A	B	A	A	A	A
A	A	B	B	B														

satt434 A A A A A A A A B A A A A A A A A A
A A B A A A A A A A A A A A A A B A A
A A B A A A B A A A A A A B A A A A A
A A A B A B A B B B A B A B A A A B B
B A A A B A B A A A A B B A B B A A A
A B A B A A A A B A A B A B A B A A A
A A B A A A B A X A A A B A A B A B A
A A B A A A X B A B A A A A B A A A A
A B B B A
satt343 A A A B A A A A A A A B B A B A B A
A A A A B A A A A A A A A A A A A A A
A B A B A B A A A A B B A A A A A A A
A A A B A B A B B A A B B A B B A B B
A B B A B B B A A B B B B A A B A A A
A B B B B B A B A A A A A A A A A A A
A A A A A A A A A A A A A A A B A A A
A A A A A A A A A A A A A B A A A A A
A A B A A
satt030 A A A X A A A X A A A A B A A A A A
A A A A B A A A A A A A A A A A A B A
A A A A A A B A A A B B A B A A A A A
A A A A A B A A A A A X X B A A B B A
B A B A B A A B A A B A A A A B A A B
A B A A A A A A A A A A A A A A A A A
A A B X B A A A A B A A A A B B A B A
X A A A A A B A B A A A B A X A A A B
B B B X B
sat_240 A A A A A A A A A A A A A A A A A A
A A A A A A A A A A A A A A A A A A A
A A A A A A A A A A B B A A B A A A A
A A A A A A A A A A A A A A A A A A A
A A A A A A A A A A A A A A A A A A A
A A A A A A A A A A A A A A A A A A A
B A A B B A A A A A A A A A A B A A A
A A A A A A A A A A A A B A A B A B B
A A A A A
sat_039 A A A A A A A A B A A A A A A A A B
B B A A A A A A A A A A A A A A B A A
A B A A A A A A A A A A A A A A A A A
A B A B A B A B A B A B A B A A B A A
A A A A A A A A A A A A A A B B A A A
A B A A A A A A A A A A A B A A A A A
B B B B A A A A A A A B A A B A A A A
B A B B A A A B B A A A A A B A A A A

A B A A A
Sat_417 B B B A B B B B A A B B B B B B B A
A A B A B A B B A B B B A A A B A A A
B B A A B B A B B B B B B A A B B B A
B A B A B A B A A A A A B B B B B A A
B B B B B A B A B B B A A B A A B B A
B A B B B B B B A B A B B A B A B B B
B B A B A A A B A A A A A B B X B A B
A B B A B B B A B A B B B A B B B B B
B A A B B
satt334 A B A B A A A B A A A A A A B A A B
B B B A B A A A B A A A A A B A B A B
A B X B A B B A B B A A A B B A A B B
A B A A A B X B A B A A B B A A A B A
A A B A B A B A A A A B B A A B A A B
A A A A A A B B A A B B A B A B A A A
A A A B B A A A B B A A B A A A A B A
B A A A A A A A B A A A B A A A A A B
B A B B A
SOYHSP176 A B A A A A X X A B A A A A B A A A
X X X A B A A A B A A X X X X A A A X
A B B B A X X X X X X X A B B A A X X
A B B A A A B B A B B B B X X A A B A
A A B A B B B A A A A B B X X B B A B
A A A A A A B B A A B A A B A B A A A
A A A B B A A A A B A A B A A A A X A
A A A A A A A A B A A A X A A A A A A
B A A A A
satt374 A B B A A A A A A B A A A A B A A A
A B B B A A A A B A B A B B B A A A B
A B B B A B B B B B B A A B A B B A B
B B A A A A A A A A A B B A B B B A A
A A B A A A A A A A A B A A B B B A A
B B A A A A A B B A A A A A A A A A A
B A A B A A A A A A A B A B B A A B A
B A A A A A B B B B A B A A B A A A A
B X A B B
satt425 B B A A A A A A B A B A A A A B A A
A A B B B A A A A B A A A B B A B B A
A A A A A A B A A A A A A A A A A B B
B B B A B B B B A A B B B B B A B A B
B B B A A A A A A A A B A B B B B A B
B B A A A A A B A A A A A B A A A A A
B A A B A A A A A A A B A B B A B B A

B	A	A	B	A	A	B	B	B	B	A	A	A	A	B	B	A	A	B
B	A	A	B	B														
satt168	B	A	A	A	A	A	A	A	A	A	A	A	A	A	A	A	A	A
B	A	A	A	A	A	A	A	A	A	B	A	A	A	A	A	A	A	A
A	A	A	A	A	B	A	A	B	A	A	A	A	A	A	A	A	A	A
A	A	A	A	B	A	A	A	A	A	A	A	A	A	A	A	A	A	A
A	A	A	A	A	A	A	A	B	A	A	A	B	B	A	A	A	A	A
A	A	B	B	B	B	B	B	A	A	A	A	A	A	A	A	A	A	A
B	B	A	B	B	A	A	B	A	A	B	A	A	B	A	A	A	A	A
B	A	A	A	A	A	A	A	A	A	A	A	A	A	A	A	A	X	A
A	A	A	A	A														
sat_009	A	A	X	X	A	A	A	A	A	X	A	A	X	A	X	A	X	A
A	X	A	A	X	A	A	A	X	A	A	A	X	X	X	A	X	A	A
A	X	X	X	A	X	X	A	X	X	X	X	A	X	A	A	A	A	A
A	X	X	X	A	X	A	X	A	A	A	X	A	A	A	A	A	A	A
X	A	X	A	X	A	X	X	A	X	X	X	X	A	X	A	A	A	X
A	X	A	X	A	A	A	A	X	A	X	A	A	A	A	X	A	A	A
B	A	X	A	B	A	A	A	A	X	A	X	X	A	A	A	A	X	A
B	X	X	X	A	A	A	X	A	X	A	A	A	A	A	A	A	A	A
X	A	X	A	A														
satt474	A	A	A	A	A	A	A	A	A	A	A	A	B	A	B	A	B	A
A	B	A	A	B	A	A	A	A	A	A	A	A	B	B	A	A	A	A
A	B	B	B	A	B	A	B	A	A	A	A	A	A	A	A	A	A	A
A	B	B	B	A	B	A	B	A	A	A	B	A	A	A	A	A	A	A
B	A	B	A	B	A	B	B	A	B	B	B	B	A	A	A	A	A	A
A	A	A	B	A	A	A	A	B	A	A	A	A	A	A	B	A	A	A
A	A	A	A	B	A	A	A	A	B	A	A	B	A	A	A	A	B	A
B	B	B	B	A	A	A	A	A	A	A	A	A	A	B	A	A	A	A
A	A	A	A	B														
Sat_230	A	B	B	B	A	A	A	A	B	B	A	A	B	A	B	A	A	A
A	B	A	A	B	A	A	A	B	A	A	A	A	B	B	A	B	B	B
A	B	B	A	B	A	B	A	A	X	B	A	A	A	X	A	A	A	A
A	B	B	B	A	B	A	B	A	A	A	A	A	A	A	A	A	A	A
B	A	B	A	A	A	B	B	A	B	B	B	A	A	A	B	A	A	A
B	B	B	B	A	A	A	A	B	A	A	A	A	A	A	X	A	A	A
A	A	B	B	A	A	A	A	A	B	A	A	B	A	A	A	B	A	B
A	B	B	B	A	B	B	A	A	B	B	A	B	A	B	A	A	A	A
A	A	A	A	A														
Satt070	A	B	B	B	A	A	A	A	A	A	A	A	B	A	B	A	B	A
A	B	A	B	A	B	A	A	B	A	A	A	B	B	B	A	B	A	X
A	B	A	B	A	X	B	A	A	A	A	A	A	A	B	A	A	A	A
A	A	B	B	A	B	A	B	A	A	A	B	A	A	A	A	X	A	A
A	A	A	B	A	A	A	A	A	A	A	B	B	A	A	B	A	A	B
A	X	B	A	A	A	A	A	A	B	A	B	A	A	X	B	A	A	A

B	B	A	B	A	B	A	A	A	B	A	B	B	A	A	A	A	B	B
B	B	A	B	A	B	A	A	A	A	A	A	A	B	A	A	A	A	A
A	B	A	B	B														
satt577	A	B	A	B	A	A	A	A	A	B	A	A	B	A	B	A	A	A
A	A	A	A	B	A	A	A	B	A	A	A	B	B	A	A	B	B	A
A	B	B	B	A	B	A	A	A	A	A	A	A	A	A	A	A	A	A
A	B	B	A	A	B	A	A	B	B	A	A	A	A	A	A	A	B	A
A	X	A	B	A	B	B	A	B	B	B	B	B	A	A	A	B	A	A
B	A	A	A	A	B	A	A	A	A	A	A	A	A	A	A	A	A	A
B	A	B	A	X	A	B	A	B	A	A	X	A	B	A	A	A	B	A
B	B	B	A	A	A	B	B	A	A	X	A	B	A	A	B	A	A	B
B	B	B	A	B														
satt231	A	A	B	B	A	A	A	A	A	B	A	B	A	A	A	A	A	B
B	A	A	B	A	B	A	A	B	A	A	A	B	B	B	A	A	B	A
A	A	A	B	A	A	B	A	A	A	B	A	A	B	A	A	A	B	A
A	A	B	A	A	A	A	B	B	B	A	A	A	A	A	A	B	A	A
A	A	A	B	A	A	B	A	B	B	B	B	A	A	A	A	A	A	A
A	A	A	A	A	B	B	A	A	A	A	A	A	B	A	B	A	A	A
A	A	A	A	B	A	B	A	A	A	A	B	B	A	A	A	A	B	A
A	A	A	B	A	B	A	B	A	B	A	A	A	A	A	B	A	A	B
A	A	A	A	A														
satt685	A	A	A	A	A	A	A	A	A	A	A	A	A	A	A	A	A	A
A	A	A	A	A	A	A	A	A	A	A	A	A	A	B	A	A	A	A
A	A	A	A	A	A	A	A	A	B	B	A	A	A	A	A	A	A	A
A	A	A	A	A	A	A	A	A	A	A	A	A	A	A	A	A	A	A
A	A	A	A	A	A	A	A	A	A	A	A	A	A	A	A	A	A	B
A	A	A	A	A	A	B	A	A	A	A	A	A	A	A	A	A	A	B
B	A	A	A	B	A	A	A	A	A	A	A	A	B	A	B	A	A	A
B	A	B	A	A	A	A	B	A	B	A	B	A	A	A	A	A	B	B
A	A	A	A	A														
sat_136	A	A	A	A	A	A	A	A	A	A	A	A	A	A	A	A	A	A
A	A	A	A	A	A	A	A	A	A	A	A	A	A	A	A	A	A	A
A	A	A	A	A	A	A	A	A	A	A	A	A	A	A	A	A	A	A
A	A	A	A	A	A	A	A	A	A	A	A	A	A	A	A	A	A	A
A	A	A	A	A	A	A	A	A	A	A	A	A	A	A	A	A	A	A
A	A	A	A	A	A	B	B	A	A	A	A	A	A	A	A	A	A	A
A	A	A	A	A	A	A	A	A	A	A	A	A	A	A	A	A	A	A
A	A	A	A	A	A	A	A	A	B	A	A	A	A	A	A	A	A	A
A	A	B	A	A														
satt651	A	X	A	A	A	A	A	A	B	A	A	A	B	A	A	A	A	B
B	A	A	B	B	B	A	A	B	A	A	A	A	A	A	A	A	B	B
A	A	A	B	A	X	A	A	A	A	X	X	A	A	X	A	A	A	A
A	A	A	A	A	A	A	B	A	B	A	A	B	A	A	A	B	A	A
A	A	B	A	A	A	A	A	A	A	B	B	A	A	A	A	A	A	X

A	B	A	A	A	A	B	B	B	A	X	B	A	A	A	B	A	A	A
X	A	B	A	B	A	A	A	A	B	A	X	A	A	A	X	A	B	A
B	B	B	B	A	A	X	X	B	A	X	A	B	A	B	B	A	A	X
A	B	B	B	A														
Satt045	B	B	A	B	B	B	B	X	A	B	B	B	B	B	B	B	B	A
A	A	X	A	A	A	B	B	A	B	B	B	B	B	B	B	A	A	B
B	B	A	B	B	B	X	B	B	B	X	B	X	B	B	B	B	B	X
B	B	A	A	B	B	B	A	X	X	B	A	B	A	B	B	A	B	B
B	B	A	B	B	B	B	B	X	B	X	B	B	B	A	A	B	B	B
B	A	B	B	B	B	X	B	B	B	X	B	B	A	B	A	B	B	B
X	B	B	B	B	B	B	B	B	B	B	A	B	B	B	B	B	B	B
B	B	A	X	B	B	B	A	B	A	B	B	B	B	B	A	B	B	B
B	A	A	A	B														
Satt483	A	A	B	A	A	A	A	B	B	A	A	A	A	A	A	A	A	B
B	B	A	B	B	B	A	A	B	A	A	A	A	A	A	A	B	B	A
A	A	B	A	A	A	A	A	A	A	B	B	A	A	A	A	A	A	B
A	A	B	B	X	X	A	B	B	B	A	B	X	B	A	A	B	A	A
A	A	B	A	A	A	A	A	A	A	A	A	A	A	B	B	A	A	A
A	B	A	A	A	X	B	B	A	A	B	A	A	B	A	B	A	A	A
A	A	A	A	B	A	A	A	A	A	B	B	A	A	A	A	A	B	A
A	A	B	A	A	A	A	B	A	B	A	A	A	A	A	B	A	A	A
A	B	B	B	A														
Satt553	X	X	B	B	B	B	B	B	B	A	B	A	A	A	A	A	A	A
A	B	A	B	B	A	A	A	A	B	B	A	B	A	A	B	B	B	A
B	B	B	B	A	A	B	B	B	A	B	B	A	A	A	B	A	B	A
B	B	A	B	A	A	A	A	A	B	B	B	B	B	B	B	A	A	A
B	B	A	B	A	B	B	B	B	B	B	B	A	B	A	A	B	A	A
B	A	B	B	B	B	A	A	B	B	A	B	B	A	B	A	B	B	B
B	B	B	B	A	B	B	B	B	B	A	A	A	B	B	B	A	B	A
B	B	B	A	B	B	B	A	B	A	B	B	B	B	B	A	B	B	A
A	A	A	X	A														
Satt596	A	A	B	B	B	B	B	X	B	A	A	A	B	A	X	B	A	B
A	A	B	B	A	B	B	B	B	B	X	A	B	B	B	A	A	B	X
A	B	B	A	A	B	X	X	B	X	B	X	X	B	A	X	A	B	A
A	A	B	B	A	A	A	B	A	A	A	B	B	A	B	A	A	X	B
A	A	B	A	B	A	A	A	A	B	B	A	B	A	B	B	A	A	B
A	A	B	B	B	B	A	A	B	A	B	A	A	B	A	B	A	A	A
A	A	B	B	B	B	A	A	A	A	A	B	B	A	A	A	A	B	A
X	A	B	B	A	A	A	B	B	B	A	A	B	A	B	B	A	A	B
B	A	B	A	A														
Satt414	A	A	A	B	A	A	A	A	A	B	A	A	B	A	A	A	B	B
A	B	A	B	B	A	A	A	B	A	A	A	A	B	B	A	A	A	A
A	A	A	B	A	B	B	A	A	A	A	A	A	B	A	A	A	B	A
A	A	A	A	A	A	A	B	A	B	A	B	A	A	A	A	A	B	B

A A A A B A A B A A B X A A B B A A B
A A A A A A B A B A B A A B A B A A A
A A A B B A A A B A A A B A A A A B B
B B B B A A A B A B A A A A A B A A A
B B A B B
Sat_255 B B B B A B B B B B A B A B B B A A
A A B A A A B B A B B B A A A B X A B
B B B A B A A B B B B B B A B B B B A
B B B B B B B X B A B A B B B B B A A
B B B B A B B A B A A B A B A A B B B
X A B B B B B B B B B B B A B A B B B
B B X B B X B B B B A A A B A A A B B
B A B B X A B B B A B B A B A X B B B
B A B A A
sat_394 A A A B A A A A A B A A A A A A B A
A A A A A A A A B A A A X B A A A A A
A A A B A A A A A A B A A A A A A A A
A A A A A A A B B A X A B B A A A A A
A A A A A A B B A X A A A A A A A A A
A A A A A A A A A A A A A A A A A A A
A A A A A A A A A A A A B A A A B A A
A A A A A B A A A A A A A B A A A A A
A A A A A
Sat_366 A A A A A A A A A A A A A A A A A A
A A A A A A A A A A A A A A A A A A A
A A A A A A A A A A A A A A A A A A A
A A A A A A A A A A A A A A A A A A A
A A A A A A A A A A A A A A A A A A A
A A A A A A A A A A A A A A A A A A A
A A A A A A A A A A A A A A A A A A A
A A A A A A A A A A A A A A A A A A A
A A A A A
sat_228 A A A A A A A A X A A X A A A A X A
B A B B A A A A X A A A X A X B A A X
A A X A A A X A A A A A A A A X A A X
X A X A A X A A A A A X A A X A A A A
A A A A A X X X A X A A A A B A A A B
A A X A A A X X X A A X X X A A A A A
A A A A B A A A A A A A A B A A A B A
B A A A A A B A A A A B B A A A A A X
A A B A A
Sct_065 B B B B B B B B B A B B B B B B B A
A B B B A B B B B A B B A A A B B B A
B A A A B A A B B A B A A B A B B A B

B A B B B B B A B A B X A B B B B B B
B A A B A B A B B B B B B A B B B A B
A A B B A B A B B B B B B B B B A B B
A A B B B A B B B A B B A A B B B X B
X B A B A B A B A B B B B A B B B B B
B A X B A
satt154 A A A A A A A A A A A A A A A A A A
B A A A A B A A A A A A A A A A A A A
A A B A A A B A A A A A A A A A A A B
A A B B A A A A A A B B B A B B A A A
A B A A A A B A B A A A A A A B A A A
A A A A A A B B A A A A A A A A A A A
A A A B A A A A B B A A A B A B A B A
A A B B A A B B A A A A A A A A A A B
A A B A A
Satt669 A A A A A A A B A A A A B A B A A B
A B A A B A A A A A A A A A A A A A A
A B B A A A A A A A A B A A B A A A A
A A B A A A B A B B A B B A A A A B B
A A A X B A A A A A A A A A A A A A A
A A A A A A B B B A A A A A A A A A A
A A A B A A A A A B A A A B B A A A A
A B A B A A A B A B A A B A B A A A B
B B B B A
sat_326 A A A A A A A A B A A X A A A A A A
A A A B X A A A B A A A X A A A X A A
A A X X A A A A A A X A A X A A A A A
A A A B A X A B A A A B A A A A B A B
A A A B A A A A A A B A B A B A A A B
A B A A A B B B A A A B A A A A A A A
A A A B A A A A A A A B B A B A A A A
A A A A A A A B A B A A A A B B A A A
A A A A A
satt186 A A B A A A A A B B A B A A A A A A
A A A A A A A A B A A A B B B A B A B
A B B B A A B A A A A A A B A A A B A
A A B B A A A A B A A B A B A A B A B
A A A A B A A A A A B B B A B A A A X
A B A A A A B B B A A A A A A A A A A
A A B B B A A A A A A B B A B A A B A
X A B A A A X B A B A A A A B A A A B
A A B A B
sat_333 A A B A A A A A A B A A A A B A B X
A A A A A A A A A A A A B B A A A B X

A	A	A	A	A	B	B	A	A	A	A	A	A	B	A	A	A	B	A
A	B	A	A	A	A	A	A	B	A	A	A	A	A	A	A	B	X	X
A	A	X	A	X	A	A	A	A	B	A	A	B	A	A	A	A	A	A
A	B	A	A	A	B	A	A	A	A	A	A	A	A	A	A	A	A	A
A	A	B	B	A	A	A	A	A	B	A	B	B	X	B	A	A	A	A
A	A	A	B	A	A	B	A	A	B	A	A	A	A	B	A	A	A	A
B	A	X	A	B														
sat_194	A	A	B	B	A	A	A	A	A	A	A	A	A	A	B	A	A	B
A	B	B	B	A	A	A	A	A	A	A	A	A	A	A	A	A	A	B
A	A	A	A	A	A	B	A	A	A	A	A	A	B	A	A	A	B	A
A	A	A	A	A	A	A	A	A	A	A	B	A	B	A	A	B	A	A
A	A	A	A	B	A	A	A	A	B	A	B	A	A	B	A	A	A	A
A	B	A	A	A	A	A	A	A	A	A	A	A	A	A	B	A	A	A
B	A	A	B	B	A	A	A	A	A	B	B	B	A	B	A	A	A	A
B	A	B	A	A	A	A	B	A	B	A	A	A	A	B	A	A	A	B
B	A	A	A	A														
sat_001	B	B	B	A	A	A	A	A	X	A	A	A	B	B	A	A	A	B
B	A	A	A	A	A	B	A	B	A	A	A	B	A	B	B	A	X	X
A	A	A	A	A	A	A	B	A	A	A	B	A	A	A	A	B	A	A
A	B	B	B	A	A	B	A	A	A	A	B	B	A	B	A	A	A	B
B	B	A	A	B	A	A	A	A	B	A	B	A	A	B	X	A	A	A
A	A	A	A	A	A	B	A	A	A	A	A	A	A	A	B	A	A	A
A	B	A	A	B	B	A	A	A	A	A	A	A	A	B	A	A	A	A
B	B	B	A	A	A	A	B	A	B	A	A	A	A	A	A	A	A	A
B	A	A	A	B														
sat_403	A	B	A	B	A	A	A	A	B	A	A	B	A	A	B	A	B	B
B	B	A	B	A	B	A	X	B	A	A	A	X	A	A	A	B	A	B
A	B	B	X	A	B	B	A	A	A	A	A	X	B	A	A	A	A	A
A	B	B	B	X	B	A	A	B	B	A	B	B	A	X	A	A	A	A
B	A	B	A	B	A	B	A	A	B	B	B	B	B	A	B	A	A	B
A	A	A	B	A	B	X	A	B	A	A	B	A	B	A	A	A	A	A
B	X	A	A	B	X	A	A	A	B	A	B	B	A	A	A	A	B	A
B	A	A	B	A	A	B	A	B	A	A	A	A	A	B	B	A	A	B
B	A	B	A	B														
satt309	A	B	B	B	A	A	A	A	A	A	A	B	A	A	B	A	B	A
A	A	A	A	B	A	A	A	B	A	A	A	A	A	A	A	A	A	A
A	A	A	B	A	B	B	A	A	A	A	A	A	B	A	A	A	B	A
A	B	B	A	A	A	A	A	B	B	A	A	B	A	A	A	A	A	A
B	A	B	A	A	A	B	A	A	A	A	A	B	A	B	A	A	A	A
A	B	A	A	A	A	A	B	A	A	A	B	A	B	A	A	A	A	A
B	A	B	B	B	A	B	A	A	A	A	B	B	A	B	A	A	B	A
A	A	A	A	A	A	B	B	B	B	A	A	B	A	B	A	A	A	B
B	B	B	A	B														
sat_210	A	B	B	B	A	A	A	B	A	X	A	A	B	A	B	A	B	A

A	A	B	A	B	A	X	A	B	A	A	A	A	A	A	A	A	A	A
A	A	A	B	A	A	B	A	A	A	A	A	A	B	A	A	A	A	A
A	A	A	A	A	A	A	A	A	A	A	A	B	A	A	A	B	B	A
B	A	B	A	B	A	B	A	A	A	A	A	B	A	B	A	A	A	A
A	B	A	B	A	A	A	A	A	A	A	B	A	A	A	A	A	A	A
A	A	A	A	X	A	B	A	A	A	A	B	X	A	A	A	A	X	A
B	B	A	A	A	A	A	A	B	B	A	A	B	A	B	A	A	A	A
B	X	B	A	B														
AW734137	A	A	B	B	A	A	A	A	B	A	A	A	A	A	B	A	B	B
A	B	A	A	B	A	X	B	A	A	A	A	A	A	A	A	A	A	A
A	A	A	A	A	B	B	A	A	A	A	A	A	B	A	A	A	B	A
B	A	A	A	A	A	A	A	B	B	B	A	B	A	A	A	A	B	A
B	A	X	A	X	A	B	B	A	A	A	A	B	A	A	A	A	A	A
A	B	A	B	A	A	A	A	B	A	A	A	A	A	A	A	A	A	A
B	A	B	B	B	A	A	A	A	A	A	B	B	A	A	A	A	B	A
A	B	B	A	A	B	A	A	A	B	A	A	A	A	A	A	A	A	A
A	A	B	A	A														
satt570	A	A	A	B	A	A	A	A	A	A	A	A	B	A	B	A	B	B
B	B	A	A	B	A	A	A	B	A	A	A	A	A	A	A	A	B	A
A	A	A	B	A	B	B	A	A	A	A	B	A	B	A	A	A	A	B
B	X	B	A	A	A	A	A	B	B	A	A	B	A	A	A	A	A	A
A	B	B	A	X	X	A	X	A	X	B	X	A	A	A	A	A	A	B
A	X	A	A	A	A	A	B	A	A	A	A	A	A	A	A	A	A	A
B	A	B	B	B	A	A	A	A	A	A	B	B	A	B	B	A	B	A
X	B	X	A	A	A	A	A	A	X	A	A	X	A	A	A	A	A	A
B	B	B	A	B														
satt688	A	B	A	A	A	A	A	A	B	A	A	A	B	A	A	A	A	B
B	A	A	A	B	A	A	A	B	A	A	A	A	A	B	A	A	A	A
A	A	A	B	A	B	A	A	A	A	B	B	A	A	A	A	A	A	A
A	A	B	B	A	A	A	B	A	B	A	B	B	A	A	A	B	A	A
A	A	B	A	A	A	A	A	A	A	A	B	A	A	A	A	A	A	A
A	A	A	A	A	A	B	B	A	A	A	B	A	A	A	A	A	A	A
A	A	B	B	A	A	B	A	B	B	A	A	A	B	A	B	A	B	A
A	B	B	A	A	A	B	B	B	A	A	A	A	A	B	A	A	A	B
A	B	A	B	A														
Sat_372	B	X	A	A	A	A	A	A	A	A	A	B	A	A	B	B	A	A
A	A	X	A	A	A	A	A	A	A	A	A	X	X	A	X	A	A	A
A	A	A	A	A	A	B	A	A	A	B	X	A	B	X	A	A	A	A
A	A	B	A	A	A	X	A	B	A	A	B	A	X	A	A	A	A	A
A	A	A	A	X	A	A	A	A	A	A	A	A	B	A	B	A	A	A
A	A	A	A	A	A	B	B	A	A	X	A	A	A	A	X	A	A	A
B	A	A	B	B	X	A	A	A	A	A	X	X	B	A	B	A	X	A
B	A	A	A	A	X	X	X	B	B	A	A	A	A	X	A	A	X	X
A	X	A	A	A														

sat_117	A	A	A	A	A	B	A	A	A	B	A	A	A	A	A	A	A	A
A	A	A	A	A	A	A	A	A	B	X	A	B	B	B	A	A	A	A
A	A	A	A	A	A	B	A	A	A	A	A	A	B	A	A	A	A	A
A	A	A	A	A	A	A	A	A	A	A	A	A	B	A	A	A	A	A
A	A	B	A	B	B	A	A	A	A	A	A	X	A	A	A	A	A	B
A	A	A	A	A	A	B	A	A	A	A	A	A	A	A	A	A	A	A
B	X	A	B	A	A	A	X	A	A	A	A	A	B	A	A	B	A	A
B	A	A	A	A	B	A	A	A	B	A	A	B	A	A	A	A	A	A
A	A	A	A	A														
satt352	A	A	A	A	A	A	A	A	A	A	A	A	B	A	A	A	A	A
A	A	A	A	A	A	A	A	A	A	A	A	A	A	A	A	A	A	A
A	A	A	A	A	A	A	A	A	A	A	A	A	A	A	A	A	A	A
A	A	A	A	A	A	A	A	A	A	A	A	A	A	A	A	A	A	A
A	A	A	A	A	A	A	A	A	A	A	A	A	A	A	A	A	A	A
A	A	A	A	A	A	A	A	A	A	A	A	A	A	A	A	B	A	A
A	A	A	A	A	A	A	A	A	A	A	A	A	B	A	A	A	A	A
A	A	A	A	B	A	A	A	A	A	A	A	A	A	A	A	A	A	A
A	A	A	A	A														
satt564	A	A	A	A	A	A	A	A	A	A	A	A	A	A	A	A	A	A
A	A	A	A	A	A	A	A	A	A	A	A	A	A	A	A	B	A	A
A	A	A	A	A	A	A	A	A	A	A	A	A	A	B	A	A	A	A
A	A	A	A	A	A	A	A	A	A	A	A	A	A	A	A	A	A	A
A	A	A	B	A	A	A	B	A	A	A	B	A	A	A	B	A	A	A
A	B	A	A	A	A	B	A	A	A	A	A	A	A	A	A	A	A	A
A	A	A	B	A	A	A	A	A	A	A	A	A	B	A	A	A	B	A
A	A	B	A	A	A	B	A	A	A	A	A	B	A	A	A	A	A	A
A	A	A	A	A														
sat_203	A	A	B	B	A	A	A	A	B	A	B	A	A	A	B	A	B	B
X	A	A	A	A	A	A	A	A	A	A	A	B	A	A	B	B	A	X
A	A	B	B	A	A	A	A	A	A	A	A	A	A	A	A	A	A	A
A	A	B	A	A	B	A	A	B	A	A	A	B	A	A	A	B	A	B
X	A	A	A	A	A	B	A	A	B	A	A	A	A	B	B	A	A	A
A	B	A	A	A	A	B	A	A	A	A	A	A	A	A	A	A	A	A
A	A	A	B	A	A	A	A	A	B	A	X	A	B	A	A	A	B	A
A	A	A	B	A	A	B	B	A	B	A	A	A	A	B	A	A	A	A
X	B	A	A	A														
satt373	A	B	B	B	A	A	A	A	B	B	A	A	B	A	B	A	B	A
A	B	A	A	B	B	A	A	A	A	A	A	B	B	A	A	B	B	X
A	B	B	A	A	B	A	A	A	A	A	A	A	A	A	A	A	A	A
A	A	A	A	A	B	A	B	B	A	A	B	B	A	A	A	B	B	A
B	A	A	A	B	A	B	A	A	A	B	A	A	A	A	A	A	A	B
A	A	A	A	A	B	B	A	A	A	A	A	A	A	A	A	A	A	A
B	A	B	B	A	A	A	A	A	B	B	A	A	B	B	A	A	B	A
B	B	B	B	A	A	A	A	B	A	A	A	B	A	B	B	A	A	B

A A B B B

satt313 A B A B A A A A B B A B B A A A A B
B B A B B B A A A A A A B B A A B B B
A A B B A B A A A A A A A A A A A A A
A A B A A B A A B A A A B B A A A A B
A A B A A A A A A A B B A B A A A A B
A A A A A B A A A A A A A A A A A A A
B A A A A A A A A B A B A B B A A B A
B B B B A B B B A A B A B A A B A A B
B A B A B

sat_191 A B A B A A A B B B A B B A A A A B
B B A B B B A A A X A A B B X A B B X
A A B A A X A A A A A A A A A A A A X
A A B A A B A A B A A X B B A A A X B
X A B A A A A A A A B B A A B A A A B
A A A A A B A A A A A X A A A A A A X
A A A A A A A A A B A X A B B X A B A
B B B B A A B B A A B X A A A B A X B
B A B A B

sat_405 A A A B A A B A A B A B B A A A A B
B B A B B B A A A A A A B B A A B B A
A A B A A A A A A A A A A A A A A A B
A A B B A B A A A A A A B B A A A A A
A A A A A A A A A A A B A A B B A A B
A A A A A B A A A A A A A A A A A A A
B A A A A A A A A A A A A B A A A B A
B A A B A A A B B A A B B B A A B B A
A A A B A

sat_195 A A A A A A A A A A A B A A A A A A
A A A A A A A A A A A A A A A A A A A
A A B A A A A A A A A A A A A A A A A
A A A A A A A A A A A A A A A A A A A
A A A A A A A A A A A A A A A A A A B
A A A A A A A A A A A A A A A A A A A
A A A A A A A A A A A A A B A A A A A
A A A A A A A A A A A A A A A A A A A
A A A A A

satt448 A A B B A A A A A A A A A A B A A A
A B A B A A A A A A A A A A A A A A A
A A A A A A B A A A A A A B A A A B A
A A A A A A A A A A A B A B A A B A A
A A A A B A A A A B A B B A B A A A A
A B A A A A A B A A A A A A A B A A A
X A A B B A A A A A X X A A B A A A A

A A B A A A A B A B A A A A A A A A B
B A A A A
satt182 A A A B A A A A B A A B B A A A A B
A B A B B B A A A A A A A A A A B B A
A A B A A A B A A A A A B A A A A B A
A B A A A B A A A A A B A A A A A B B
A A A A B A A B A A A A B A B A A A A
A A A A A B A A A A A B A A A B A A A
A A A A B A B A A B A A B B B A B A A
B B B B A A A B A A A A B A B B A A A
A A B A A
sat_268 A A B B A A A A A A B A B A A A A B
A B A B B B A A B A A A A A A B A A B
A B B A A A A A A B A A A A A A A B A
A A B A A B A B A A A B A A A A B B B
A A A A A A B B A A B A A A A A A A A
A B A A A B A A A A A A A A A A A A A
A A B A A A A A A A B B A A A A A B A
A A B B A A B A A B A A A A B A A A B
A A A B B
sat_170 A A A B A A A A B B A B A A B A A A
A B A A A A A A B A A A B A A A B B B
A A B B A B A A A A A A A A A A A A A
A A B A A B A A A B A B B A A A B A A
B A B A A A B A A A A A A A B A A A B
A A A A A A A A A A A A A A A B B A A
B A A A A A A A A B A A A A A A A B A
B A A A A A B A A A B A B A B A A A A
B B B A A
satt330 A A A B A A A A B B A A A A B B A A
A B A A A A A A B A A A B A B A B B B
A A B B A B A A A A A A B A A A A A A
A A B B A B A A A B A B B A A A A B A
A A B A A A B A A B A A A A B A A A B
A A A A B A A A A A A B A A A B A A A
A A A A A A A A A B A A A A B A A A A
B A A A A A A A B A A A B A B A A A A
B B B A A
satt367 A A A A A A A A A A A A A A A A A A
A A A A A A A A A A A A A A A A A A A
A A B A A A A A A A B A A A A A A A A
A A A A A B A A A A A A A A A A A A A
A A A A A A A A A A A A A A A A A A A
A A A A A A A A A A A A A A A A A A A

A A B A A A A B A A A A A A A A A A A
A A A A B A A A B A A A B A A A B A A
A A B A A
satt571 A A A A A A A B A A B B A B A B A A
A A A A A A B B B A A A A A A A A A A
A B A A A A A A B A B B A B B A B A A
A A A A A A A A A A A A A A A A A A A
A A A B A A A B A B A X A A A B A A A
B B A A A A A A A A A A A A A A A A A
X A B B A B B B B A A A A B A A A X A
B B X B A A X A A A X A X A A A A A X
X A A A A

Phenotypic Data

PNUA 1 3 3 2 1 3 1 1 1 1 2 3 2 1 4 5 1 4 2
1 1 2 1 -100 2 1 1 1 4 1 1 5 1 1 1 1 1 1
4 1 1 1 1 2 5 1 1 1 3 2 2 2 1 2 8 1
-100 -100 1 3 -100 2 1 1 3 2 2 1 1 1 2 1 2 2 2
6 1 3 1 -100 3 2 1 2 2 1 1 3 1 2 1 1 2 1
3 2 1 2 1 3 2 1 3 2 1 1 -100 1 1 1 2 1 3
2 2 2 1 1 6 3 1 -100 1 1 -100 5 1 1 1 9 3 1
1 1 1 -100 3 1 1 2 -100 1 1 1 2 1 1 1 1 1 1
2 2 2 2 -100
PNMA 3 5 3 3 1 1 1 1 1 2 1 2 1 5 2 1 3 2 2
1 1 2 2 -100 1 2 2 1 1 1 1 3 1 4 1 5 1 2
4 1 1 1 4 3 3 2 1 6 2 3 1 2 7 1 4 6
-100 -100 1 1 -100 2 2 1 5 2 2 1 2 4 2 2 3 1 1
1 1 1 1 -100 2 1 3 1 3 4 4 3 2 1 2 2 1 1
1 1 1 1 2 1 2 1 3 2 1 1 -100 1 1 1 1 3 5
2 3 1 1 2 2 5 7 -100 1 1 -100 1 4 2 2 3 3 2
2 1 1 -100 1 1 4 3 -100 1 1 1 1 1 1 1 1 4 4
6 1 2 1 -100
PNDA 2 4 4 2 1 1 1 1 1 2 1 3 1 2 2 4 3 1 1
1 1 1 2 -100 1 1 3 2 2 1 1 3 1 1 1 1 1 1
2 1 3 1 7 2 2 3 1 4 2 1 1 3 1 2 2 4
-100 -100 2 1 -100 2 2 1 3 2 1 1 1 4 2 1 3 1 1
4 1 3 1 -100 2 1 1 1 2 11 2 1 2 2 2 1 1 1
1 4 1 1 1 2 1 1 3 3 1 1 -100 1 2 1 1 1 4
1 2 2 2 1 1 3 1 -100 2 1 -100 2 4 4 3 1 1 1
2 1 1 -100 2 2 2 3 -100 1 1 1 2 1 2 1 1 1 2
2 1 2 1 -100
PNUB 8 2 5 5 2 6 3 4 2 6 5 5 3 11 13 3 3 4 3
1 3 4 8 -100 4 2 5 3 3 2 2 12 1 10 1 4 3 4

9 4 5 5 9 3 4 5 2 4 2 3 2 8 1 5 11 8
-100 -100 2 7 -100 6 8 4 6 3 4 4 7 2 3 2 6 3 6
10 7 5 2 -100 16 4 3 3 5 5 7 9 8 5 3 5 2 2
6 6 3 2 2 3 6 5 5 3 5 4 -100 4 4 3 7 2 2
5 3 4 6 8 10 4 9 -100 4 3 -100 12 8 2 2 3 4 5
3 1 7 -100 7 4 11 3 -100 3 2 2 5 2 4 1 2 4 6
10 4 6 6 -100
PNMB 9 8 7 12 3 6 5 3 2 3 4 11 1 18 5 9 1 7 1
2 4 7 12 -100 2 3 3 4 10 14 3 10 3 8 2 4 6 5
7 2 3 8 12 11 5 6 1 7 7 4 6 7 6 9 11 4
-100 -100 2 10 -100 10 5 2 6 5 3 3 5 9 5 8 8 3 3
12 7 7 2 -100 11 12 3 4 3 8 5 6 9 5 6 1 3 2
6 11 3 3 2 6 7 4 4 8 14 6 -100 6 9 2 6 8 8
3 3 6 6 4 13 16 13 -100 6 3 -100 14 7 7 3 8 6 2
5 2 5 -100 5 3 10 10 -100 4 1 5 6 3 5 2 5 6 4
16 2 9 6 -100
PNDB 6 6 6 9 1 2 5 1 7 3 4 7 2 9 7 3 2 8 1
2 2 5 5 -100 4 3 6 4 4 4 2 8 1 7 1 6 2 5
4 2 3 5 6 2 4 4 2 4 5 4 4 7 4 2 8 6
-100 -100 4 6 -100 6 2 3 4 5 5 2 2 5 2 3 3 3 3
9 3 2 3 -100 9 7 1 3 3 9 6 6 6 6 2 2 2 4
9 6 2 3 1 4 5 3 3 6 9 2 -100 3 9 1 2 6 6
4 2 5 1 3 6 6 4 -100 5 4 -100 10 5 8 2 7 2 4
3 1 6 -100 3 2 5 4 -100 4 3 3 3 3 2 1 1 6 4
13 4 3 6 -100
PNUC 2 8 5 7 5 2 5 3 4 3 9 5 2 12 9 3 5 5 3
2 4 6 6 -100 4 7 7 5 6 6 8 5 4 5 2 8 3 3
4 4 5 11 3 9 10 6 2 8 6 7 8 3 8 6 3 5
-100 -100 5 2 -100 2 6 3 3 5 2 5 8 4 4 6 4 2 5
10 10 7 9 -100 2 12 6 2 4 5 7 7 4 4 4 16 5 7
9 17 6 7 3 7 2 3 9 4 7 6 -100 9 11 2 3 8 8
7 6 6 5 5 9 6 3 -100 9 3 -100 5 4 9 7 4 3 10
6 3 8 -100 10 3 14 7 -100 8 5 4 3 1 3 3 3 4 3
11 2 4 6 -100
PNMC 13 5 3 9 5 4 7 6 10 4 8 12 6 11 7 8 4 6 5
3 4 6 11 -100 9 9 9 7 8 6 5 8 3 8 3 12 3 2
7 6 11 12 7 8 6 10 8 12 12 8 5 13 2 11 5 6
-100 -100 10 16 -100 4 9 7 6 8 9 5 9 7 7 2 5 3 9
8 7 6 11 -100 7 8 4 3 10 7 11 7 12 11 4 12 3 7
8 13 11 8 3 4 9 7 11 9 8 4 -100 5 11 1 7 6 5
7 6 4 6 6 7 10 6 -100 6 4 -100 7 8 9 8 14 3 7
6 2 11 -100 6 4 22 19 -100 5 8 2 8 6 1 3 5 7 8
9 9 5 7 -100
PNDC 8 6 2 5 4 4 5 3 4 5 3 9 1 6 6 7 4 4 4

	3	2	9	12	-100	3	7	1	2	6	8	4	5	2	7	2	7	2	6
	5	8	5	12	4	9	3	4	6	4	5	3	3	5	5	5	4	1	
	-100	-100	6	8	-100	3	5	4	5	3	2	2	12	3	7	7	2	4	5
	5	6	4	7	-100	4	5	5	3	4	3	6	6	3	7	2	4	2	6
	7	10	5	6	3	3	3	3	5	4	7	4	-100	2	8	2	6	6	6
	2	7	5	4	8	7	7	4	-100	1	3	-100	6	3	9	4	11	3	5
	2	3	3	-100	4	2	18	4	-100	2	4	1	5	3	2	2	3	4	5
	10	5	3	8	-100														
PNUD	1	3	1	1	3	1	1	1	1	1	1	1	1	1	1	2	1	1	1
	1	2	1	1	-100	1	1	1	1	1	1	7	1	1	1	1	1	1	1
	1	6	1	1	1	7	1	3	3	1	1	1	2	1	1	1	1	1	
	-100	-100	4	1	-100	1	1	3	2	1	1	3	2	1	2	1	1	1	1
	1	1	1	1	-100	1	1	2	1	1	1	1	1	1	1	1	2	1	1
	1	7	2	1	1	1	1	1	3	1	1	1	-100	1	1	1	1	1	3
	1	1	1	1	1	1	2	1	-100	1	1	-100	1	1	1	4	1	1	1
	1	3	1	-100	3	1	5	1	-100	1	5	1	1	2	1	1	1	1	1
	1	1	1	1	-100														
PNMD	1	3	1	1	2	1	1	3	1	1	1	1	2	1	1	2	1	1	1
	1	1	1	1	-100	1	1	1	1	2	1	1	1	1	1	1	1	1	1
	1	12	2	1	1	4	1	1	5	1	1	1	2	1	1	1	1	1	
	-100	-100	2	1	-100	1	2	4	3	1	1	1	4	3	5	1	1	1	2
	1	4	1	7	-100	1	1	5	1	1	3	1	1	1	1	1	4	1	1
	1	5	1	1	2	1	1	1	7	1	1	1	-100	1	1	2	1	1	3
	1	1	1	1	1	1	1	1	-100	1	1	-100	1	2	1	3	2	1	1
	1	3	1	-100	6	2	11	1	-100	1	4	1	1	1	1	1	1	1	1
	1	3	1	2	-100														
PNDD	1	1	1	1	1	1	1	2	2	1	1	1	6	1	1	1	1	1	1
	1	1	1	1	-100	1	1	1	1	1	1	2	1	1	1	1	1	1	1
	1	6	1	1	1	1	1	1	4	1	1	1	1	1	1	1	1	1	
	-100	-100	1	1	-100	1	1	1	2	1	1	1	1	1	3	1	1	1	1
	1	3	1	1	-100	1	1	3	1	1	1	2	1	1	1	1	4	1	1
	1	1	2	1	1	1	1	1	2	1	1	1	-100	1	1	1	1	1	1
	1	1	1	1	1	1	1	1	-100	1	1	-100	1	1	1	3	1	1	1
	1	2	2	-100	2	1	4	1	-100	1	1	1	1	1	1	1	1	1	1
	1	1	1	1	-100														
TPA	6	12	10	7	3	5	3	3	3	5	4	8	4	8	8	10	7	7	5
	3	3	5	5	-100	4	4	6	4	7	3	3	11	3	6	3	7	3	4
	10	3	5	3	12	7	10	6	3	11	7	6	4	7	9	5	14	11	
	-100	-100	4	5	-100	6	5	3	11	6	5	3	4	9	6	4	8	4	4
	11	3	7	3	-100	7	4	5	4	7	16	7	7	5	5	5	4	4	3
	5	7	3	4	4	6	5	3	9	7	3	3	-100	3	4	3	4	5	12
	5	7	5	4	4	9	11	9	-100	4	3	-100	8	9	7	6	13	7	4
	5	3	3	-100	6	4	7	8	-100	3	3	3	5	3	4	3	3	6	7
	10	4	6	4	-100														

TPB 23 16 18 26 6 14 13 8 11 12 13 23 6 38 25 15 6 19 5
5 9 16 25 -100 10 8 14 11 17 20 7 30 5 25 4 14 11 14
20 8 11 18 27 16 13 15 5 15 14 11 12 22 11 16 30 18
-100 -100 8 23 -100 22 15 9 16 13 12 9 14 16 10 13 17 9 12
31 17 14 7 -100 36 23 7 10 11 22 18 21 23 16 11 8 7 8
21 23 8 8 5 13 18 12 12 17 28 12 -100 13 22 6 15 16 16
12 8 15 13 15 29 26 26 -100 15 10 -100 36 20 17 7 18 12 11
11 4 18 -100 15 9 26 17 -100 11 6 10 14 8 11 4 8 16 14
39 10 18 18 -100

TPC 23 19 10 21 14 10 17 12 18 12 20 26 9 29 22 18 13 15 12
8 10 21 29 -100 16 23 17 14 20 20 17 18 9 20 7 27 8 11
16 18 21 35 14 26 19 20 16 24 23 18 16 21 15 22 12 12
-100 -100 21 26 -100 9 20 14 14 16 13 12 29 14 18 15 11 9 19
23 23 17 27 -100 13 25 15 8 18 15 24 20 19 22 10 32 10 20
24 40 22 21 9 14 14 13 25 17 22 14 -100 16 30 5 16 20 19
16 19 15 15 19 23 23 13 -100 16 10 -100 18 15 27 19 29 9 22
14 8 22 -100 20 9 54 30 -100 15 17 7 16 10 6 8 11 15 16
30 16 12 21 -100

TPD 3 7 3 3 6 3 3 6 4 3 3 3 9 3 3 5 3 3 3
3 4 3 3 -100 3 3 3 3 4 3 10 3 3 3 3 3 3 3
3 24 4 3 3 12 3 5 12 3 3 3 5 3 3 3 3 3
-100 -100 7 3 -100 3 4 8 7 3 3 5 7 5 10 3 3 3 4
3 8 3 9 -100 3 3 10 3 3 5 4 3 3 3 3 10 3 3
3 13 5 3 4 3 3 3 12 3 3 3 -100 3 3 4 3 3 7
3 3 3 3 3 3 4 3 -100 3 3 -100 3 4 3 10 4 3 3
3 8 4 -100 11 4 20 3 -100 3 10 3 3 4 3 3 3 3 3
3 5 3 4 -100
